# BRAFV600E-Associated Gene Expression Profile: Early Changes in the Transcriptome, Based on a Transgenic Mouse Model of Papillary Thyroid Carcinoma

**DOI:** 10.1371/journal.pone.0143688

**Published:** 2015-12-01

**Authors:** Dagmara Rusinek, Michal Swierniak, Ewa Chmielik, Monika Kowal, Malgorzata Kowalska, Renata Cyplinska, Agnieszka Czarniecka, Wojciech Piglowski, Joanna Korfanty, Mykola Chekan, Jolanta Krajewska, Sylwia Szpak-Ulczok, Michal Jarzab, Wieslawa Widlak, Barbara Jarzab

**Affiliations:** 1 Department of Nuclear Medicine and Endocrine Oncology, Maria Sklodowska-Curie Memorial Cancer Center and Institute of Oncology, Gliwice Branch, Gliwice, Poland; 2 Genomic Medicine, Department of General, Transplant and Liver Surgery, Medical University of Warsaw, Warsaw, Poland; 3 Department of Tumor Pathology, Maria Sklodowska-Curie Memorial Cancer Center and Institute of Oncology, Gliwice Branch, Gliwice, Poland; 4 Department of Oncological and Reconstructive Surgery, Maria Sklodowska-Curie Memorial Cancer Center and Institute of Oncology, Gliwice Branch, Gliwice, Poland; 5 Center for Translational Research and Molecular Biology of Cancer, Maria Sklodowska-Curie Memorial Cancer Center and Institute of Oncology, Gliwice Branch, Gliwice, Poland; 6 III Department of Radiotherapy and Chemotherapy, Maria Sklodowska-Curie Memorial Cancer Center and Institute of Oncology, Gliwice Branch, Gliwice, Poland; 7 II Department of Radiotherapy and Chemotherapy, Maria Sklodowska-Curie Memorial Cancer Center and Institute of Oncology, Gliwice Branch, Gliwice, Poland; IPATIMUP/Faculty of Medicine of the University of Porto, PORTUGAL

## Abstract

**Background:**

The molecular mechanisms driving the papillary thyroid carcinoma (PTC) are still poorly understood. The most frequent genetic alteration in PTC is the *BRAF*V600E mutation–its impact may extend even beyond PTC genomic profile and influence the tumor characteristics and even clinical behavior.

**Methods:**

In order to identify *BRAF*-dependent signature of early carcinogenesis in PTC, a transgenic mouse model with *BRAF*V600E-induced PTC was developed. Mice thyroid samples were used in microarray analysis and the data were referred to a human thyroid dataset.

**Results:**

Most of *BRAF*(+) mice developed malignant lesions. Nevertheless, 16% of *BRAF*(+) mice displayed only benign hyperplastic lesions or apparently asymptomatic thyroids. After comparison of non-malignant *BRAF*(+) thyroids to *BRAF*(−) ones, we selected 862 significantly deregulated genes. When the mouse *BRAF*-dependent signature was transposed to the human HG-U133A microarray, we identified 532 genes, potentially indicating the *BRAF* signature (representing early changes, not related to developed malignant tumor). Comparing *BRAF*(+) PTCs to healthy human thyroids, PTCs without *BRAF* and *RET* alterations and *RET*(+), *RAS*(+) PTCs, 18 of these 532 genes displayed significantly deregulated expression in all subgroups. All 18 genes, among them 7 novel and previously not reported, were validated as *BRAF*V600E-specific in the dataset of independent PTC samples, made available by The Cancer Genome Atlas Project.

**Conclusion:**

The study identified 7 *BRAF*-induced genes that are specific for *BRAF V600E*-driven PTC and not previously reported as related to *BRAF* mutation or thyroid carcinoma: *MMD*, *ITPR3*, *AACS*, *LAD1*, *PVRL3*, *ALDH3B1*, and *RASA1*. The full signature of *BRAF*-related 532 genes may encompass other *BRAF*-related important transcripts and require further study.

## Introduction

The *BRAF*V600E mutation resulting from the T1799A nucleotide substitution was first described by Davies et al. in 2002 in numerous cancers, with the highest frequency in melanoma [1]. A year later, Cohen et al. reported this mutation in a high proportion of papillary thyroid carcinomas (PTCs) [2]. Further studies confirmed that PTC is the second most frequent cancer type after melanoma harboring the *BRAF*V600E mutation, present in 45% of cases [3]. The majority of studies indicate on lack of coexistence between *BRAF* mutation and other molecular alterations described as frequent in PTCs, like *RET/PTC* rearrangements, *TRK* rearrangements and *RAS* mutations, suggesting that these are alternative events in PTC etiopathogenesis [4, 5].

*BRAF* encodes a serine–threonine kinase, which is a molecular signal transmitter within the RAS/BRAF/mitogen-activated extracellular signal-regulated kinase signaling pathway. The V600E mutation is responsible for *RAS*-independent constitutive BRAF kinase activation and an increase in ERK1/2 phosphorylation. The activity of the BRAFV600E kinase domain is about 500-fold higher than that of the wild-type BRAF. This high activity propagates through the MAPK pathway and pathways related to the deregulation of proliferation, differentiation, angiogenesis, inflammation, cell migration, and extracellular matrix (ECM) remodeling [6]. The recent study of Faustino et al. determined also over-activation of the AKT/mTOR pathway in *BRAF*(+) PTCs [7].

The *BRAF*V600E mutation plays a fundamental role in tumorigenesis and progression of PTC, as well as appears to be the primary initiating event [8]. This hypothesis is supported by numerous studies on cell lines and mouse models [9–11]. However, there are data contradicting this conclusion [12, 13]. Whether the *BRAF*V600E mutation initiates PTC or is a consequence of PTC remains to be elucidated. Nevertheless, the role of this mutation as a driver of PTC aggressiveness seems undeniable. Many studies have shown a significant association between the presence of the *BRAF*V600E mutation in PTC and factors characteristic of poor prognosis, such as extrathyroidal invasion, lymph node metastases, advanced disease stage, and lack of tumor capsule [14–16]. Moreover, Xing et al., in a multicenter study, documented the association of *BRAF*V600E with an increased cancer-related mortality among PTC patients [17], whereas Guerra et al. reported a correlation between higher allelic percentage of *BRAF*V600E mutation and poor disease outcome [18].

There have been several attempts to use specific inhibitors of mutated BRAF kinase, mostly in melanoma therapy; however, the results were not satisfactory [19, 20]. This reinforces the need to better understand the molecular consequences of *BRAF* mutation.

Several studies have investigated differences in the gene expression profile of *BRAF*-mutated PTCs compared with other PTCs [21–24], and have demonstrated a strong influence of the *BRAF*V600E mutation on the expression of genes related to angiogenesis, ECM remodeling, and the immune system. The Cancer Genome Atlas Research Network has recently reported genomic, epigenomic, and proteomic profile differences between *BRAF*-like and *RAS*-like PTCs, which suggests a need to reclassify thyroid cancers into molecular subtypes [25].

Nevertheless, the knowledge of the *BRAF* expression signature is still incomplete. The fact that human *BRAF*-induced PTC is usually at an advanced stage when diagnosed and the lack of knowledge about a possible pre-cancer stage of PTC makes distinguishing the cancer’s causes from its effects difficult. An additional issue constitutes a heterogeneous character of human material and a number of potential factors that may influence the results, including age, sex, other diseases, and environmental factors.

In this study, we focused on the influence of the *BRAF*V600E mutation on early PTC gene expression profile in a transgenic mouse model developed for this purpose.

## Material and Methods

### Ethics

All animal procedures were conducted in accordance with the recommendation of the Polish Council on Animal Care and were approved by the Local Committee of Ethics and Animal Experimentation at the Medical University of Silesia in Katowice, Poland and by the institutional animal care policy of the Maria Sklodowska-Curie Memorial Cancer Center and Institute of Oncology (Gliwice, Poland). The use of human tissue was approved by the Bioethics Committee of Maria Sklodowska-Curie Memorial Cancer Center and Institute of Oncology, Gliwice Branch. Written informed consent was obtained from all patients or caregivers for the use of their tissue for analysis in this study. All clinical data were anonymized and de-identified prior to the analysis.

### Engineering of the pTG-2HA-BRAFV600E construct and generation of transgenic mice

We used the commercially available plasmid pMEV-BRAF (Biomyx Technology, P1060d; San Diego, CA), which contains the human *BRAF*V600E cDNA in frame with sequence coding for the N-terminal affinity tag 2HA (hemagglutinin). To restrict expression of the transgene to thyroid follicular cells, the original cytomegalovirus promoter was replaced with the bovine thyroglobulin promoter, kindly provided by Prof. J. Dumont (Brussels). The thyroglobulin promoter was cloned upstream of the 2HA-BRAF into the *Spe*I/*Hin*dIII site, resulting in the pTG-2HA-BRAFV600E construct.

Transgenic mice were generated by microinjection of a purified *Apa*LI/*Pvu*II fragment into the pronuclei of zygotes from FVB/N females (AnimaLab, Poznan, Poland) by standard procedures. Mice were screened for the presence of the transgene by PCR using genomic DNA isolated from tail biopsies and primers complementary to the 2HA and *BRAF* coding sequences (F-5′-TTGCTAACGCAGTCAGTGCT-3′ and R-5′-CATGTCCCCGTTGAACAGA-3′, respectively). Mice with the transgene detected on the DNA level were classified as transgenic. Transgenic individuals were crossed with the wild-type FVB/N mice.

### Experimental animals

The animals (typically 2–5 animals per cage; minimum floor space per animal– 100 cm^2^) were kept under controlled environmental conditions with a 12L:12D cycle and were provided with food and tap water. Mice were under ocular inspection of their general health, and no differences were observed in the health between transgenic and non-transgenic individuals. The mice were killed by CO_2_ asphyxiation (at a mean age of 11 months) and the thyroid and lungs were resected. The thyroid was divided transversely so that each half contained fragments of both lobes. RNA was isolated from one half of the thyroid, and the remaining half of the thyroid and the lungs were subjected to histopathological evaluation.

### Detection of the transgene at mRNA level

RNA from the thyroids was isolated and treated with the DNase I (in order to remove genomic DNA contamination) using an RNeasy Micro Kit (Qiagen GmbH, Hilden, Germany) according to the manufacturer’s protocol. The concentration and quality of RNA were tested by spectrophotometry and evaluated on minichips (using NanoDrop ND-1000 and BioAnalyzer 2100, respectively; Agilent Technologies, Santa Clara CA, United States). RNA (25 ng) with an RNA Integrity Number (RIN) factor >8 was converted to cDNA with the Sensiscript RT Kit (Qiagen GmbH).

PCR on cDNA template (as well as on RNA template as a control for genomic contamination) was performed with primers spanning the 15^th^ exon of the *BRAF* gene with the analyzed V600 codon: F-5′-acacgccaagtcaatcatcc-3′ and R-5′-tctggtccctgttgttgatg-3′. The PCR product was analyzed using Sanger’s direct sequencing method on a 3130xl Genetic Analyzer Applied Biosystems (Life Technologies, Carlsbad CA, USA) in order to confirm the presence of the *BRAF*V600E at the RNA level (S1 Fig). All mice that expressed the transgene in thyroid at the RNA level were classified as *BRAF*(+).

### Detection of the transgene expression at the protein level

For Western blotting, extracts of mouse thyroids fragments were prepared with the RIPA buffer (50mM Tris-HCl pH 7.5, 150 mM NaCl, 50mM NaF, 1mM DTT, 0.1% NP40, 1mM PMSF, 1x Complete Roche inhibitor) and protein concentration was determined using the Bradford assay (Bio-Rad, Hercules, CA, USA). For a positive control cellular proteins were extracted from NIH3T3 cells 48 hours after transient transfection with pMEV-2HA/BRAF vector. Equal amounts of proteins (40 μg) were separated on 4–8% SDS-PAGE gels and transferred to 0.45-μm pore nitrocellulose filter (Millipore, Bedford, MA, USA). The membranes were blocked with 5% of nonfat milk in TTBS (250mM Tris-HCl pH 7.5, 0.1% Tween-20, 150mM NaCl) for 1h at room temperature. Then the membranes were incubated overnight at 4°C with primary antibodies against HA (1:2500; rabbit polyclonal HA-tag, PAB10343, Abnova, Taipei, Taiwan) or actin (1:2000; mouse monoclonal, clone C4, MAB1501R, Merck Millipore, Darmstadt, Germany). Proteins were visualized after incubation with peroxidase-conjugated secondary antibody using enhanced chemiluminescence (SuperSignal West Pico Chemiluminescent Substrate; Pierce Thermo Fisher Scientific, Illinois, USA) according to the manufacturer protocol.

For immunohistochemistry, mouse thyroids were fixed overnight in 10% buffered formaldehyde at 4°C, washed in phosphate-buffered saline (PBS) at 4°C, dehydrated, paraffin-embedded, and sectioned (6 μm). For detection of the 2HA/BRAF fusion protein, monoclonal, rabbit anti-HA antibody (Cell Signaling Technology, Boston, MA, USA; Cat no 3724S) was employed (1:500, overnight, 4°C). An antigen retrieval step in 0.01 M citrate buffer pH 6.0 was performed before immunohistochemistry procedure with an ImmPRESS™ anti-rabbit Ig (peroxidase) reagent (Vector Laboratories; Burlingame, CA, USA) according to the manufacturer’s guidelines. 3,3’-Diaminobenzidine (DAB) was used as a chromogen for visualization of immunohistochemical reactions and hematoxylin was used for counterstaining. Negative controls were performed in parallel for specific labelling by omitting the primary antibody.

### Microarray-derived dataset

The microarray analysis included both mouse and human material. The human material was derived from our cohort of PTC patients, together with microarray data from the cohort of patients with PTC published by Giordano et al. [22]. The distribution of samples used in analysis is shown in Tables 1 and 2.

**Table 1:** Microarray-derived dataset- mouse cohort.

Type of thyroid lesion	Total number of cases	Number of cases in relation to the age (and tg line)		
		4–8 months	9–12 months	>12 months
Papillary thyroid carcinomas	10 *BRAF*(+)	3 (tg2)	2 (tg3)	5 (tg3)
Borderline thyroid lesions	10 *BRAF*(+)	1 (tg2)	4 (tg3)	1 (tg2); 4 (tg3)
Benign hyperplastic thyroid lesions	4 *BRAF*(+)	1 (tg3)	3 (tg3)	
	4 *BRAF*(-)	1 (tg2)	1 (tg2); 1 (tg3)	1 (tg1)
Asymptomatic thyroids	5 *BRAF*(+)	1 (tg2)	4 (tg3)	
	5 *BRAF*(-)	1 (tg2)	2 (tg2); 2 (tg3)	

*BRAF*(+)–mice positive for the *BRAF*V600E at the RNA level; *BRAF*(-)–mice negative for the *BRAF*V600E at the RNA level and genotyped as non-transgenic [except *BRAF*(-) tg1 mouse which was genotyped as transgenic]

**Table 2:** Microarray-derived dataset- human cohort.

Type of thyroid lesion	Number of cases (Gliwice cohort)	Number of cases (Giordano et al. 2005)
Papillary thyroid carcinomas	18 *BRAF*(+)	23 *BRAF*(+)
	8 *RET*(+)	5 *RET*(+)
	1 *RAS*(+)	5 *RAS*(+)
		8 PTC(-)
Apparently healthy thyroids	18	0

PTC(-)- PTCs without any mutation detected

The mouse cohort consisted of 38 mice thyroid samples: 10 PTCs; 10 borderline thyroid lesions; 8 benign hyperplastic thyroid lesions, and 10 asymptomatic mice thyroid glands. Most cases of *BRAF*(+) mice used in the microarray analysis were from the tg3 line, since within this line the highest incidence of the pre-cancerous PTC stages was observed what correlated with a lower than in tg2 transgene expression at the protein level (see Results section).

Our human cohort included material obtained from thyroid cancer patients who underwent surgery at MSC Memorial Cancer Center and Institute of Oncology, Gliwice Branch, Poland. This material consisted of 27 PTCs and 18 apparently healthy thyroid glands. We completed our microarray analysis of human material by the data made available by Giordano et al. [22] consisting of 41 PTCs.

### Validation

Validation was performed using the data made available by The Cancer Genome Atlas Project [25], in total 391 PTC samples, including 228 *BRAF*(+), 23 *RET*(+), 52 *RAS*(+), and 88 PTC(−) samples (in this set of PTCs, all *RET* rearrangements, detected by the authors, are considered as *RET*(+) samples). Noteworthy PTCs without any mutation detected are named PTC(-).

### Microarray analysis

The GeneChip Mouse Gene 1.0 ST and Human Genome 133A Affymetrix arrays (Santa Clara CA, USA) were used to analyze the samples from mice and human thyroids, respectively. Total RNA (100 ng mouse or 5 μg human thyroid specimens) was the template for synthesis of double-stranded cDNA, followed by transcription together with cRNA biotinylation, cRNA fragmentation, and hybridization of cRNA to the arrays. The arrays were then washed in the presence of streptavidin–phycoerythrin complex and scanned on a GeneChip 3000 (Affymetrix, Santa Clara CA, USA). The whole procedure was performed according to the manufacturer’s protocol.

### Analysis of the microarray data

Raw data from the mouse cohort were pre-processed using the robust multi-array average method (RMA), followed by filtering out control and “flat” (less than 20% of samples with at least 1.5-fold change in either direction from the median) probe sets. Three-way ANOVA was performed on non-malignant samples (8 benign hyperplastic thyroid lesions and 10 asymptomatic thyroid glands) to select probe sets significantly deregulated between *BRAF*(+) and *BRAF*(−), tissue status, and sex of mice.

Raw data from both human cohorts were normalized independently using the robust multi-array average method with help of probe sequences (GCRMA). Cohort-specific bias was removed by batch mean-centering correction [26]. A hypergeometric test was performed among the selected genes to estimate overrepresentation of pathways from the KEGG (Kyoto Encyclopedia of Genes and Genomes) PATHWAY Database. Probe sets significantly deregulated between analyzed groups of samples were selected using a *t* test for pairwise comparisons between *BRAF*(+) and *RET*(+) PTCs, *RAS*(+) PTCs, PTCs(−), and normal thyroid samples, independently. The Benjamini–Hochberg false discovery rate (FDR) was used to assess the multiple testing errors.

Unsupervised analysis was performed by hierarchical clustering using a ward agglomeration method, which operates on Euclidean distance measures.

The research version of thyroid differentiation score (TDS) was calculated on the mice microarray normalized data according to the algorithm presented in The Cancer Genome Atlas Project [25]. In analysis of two group differences, U Mann-Whitney test was used. In multi-group comparisons the nonparametric Kruskal-Wallis method with post-hoc Steel-Dwass test was applied.

The data discussed in this publication have been deposited in NCBI’s Gene Expression Omnibus [27] and are accessible through GEO Series accession number GSE58689 (http://www.ncbi.nlm.nih.gov/geo/query/acc.cgi?acc=GSE58689).

### Analysis of the validation dataset

The normalized results for gene expression (RNA-Seq Version2) were downloaded from The Cancer Genome Atlas (TCGA) Data Portal (https://tcga-data.nci.nih.gov/tcga/). Expression of 18 genes selected as *BRAF*(+)-specific in the microarray analysis was analyzed by performing a Mann–Whitney test for pairwise comparisons between *BRAF*(+) and *RET*(+) PTCs, *RAS*(+) PTCs, and PTCs(−), independently. Results were corrected by the FDR control.

**Fig 1:**
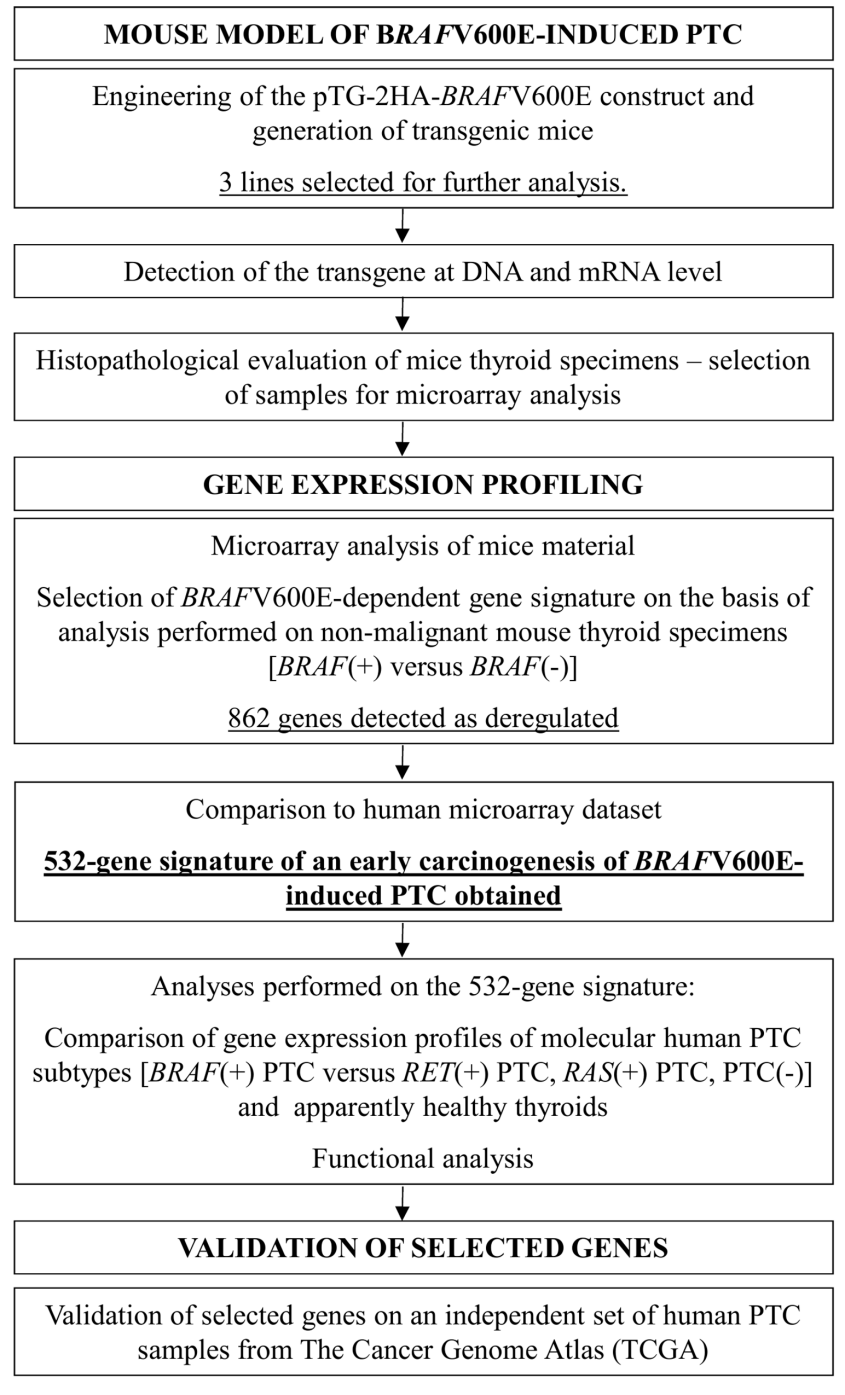
General scheme of the study.

For the general scheme of the study, refer to Fig 1.

## Results

### Transgenic mice

To investigate changes in the transcriptome of the *BRAF*V600E-induced PTC we developed a transgenic mouse model with mutated BRAF expression targeted to thyroid cells. Six of 19 mice born after microinjection showed integration of the transgene on the DNA level (1 female and 5 males). These original TG-BRAFV600E founders were used to establish 6 transgenic lines. After an initial analysis of the transgene expression and histopathological evaluation of thyroid specimens representing all lines, 3 lines were selected for further experiments and characterized in detail (tg1, tg2, and tg3). Transgenic mice accounted for 76% of all mice analyzed (89/117). The sequencing of cDNA generated from thyroid glands of 4–23-month-old transgenic mice revealed the presence of the human *BRAF*V600E mutation (S1 Fig) in 72% (64/89) mice primarily genotyped as transgenic (excluding the founders) (see also S1 Table).

#### Histopathological evaluation of mice thyroids and BRAFV600E protein expression

PTC was identified in 61% (39/64) mice that expressed mutated *BRAF*. Although in their thyroids there were some features typical of human PTC, such as enlarged, overlapping nuclei with grooves and pseudoinclusions, the general histopathological image of *BRAF*V600E-induced mice PTC differed from the human one. The mouse PTCs were of classical PTC variant, including areas of follicular architecture (S2 Fig). The cribriform variant of PTC occurred in 23% of cases (9/39), whereas prominent hobnail features were noticed in 8% of cases (3/39). However, neoplastic changes were not found in thyroid glands of all *BRAF*(+) mice. In 8% of all *BRAF*(+) mice, features of benign hyperplastic lesions were recognized (5/64; S2 Fig), and in a further 9% (6/64), histopathological evaluation showed no visible changes in the thyroid (asymptomatic thyroid).

In thyroids of a significant fraction of *BRAF*(+) mice, the histopathological analysis revealed changes in nuclei that classified these cases as not healthy but not yet PTC (S2 Fig). They were characterized by the presence of single nuclei with grooves or pseudoinclusions, heterogeneous nuclei, and separation of altered cells in single foci. These lesions, which we call borderline, were described in 22% of cases (14/64). In *BRAF*(+) mice with fully developed PTC, we observed metastases to lungs and extrathyroidal invasion to muscles with infiltration of adjacent fat and connective tissue in 15% (6/39) and 5% (2/39) mice, respectively (S3 Fig). The data concerning the histopathological analysis of all mice were summarized in the S1 Table.

The analyzed transgenic lines differed between themselves in the incidence of PTC as well as borderline lesions what generally correlated with the level of the BRAFV600E protein (see S4 Fig). The tg2 line was characterized by the highest level of the BRAFV600E protein and the highest frequency of papillary thyroid carcinomas with the 76% of cases (19 out of 25 *BRAF*(+) mice). The incidence of PTC in tg3 line (with a lower level of the BRAFV600E protein) reached 50% of *BRAF*(+) mice (19/38). However, within this line, mice displayed borderline thyroid lesions and non-malignant hyperplastic lesions more frequently comparing to tg2 individuals. Thus, the weaker expression of mutated BRAF kinase within the tg3 line translates into less PTC cases and more pre-cancerous stages incidence.

Within the tg1 line we identified only one *BRAF*(+) mouse and this mouse displayed PTC. All other mice within this line genotyped as transgenic did not express the transgene at the RNA level (S1 Table) what suggested that in the progeny the transgene could become methylated. Consistently, at the protein level we found BRAFV600E only in PTC case (S4 Fig).

### Microarray analysis

#### Results obtained by analysis on mouse cohort

On microarrays we analyzed 10 cases of PTC and 10 cases of borderline thyroid lesion [all *BRAF*(+)] as well as a non-malignant mouse thyroid samples: 8 benign hyperplastic thyroid lesions among them 4 were *BRAF*(+) and 4 *BRAF*(−), and 10 asymptomatic thyroids, with 5 being *BRAF*(+) and 5 *BRAF*(−). To identify a *BRAF*V600E signature for early carcinogenesis of PTC we focused on non-malignant samples (18 out of 38 arrayed). We compared global changes in a gene expression in *BRAF*(+) and *BRAF*(−) cases. In addition to the presence of the *BRAF*V600E mutation, we considered sex and histological type of material (benign hyperplastic lesion or apparently asymptomatic thyroids).

**Fig 2:**
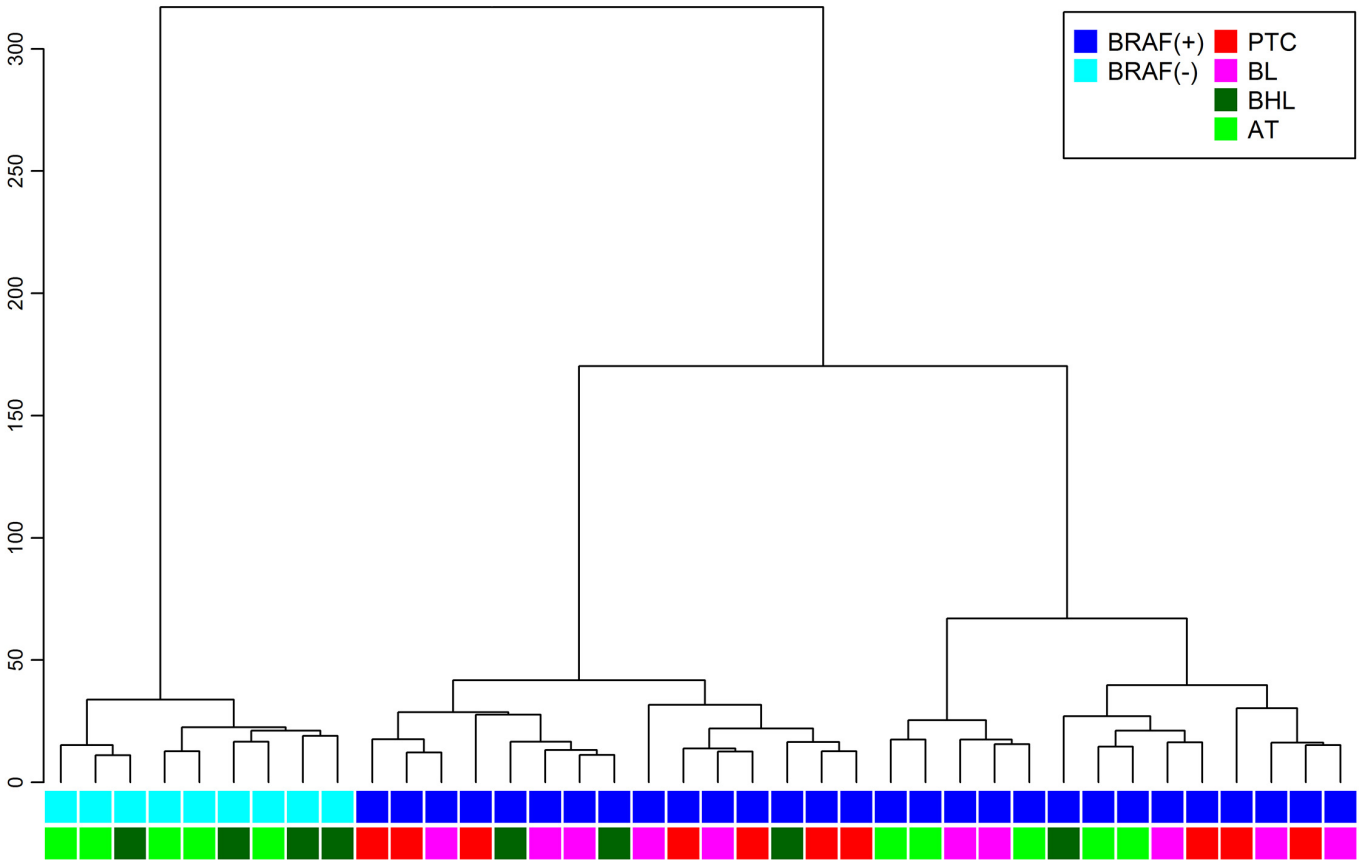
Hierarchical clustering of mouse samples. Thirty-eight mouse samples based on 1020 probe sets significantly differentiating between *BRAF*(+) and *BRAF*(−) non-malignant mouse samples (marked with blue and cyan respectively). PTCs (red); borderline thyroid lesions (BL; magenta); benign hyperplastic thyroid lesions (BHL; dark green); asymptomatic thyroid glands (AT; green).

We detected 1020 probe sets (corresponding to 862 known genes) that were significantly deregulated (FDR < 0.05) in *BRAF*(+) compared with *BRAF*(−) non-malignant mice thyroid samples, adjusting for type of material (no probe sets with FDR < 0.05) and sex (114 probe sets with FDR < 0.05) in the three-way ANOVA. To confirm the signature is *BRAF*V600E-specific, hierarchical clustering was performed for all 38 microarrayed mouse samples, and resulted in perfect separation between the *BRAF*(+) and *BRAF*(−) samples, irrespective of material histopathological type (Fig 2).

#### Results obtained by analysis on human cohort

**Fig 3:**
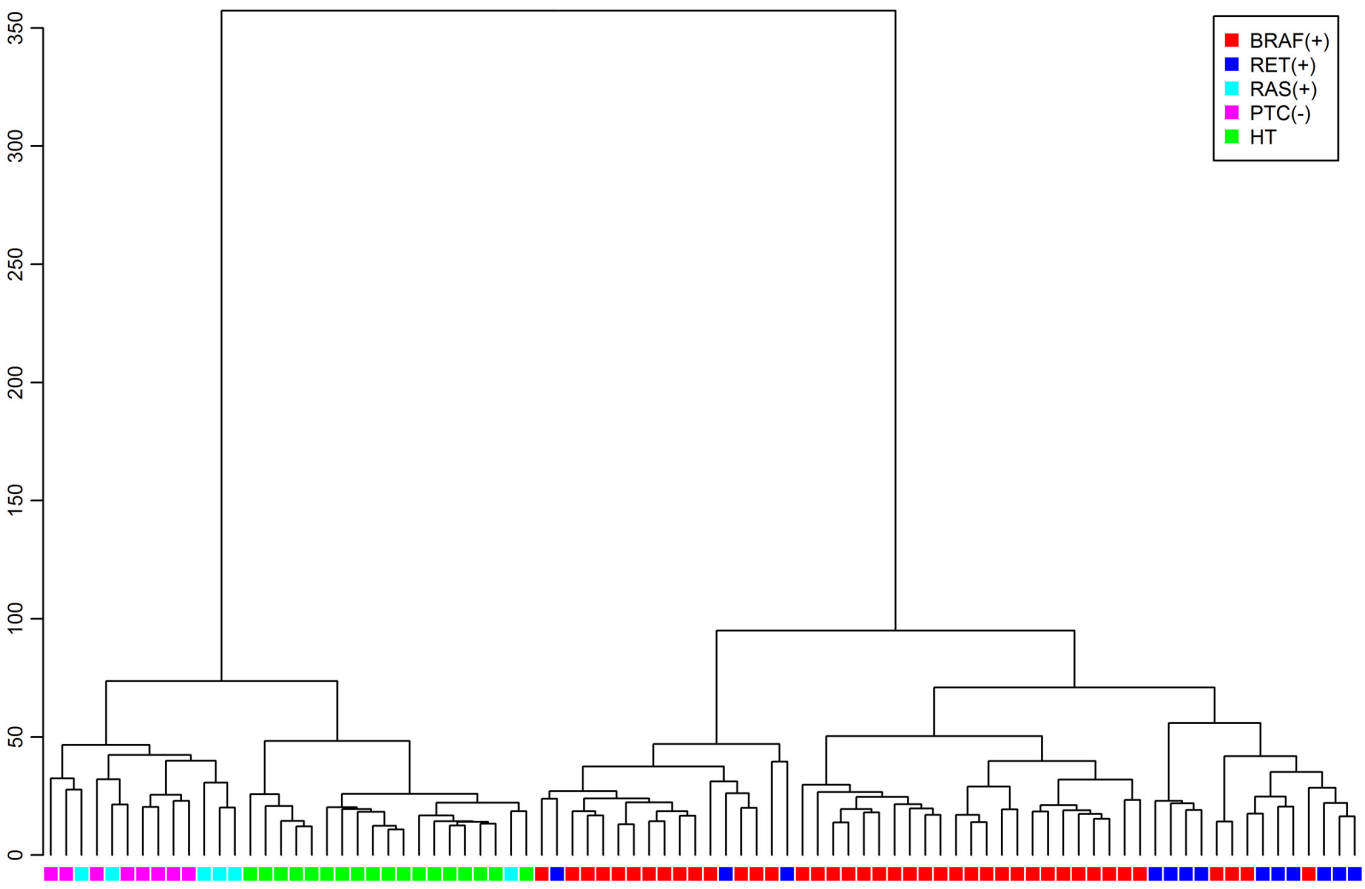
Hierarchical clustering of human samples. Eighty-six human samples based on 532 of 862 genes selected on the mice dataset and identified on the human arrays. *BRAF*(+) PTCs (red); *RET*(+) PTCs (blue); *RAS*(+) PTCs (cyan); PTCs(−) (magenta); apparently healthy thyroid samples (HT; green).

The early *BRAF*V600E signature obtained from the mouse microarray data analysis was evaluated on the human microarray-derived dataset. From 862 genes selected on the mouse dataset as *BRAF*V600E-specific for early PTC, 532 were identified on the human HG-U133A microarray (full results in S2 Table). To verify the global utility of this signature on the human samples, hierarchical clustering based on these 532 genes was performed, revealing that the main source of variability in the human dataset was the difference between *BRAF*(+) or *RET*(+) PTCs and all remaining samples. Even when *RAS*(+) and PTCs(−) samples were clearly separated from apparently healthy thyroid samples, they were much closer to them than to *BRAF*(+) or *RET*(+) PTC samples (Fig 3).

The functional analysis within these 532 genes showed the overrepresentation of 23 signaling pathways (*P* < 0.05, odds ratio > 1.5). Five out of 23 pathways are involved in metabolism. However, among the overrepresented pathways are pathways related to the endocrine system, signal transduction, and some associated with different types of cancer (Table 3).

**Table 3:** Pathway analysis of the 532-gene signature of BRAFV600E-induced PTC early carcinogenesis.

Pathway Name	P-value	Odds Ratio	Genes
Calcium signaling pathway	4,7E-04	2,6	*ADCY3*, *ADRA1A*, *ADRB3*, *BST1*, *CALML3*, *CYSLTR1*, *EDNRA*, *ERBB3*, *ERBB4*, *ITPR3*, *OXTR*, *P2RX6*, *PHKG1*, *PLCB4*, *PLCG2*, *PLN*, *PTAFR*, *PTGER3*, *PTK2B*, *TACR3*
Salivary secretion	7,1E-04	3,4	*ADCY3*, *ADRA1A*, *ADRB3*, *ATP1A2*, *BST1*, *CALML3*, *DMBT1*, *GUCY1A2*, *ITPR3*, *PLCB4*, *SLC12A2*, *TRPV6*
Osteoclast differentiation	2,5E-03	2,5	*BTK*, *FCGR2B*, *FHL2*, *IL1B*, *LILRA6*, *LILRB4*, *MAPK10*, *NCF2*, *NCF4*, *PIK3CG*, *PLCG2*, *PPARG*, *SOCS3*, *SYK*, *TYROBP*
Fatty acid biosynthesis	2,8E-03	17,6	*ACACA*, *ACACB*, *FASN*
Propanoate metabolism	3,4E-03	4,8	*ABAT*, *ACACA*, *ACACB*, *ACSS3*, *EHHADH*, *SUCLA2*
Malaria	3,4E-03	3,6	*COMP*, *ICAM1*, *IL18*, *IL1B*, *MET*, *SDC1*, *SELP*, *THBS2*
Insulin signaling pathway	3,7E-03	2,4	*ACACA*, *ACACB*, *BRAF*, *CALML3*, *FASN*, *FBP1*, *LIPE*, *MAPK10*, *PCK1*, *PHKG1*, *PIK3CG*, *PRKAR2B*, *PYGM*, *SLC2A4*, *SOCS3*
ECM-receptor interaction	4,5E-03	2,8	*AGRN*, *CD44*, *COL5A2*, *COMP*, *FN1*, *ITGA4*, *LAMB1*, *SDC1*, *SPP1*, *SV2A*, *THBS2*
Fatty acid elongation in mitochondria	4,7E-03	13,2	*ACAA2*, *HADH*, *HADHB*
Glycerolipid metabolism	5,2E-03	3,8	*AGPAT2*, *DGKB*, *LPL*, *MBOAT2*, *MGLL*, *PNPLA3*, *PPAP2A*
PPAR signaling pathway	6,5E-03	3	*ACSL3*, *ADIPOQ*, *EHHADH*, *FABP4*, *LPL*, *PCK1*, *PLIN1*, *PPARG*, *RXRG*
Adipocytokine signaling pathway	7,2E-03	2,9	*ACACB*, *ACSL3*, *ADIPOQ*, *MAPK10*, *NPY*, *PCK1*, *RXRG*, *SLC2A4*, *SOCS3*
Alzheimer's disease	1,0E-02	2,1	*BACE2*, *CALML3*, *COX5A*, *COX6A1*, *IL1B*, *ITPR3*, *LPL*, *MME*, *NDUFA1*, *NDUFB6*, *NDUFB8*, *PLCB4*, *SDHB*, *UQCR11*, *UQCRQ*
Arachidonic acid metabolism	1,1E-02	3,2	*CYP2E1*, *EPHX2*, *GGT5*, *GPX2*, *GPX3*, *PTGS1*, *PTGS2*
Butanoate metabolism	1,4E-02	4	*AACS*, *ABAT*, *ACSM3*, *EHHADH*, *HADH*
Thyroid cancer	1,9E-02	3,7	*BRAF*, *CCND1*, *NCOA4*, *PPARG*, *RXRG*
Leukocyte transendothelial migration	2,8E-02	2,1	*ACTN1*, *CLDN6*, *CXCL12*, *ICAM1*, *ITGA4*, *ITGAM*, *NCF2*, *NCF4*, *PIK3CG*, *PLCG2*, *PTK2B*
Proximal tubule bicarbonate reclamation	2,9E-02	3,9	*AQP1*, *ATP1A2*, *PCK1*, *SLC38A3*
Phosphatidylinositol signaling system	3,5E-02	2,3	*CALML3*, *DGKB*, *INPP5J*, *ITPR3*, *PIK3CG*, *PIP5K1B*, *PLCB4*, *PLCG2*
Glioma	4,0E-02	2,4	*BRAF*, *CALML3*, *CCND1*, *CDK6*, *IGF1R*, *PIK3CG*, *PLCG2*
Staphylococcus aureus infection	4,0E-02	2,6	*CFD*, *FCGR2B*, *ICAM1*, *ITGAM*, *PTAFR*, *SELP*
Cytokine-cytokine receptor interaction	4,8E-02	1,6	*BMP7*, *CCL17*, *CCL7*, *CCR2*, *CNTFR*, *CSF2RB*, *CXCL11*, *CXCL12*, *EDA2R*, *IL10RA*, *IL17RB*, *IL18*, *IL18R1*, *IL1B*, *INHBA*, *INHBB*, *MET*, *OSMR*, *TNFRSF21*
Cardiac muscle contraction	5,0E-02	2,2	*ATP1A2*,*CACNA2D1*, *CACNA2D2*, *COX5A*, *COX6A1*, *UQCR11*, *UQCRQ*

Considering the molecular event (presence of *BRAF*V600E mutation, *RET* rearrangements, and *RAS* mutation) of the available human PTCs, from these 532 genes, we selected genes significantly different (FDR < 0.05) between *BRAF*(+) PTCs and *RET*(+) PTCs (50 genes), *RAS*(+) PTCs (135 genes), PTCs(−) (146 genes), and healthy thyroid samples (220 genes). 290 out of 532 transcripts (54.5%) showed significant difference in at least one comparison. Eighteen genes were deregulated in all 4 comparisons (13 upregulated and 5 downregulated in *BRAF*(+) PTCs; Table 4) (S2 Table, S5 Fig). Among the 18 genes deregulated in a *BRAF*V600E-specific manner, 7 had not previously been considered as associated with either thyroid cancer or the *BRAF* mutation. These new genes are *PVRL3*, *MMD*, *LAD1*, *ITPR3*, *ALDH3B1*, *RASA1*, and *AACS*. When the expression of these genes (previously derived in mouse benign samples and then further selected on human PTC/normal samples) was analyzed backward in mouse dataset including mouse PTCs and borderline lesions, not used at the step of gene selection, all of them showed the direction of change in mouse PTC identical to human PTC, and this trend was also observed in borderline lesions (S3 Table). These differences were statistically significant for all genes in comparison of borderline lesions and PTCs to *BRAF*(-) healthy thyroids.

**Table 4:** Genes significantly deregulated in human *BRAF*V600E-induced PTC samples.

Comparisons between *BRAF*(+) PTCs and:	Microarrays included in the study								The Cancer Genome Atlas					
	*RET*(+)^a^		*RAS*(+)		PTC (-)		healthy thyroid		*RET*(+)^b^		*RAS*(+)		PTC (-)	
Symbol	FDR	Fold change	FDR	Fold change	FDR	Fold change	FDR	Fold change	FDR	Fold change	FDR	Fold change	FDR	Fold change
DCSTAMP*	1,1E-09	24,57	1,1E-14	181,51	1,2E-16	162,14	1,2E-23	61,86	2,6E-12	8,07	2,9E-28	58,04	3,4E-39	20,92
***MMD***	1,4E-04	0,43	4,0E-07	0,19	1,2E-04	0,41	5,9E-05	0,54	1,2E-04	0,64	4,8E-19	0,42	1,8E-11	0,56
*SLC34A2*	1,9E-04	3,16	1,4E-14	62,24	3,9E-09	19,86	1,2E-23	31,99	1,2E-09	4,47	3,1E-27	17,82	3,6E-33	6,95
FN1*	1,7E-03	5,58	6,0E-09	62,78	2,5E-08	44,84	3,3E-13	18,87	2,4E-05	2,07	2,9E-28	47,55	2,0E-34	7,95
*PLAUR*	2,2E-03	0,28	8,7E-03	3,67	1,1E-03	3,98	3,5E-04	2,92	1,1E-01	0,70	2,0E-22	6,07	1,5E-19	2,30
***ITPR3***	5,4E-03	1,7	6,7E-03	2,06	2,6E-04	2,17	2,6E-08	2,24	2,4E-06	1,69	9,6E-08	1,45	1,8E-22	1,94
*GRB7*	5,9E-03	1,76	1,1E-02	2,03	1,1E-02	1,91	1,1E-07	2,23	5,6E-08	1,76	3,1E-27	3,47	4,5E-33	3,07
*PDLIM4*	6,2E-03	1,67	8,8E-18	16,73	2,7E-14	12,63	6,5E-28	10,69	5,6E-08	1,84	2,9E-28	11,50	2,4E-35	4,79
MET*	6,4E-03	2,04	2,4E-07	7,68	4,1E-06	4,49	4,6E-19	9,09	1,5E-03	1,35	2,9E-28	4,82	1,6E-31	3,18
***AACS***	9,3E-03	0,67	8,8E-06	0,44	2,4E-06	0,43	3,9E-06	0,59	3,9E-02	0,92	1,0E-22	0,62	2,2E-22	0,66
***RASA1***	1,7E-02	1,45	2,5E-04	1,95	1,8E-05	1,96	3,3E-11	2,14	1,5E-03	1,23	4,6E-21	1,95	1,0E-25	1,89
***LAD1***	1,8E-02	1,73	1,1E-03	2,53	1,2E-04	2,75	1,5E-11	3,22	5,6E-08	1,87	7,4E-25	2,79	1,6E-34	3,28
*IQGAP2*	2,1E-02	0,46	2,3E-08	0,1	2,1E-04	0,24	3,3E-12	0,19	2,6E-03	0,47	3,6E-23	0,22	4,3E-29	0,20
ERBB3*	2,9E-02	2,5	9,7E-05	5,2	5,8E-03	2,91	9,7E-10	5,13	5,6E-08	1,89	3,2E-28	5,05	2,0E-34	3,66
***PVRL3***	3,0E-02	0,45	4,9E-03	0,31	8,0E-05	0,23	5,3E-08	0,32	1,7E-05	0,49	3,2E-10	0,45	2,0E-05	0,65
EPHA2*	3,9E-02	0,61	1,7E-04	2,84	4,1E-06	3,19	5,4E-10	2,98	2,1E-02	0,84	2,9E-18	2,00	5,2E-17	1,68
DIO1*	4,2E-02	0,24	5,5E-07	0,01	4,8E-05	0,03	1,8E-15	0,02	1,6E-03	0,56	2,2E-23	0,06	1,0E-27	0,05
***ALDH3B1***	4,8E-02	1,53	2,1E-02	1,89	2,9E-02	1,69	1,3E-06	2,07	1,5E-03	1,27	1,3E-23	3,57	3,9E-19	2,09

Abbreviations: FDR = Benjamini-Hochberg false discovery rate.

*RET*(+)^a^-PTCs with detectable *RET/PTC1* or *RET/PTC3* rearrangements

*RET*(+)^b^-PTCs with detectable *RET* rearrangements.

Genes not previously reported as related to thyroid cancer or *BRAF*V600E mutation are bolded

Genes with documented relation to thyroid cancer or to *BRAF*V600E mutation are underlined and marked by asterisk respectively.

### Comparison with the Cancer Genome Atlas data

**Fig 4:**
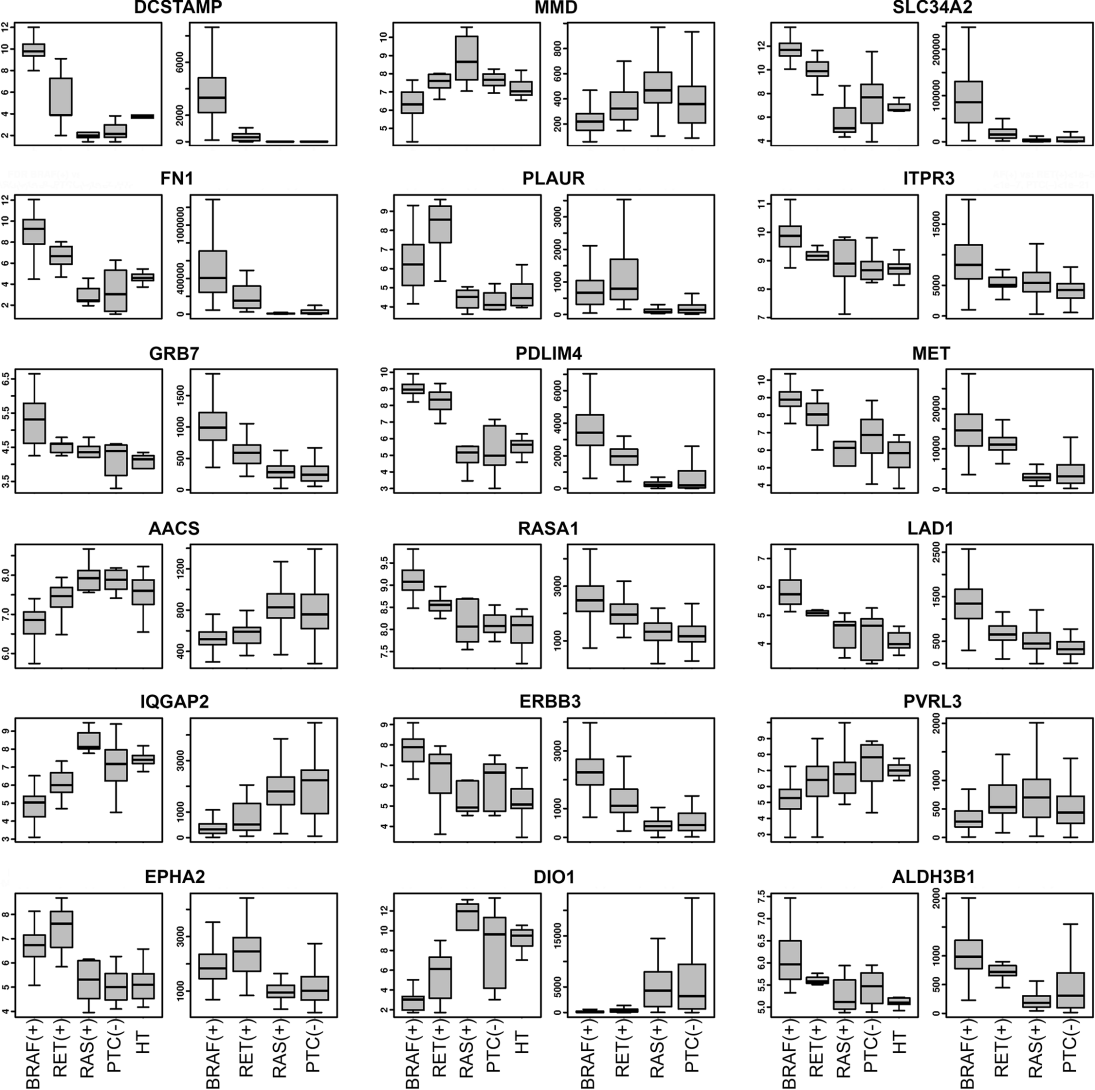
Boxplots of 18 genes chosen for validation. Expression distribution for each gene from our microarray data (on the left), The Cancer Genome Atlas Project data (on the right). The expression levels of analyzed genes are presented in *BRAF*(+) PTCs, *RET*(+), *RAS*(+), PTC(-) and healthy thyroids (HT) from left to right, respectively (as presented at the bottom of the figure). FDR values are shown in Table 4.

Eighteen genes selected in our microarray analysis were validated on the data from The Cancer Genome Atlas Project. *BRAF*V600E-specific altered expression was fully confirmed for 17 out of 18 genes (FDR < 0.05 for all comparisons, with changes of expression all in the same direction) (Table 4, Fig 4). The only gene not completely confirmed was *PLAUR*, for which expression of *RET*(+) PTCs was not significantly changed (FDR = 0.11) compared with *BRAF*(+) samples.

As in TCGA study the novel measure of thyroid specific genes expression named TDS (thyroid differentiation score) was introduced, we calculated this metric also in our mice data. According to expectations, PTCs exhibited lower TDS than asymptomatic thyroids (p value in Kruskal-Wallis test p = 0.01, p value in post-hoc test p = 0.007 for comparison between PTC vs BRAF(-) normal/benign lesions; S6 Fig). Conversely, no pronounced difference between normal/benign lesions of *BRAF*(+) vs *BRAF*(-) mice was seen, indirectly indicating that no major change in TSH level occurred between these groups, although a slight and non-significant decrease in *BRAF*(+) specimens was observed in comparison to *BRAF*(-) ones.

## Discussion

### Summary of the results

In our study, we analyzed mouse thyroid tumors driven by *BRAF*V600E transgene. The majority of mice with high expression of BRAF protein developed papillary thyroid cancer, while in lines with less abundant protein, PTC cases were less frequent. Especially important, cases with less advanced lesions (borderline or benign hyperplastic) were observed more frequently in mouse line with lower protein amount. In genomic analysis, we identified 532 transcripts potentially influenced by *BRAF* status. Forty one percent of these genes showed significant alterations in expression in human PTC. Of particular interest, 18 *BRAF*-specific transcripts (showing differences vs PTCs driven by other mutations) demonstrated deregulated expression also in primary non-malignant lesions, potentially indicating their involvement in early stages of tumorigenesis.

### *BRAF* transgenic PTC model

Published data indicate that *BRAF*V600E mutation is an initiating event in the development of PTC and is associated with more aggressive disease [28]. Extensive study of the molecular consequences of this mutation revealed significant effects on the gene expression profile. However, most analyses have been performed on human thyroid material, which is characterized by a large heterogeneity with many potential confounding factors that may affect the results, such as age, sex, clinical and environmental variables, and additional diseases. Moreover, it is very challenging to study the initial molecular consequences of the *BRAF*V600E mutation in human early lesions (lack of access). In our study, we developed a mouse model of PTC induced by the *BRAF*V600E mutation in order to analyze the molecular consequences of this alteration *in vivo* in more homogeneous material and to focus on the early stages of the neoplastic transformation.

### Histopathology of *BRAF*V600E-induced PTC

The question arises whether the transgenic mouse PTC resembles the human counterpart. In our model, mouse PTCs had number of features typical for human PTCs, such as nuclear grooves; pseudoinclusions; and enlarged, heterogeneous, and overlapping nuclei. These features lead to the pathological pattern less pronounced than in human PTC (personal opinion of pathologist involved in the study, E.C). However, in addition to the typical human *BRAF*(+) PTC classical variant, most mouse PTCs had an admixture of a follicular architecture, which is normally rare in PTCs associated with *BRAF* mutations [29]. In single mice, we also observed features of the cribriform–morular PTC subtype, which in humans occurred only in 1–2% of cases of familial adenomatous polyposis and was uncommon in PTC [30, 31]. Furthermore, the *BRAF*V600E mutation has never been detected in patients with this variant of PTC [32, 33]. However, the molecular background of the cribriform–morular PTC variant includes Wnt pathway activation with altered β-catenin (*CTNNB1*) expression. A relation between the presence of the *BRAF*V600E mutation, change in *CTNNB1* expression, and consequent activation of the Wnt signaling pathway has been shown in sessile serrated colorectal adenomas and sessile serrated adenomas dysplasia, as well as in skin melanoma [34, 35].

Since Knauf et al. has developed in 2005 the first mouse model of *BRAF*V600E-induced papillary thyroid carcinoma [9], several *in vivo* studies of *BRAF*(+) PTC have been published. The first transgenic mouse model demonstrated the initiating *BRAF*V600E potential as well as aggressive nature of *BRAF*-induced PTC. Using the same model 6 years later the authors performed microarray analysis of paired mouse PDTC and PTC and found deregulation of genes involved in cell adhesion and intracellular junctions, with changes consistent with a TGFβ-mediated epithelial-mesenchymal transition (EMT) [36]. Further *in vivo* studies confirmed the role of mutated *BRAF* in PTC progression through genes related to tumor invasion and metastasis [37] and demonstrated the key role of TSH signaling in *BRAF*-induced PTC initiation [38] and its relation to more aggressive features of *BRAF*(+) PTCs [39]. Although there were studies in which hyperplastic thyroid lesions preceding malignancy were observed in *BRAF*(+) mice [40], these were never analyzed. Our mouse model is unique against models published so far since next to hyperplastic lesions a part of *BRAF*(+) transgenic mice developed borderline thyroid lesions, while some individuals exhibited asymptomatic thyroids despite the presence of the transgene. We are also the first who used mouse model to analyze early PTC stages and transposed these results to human data. We observed significant *BRAF*V600E-dependent changes in the gene signature of *BRAF*(+) apparently asymptomatic thyroids and benign hyperplastic thyroid lesions compared with *BRAF*(−), despite the lack of clear differences in the histopathological examination. Thus, we focused in this study primarily on the effect of *BRAF*V600E in non-malignant thyroid lesions. A similar analysis was carried out by Hébrant et al. [41], who used primary cultures of human thyrocytes to demonstrate a clear distinction between the gene signature of short-term and long-term EGF/serum-treated cells, but a similar gene expression pattern in long-term EGF/serum-treated cells and PTCs. The altered gene expression in *BRAF*(+) thyroids was foreseeable, as the *BRAF*V600E mutation was previously described as a strong differentiating factor [22]. However, we did not expect a difference of large scale, which was observed in our study.

We are aware that our transgenic mouse model has some limitations as well. The ideal model would consist also of control group with wild-type *BRAF* transgene and would include the intervention to overcome the *BRAF*-induced hypothyroidism and document euthyreosis by TSH serum levels measurements in all animals. In our model, like in previous studies some *BRAF*(+) animals might have increased serum TSH levels [9]. The enlargement of thyroid glands in some of our *BRAF*(+) mice, suggesting hypothyroidism, is in concordance with existing data. To get an indirect insight into TSH effect onto thyroid tissue, we also calculated novel metric called Thyroid Differentiation Score (TDS) for all mouse samples tested in microarray analysis. As expected, PTCs exhibited much lower TDS than asymptomatic thyroids, while the differences between the *BRAF*(+) and (-) normal/benign thyroids were much smaller and non-significant. However, full elucidation of differences between genes driven by *BRAF* V600E only, by TSH only, and genes depending on both factors shall be carried out in well designed *in vitro* and *in vivo* experiments. The further studies of thyroid cancer mouse models deserve a good control of iodine intake as well as adequate monitoring of thyroid function and possible interventions to balance it.

### Genomic PTC data

In a large cohort of almost 400 PTCs in the analysis of the Cancer Genome Atlas Research Network the authors subdivided PTCs into *BRAF*-like and *RAS*-like groups, depending on the exome and RNA sequencing, proteomic profiles, and epigenetic changes [25]. The *RET*(+) PTCs were much closer to *BRAF*-like PTCs than to *RAS*-like ones. The authors demonstrated also that *BRAF*-like PTCs preferentially activate the MAPK pathway, whereas *RAS*-like PTCs signal through MAPK as well as the PI3K pathway. Clinically, the *RAS*-like molecular profile seems to be rare in PTCs, and found almost exclusively in follicular variant PTC [42, 43]. Moreover *RAS* mutations are more frequent in poorly differentiated thyroid carcinomas and anaplastic ones [44], suggesting that *RAS* alterations play a role as a secondary event more than a primary one in PTC development.

Indeed, in our data the hierarchical clustering performed on human samples with the *BRAF*-related 532 transcripts, selected earlier in mouse array, showed that *BRAF*(+) PTCs had a gene expression profile similar to *RET*(+) PTCs, while *RAS*(+) were much closer in their gene signature to PTCs without *BRAF* or *RET* alterations, and even to apparently healthy thyroids. Although this result was not the main focus of our study, it is noteworthy in the context of the molecular PTC background and recent studies. A similar distribution of asymptomatic thyroids and PTCs harboring *BRAF*, *RET*, and *RAS* alterations was observed also by Durand et al. [45], who performed hierarchical clustering exclusively on the published Giordano PTC samples using 51 genes differentially expressed between their *BRAF*/*RET*(+) PTCs and PTC(−). We confirmed that the observed clustering of PTCs with distinct molecular events was not an artifact of the Giordano et al. data, but rather an inherent feature of PTC.

Within the 532-gene signature, we found several pathways that might be of high importance in *BRAF*V600E-induced PTC development. Three out of 23 overrepresented pathways are involved in the endocrine system: These are the “insulin signaling pathway”, which interacts with the MAPK, a flagship *BRAF*-like PTCs pathway (TCGA); the “adipocytokine signaling pathway”, which seems to play a relevant role in PTCs as leptin, its main activator, was reported by Cheng et al. in 2010 to promote migration of PTC cells [46]; and the “PPAR signaling pathway”, which regulates lipid metabolism, adipocyte differentiation, and cell survival.

The *BRAF*-specific character of the 532-gene signature was confirmed by the hierarchical clustering of all mice thyroid samples including PTCs, borderline lesions, benign hyperplastic thyroid lesions, and asymptomatic thyroids, showing perfect separation of *BRAF*(+) from *BRAF*(−) thyroid samples. These human 532 transcripts may consist a plethora of molecules important for PTC, and we used human cohort to further sub-select this group. However, the list may also contain some transcripts related to the design of our study (comparison between *BRAF*V600E-transfected thyroids and thyroids of mice with no transgene), thus, shall be interpreted cautiously. The second step of our analysis–transposition into human arrays and comparison between different tumor groups filters out the potentially confounding transcripts, thus we consider it more robust for further interpretation.

### Signature of *BRAF*-mediated PTC tumorigenesis

The most relevant result of this study is the list of 18 genes obtained from the early *BRAF*(+) PTCs 532-gene signature by selection of genes specific for *BRAF*(+) PTCs in human samples. These 18 genes fulfill stringent selection criteria (FDR<0.05 in comparison between mouse *BRAF*(+) and *BRAF*(-) non malignant thyroids; FDR<0.05 in all 4 comparisons in human material *BRAF*(+) vs *RET*(+), *RAS*(+), PTC(-) and healthy thyroids) which were imposed by us in order to filter signature that will be as specific as possible. Moreover additional comparisons of differences in the expression of these 18 genes performed in other human tumor subtypes [*RET*(+) PTCs and *RAS*(+) PTCs compared to healthy thyroids] indicated that only few of these genes showed the trend observed in *BRAF*(+) tumors. In fact, some of them exhibited the reverse direction of change. Taking into account the fact that all the genes in *BRAF*-specific 18 transcripts signature were selected to significantly differ from *RAS*(+), PTC(-) and *RET*(+) tumors, it confirms the validity of our assumptions that the signature consists of genes truly specific for *BRAF*. However, as presented in hierarchical clustering performed on human samples with the *BRAF*-related 532 transcripts, *RET*(+) PTCs are the closest in the resemblance to *BRAF*(+) tumors, and 13 of our 18 genes are significantly different in *RET*(+) tumors compared to healthy thyroid. However, all of them have fold change much lower than in *BRAF*(+) tumors, except 2, *PLAUR* and *EPHA2*, which were more up-regulated in *RET*(+) PTCs (S4 Table).

Eleven of 18 *BRAF*V600E-specific genes were previously reported as related to thyroid cancer, with 6 documented as associated with *BRAF* mutation (*DCSTAMP*, *MET*, *FN1*, *DIO1*, *EPHA2*, and *ERBB3*; Table 4). The *BRAF*V600E- specific expression profile of 18 genes, observed in our microarray data, was confirmed in a validation analysis performed by us on an independent cohort of PTCs made available by the Cancer Genome Atlas Research Network [25]. The majority of these genes demonstrate the difference between mouse *BRAF*(-) and (+) benign thyroids, [asymptomatic thyroids and hyperplastic thyroid lesions, both *BRAF*(+) as well as *BRAF*(-)]. Moreover all of them showed the direction of change in mouse PTC identical to human PTC, and this trend was also observed in borderline lesions as well.

The majority of the identified genes are known to be altered in thyroid cancer. Among *BRAF*-deregulated genes, there was a thyroid-specific gene, *DIO1*, which was also described as downregulated in PTC by Durand et al. [45]. The role of the *BRAF*V600E mutation in the regulation of thyroid-specific genes is known *inter alia* thanks to the study of Durante et al., who observed much higher reduction of *NIS*, *TG*, and *TPO* expression in *BRAF*(+) PTCs compared with *BRAF*(−) ones [47]. Several of our 18 genes were described as involved in the MAPK pathway, such as *MET*, *ERBB3*, and *PLAUR*, which all encode protein receptors, with the first two being tyrosine kinase-type cell surface receptors. *MET* and *ERBB3* play an important role in proliferation and survival, and they also act through the PI3K/AKT pathway [48, 49]. *DCSTAMP* and *MMD*, with the latter not previously related to thyroid cancer nor to *BRAF*V600E are known to participate in the regulation of the immune response [22]. Similar to results obtained by Giordano et al. [22, 50], *DCSTAMP* is one of the most preferentially expressed genes in our cohort of *BRAF*(+) PTCs. While *MMD* is involved in macrophage activation, the role of *DCSTAMP* is still unknown. Among the *BRAF*V600E-specific differentially expressed 18 genes, there are two involved in the calcification of epithelial cells: *SLC34A2* and *ITPR3*, with the latter not previously related to *BRAF* mutation or to thyroid cancer. *ITPR3* encodes an intracellular Ca^2+^ release channel and is upregulated in actively invading cancer cells and in invasive margin of tumors in colorectal cancer [51] whereas *SLC34A2*, a sodium/phosphate cotransporter, promotes calcium-phosphate calcifications involved in the development of breast cancer [52]. Ca^2+^ is well known as an important regulator of cell proliferation and apoptosis [51].

Two of the 18 *BRAF*V600E-specific genes are believed to be tumor suppressor genes: *PDLIM4* [53] and *IQGAP2* [54], both previously related to thyroid cancer. However, only the latter is downregulated in our cohort of *BRAF*(+) PTCs, while expression of *PDLIM4* is increased.

In addition to *MMD* and *ITPR3*, 5 other genes have not been previously documented as related to thyroid cancer or *BRAF*V600E: *AACS*, *PVRL3*, *RASA1*, *ALDH3B1*, and *LAD1*. The knowledge about the potential role in tumorigenesis of these genes is poor, and further studies are essential. One of them, *ALDH3B1*, an aldehyde dehydrogenase plays a protective role against cellular oxidative stress. Interestingly, its protein is not expressed in certain epithelial tissues, including thyrocytes, with only minor immunoreactivity in some histiocytes located in the connective tissue [55]. Upregulation of *ALDH3B1* in *BRAF*(+) PTCs may constitute a mechanism of cancer cell survival. The remaining 4 genes encode proteins representing distinct functional groups: AACS, protein involved in metabolic processes; PVRL3, adhesion molecule; LAD1, protein involved in epithelial–mesenchymal interaction; RASA1, signal transduction, involved in the control of cell proliferation and differentiation. Although altered expression of most of them has been related to different cancers, their function needs further research.

Our analysis presents novel data, applying homogeneous non-malignant mouse samples for *BRAF*V600E-induced PTCs and human samples. We were able to select 532 genes that we believe represent a gene signature of the pre-cancerous stage of *BRAF*V600E-induced PTC. We have selected a list of 18 genes that are deregulated in a *BRAF*V600E-dependent manner and may be of high importance during early PTC carcinogenesis. This may be useful from a therapeutic point of view. We also found confirmation that a *RAS*(+) molecular subtype of PTC has a gene signature different from that of the *BRAF*(+) and *RET*(+) PTCs gene signatures, whereas *RET*(+) and *BRAF*(+) tumors display close proximity in their gene expression profile. This should be studied in detail, especially to detect additional molecular events that may function in *RAS*(+) PTCs to initiate PTC development.

## Supporting Information

S1 ARRIVE Checklist (PDF)

S1 Fig Nucleotide sequence of the *BRAF* cDNA, including the 600 codon (underlined black line) (A) from the wild-type mouse; (B) from transgenic mouse: overlapping of sequences of wild-type mouse gene and mutated human transgene–differences are indicated by grey arrows and the V600E mutation by a black arrow.
(PDF)

S2 Fig Histopathological evaluation of mice thyroid specimens. A. Apparently asymptomatic thyroid (magnification 200x). B. PTC with mixture of classical and follicular variants (magnification 400x). C. PTC with the structure typical for the cribriform variant (magnification 200x). D. PTC with Hobnail features (magnification 400x). E. Benign hyperplastic lesion (magnification 40x). F. Borderline lesion (magnification 400x).
(PDF)

S3 Fig Histopathological image of aggressiveness of obtained mouse model of *BRAF*V600E-induced PTC. A. Metastatic PTC to the lung with visible inflammation foci (magnification 100x). B. Lung metastasis of PTC with the histopathological details of PTC (magnification 400x). C. Invasion of PTC to the muscle tissue (magnification 200x). D. Infiltration of the surrounding adipose tissue (magnification 100x).
(PDF)

S4 Fig The level of 2HA/BRAF protein expression in mouse thyroids differs between transgenic lines. (A) Western blot detection of the 2HA/BRAF protein by anti-HA antibody. Actin was used as a loading control (asterisk indicates an unspecific bands). As a positive control (+) extracts from cells transiently transfected with pMEV-2HA/BRAF were used. (B) Immunohistochemical detection of the 2HA/BRAF protein by anti-HA antibody. The tg1 thyroid shown is the only one *BRAF*(+) case detected in this line (see S1 Table). DAB (brown) was used as chromogen. Negative controls were performed in parallel by omitting the primary antibody. Magnification 200x.
(TIF)

S5 Fig Venn diagram of 532-gene signature of the *BRAF*V600E-induced PTC early carcinogenesis, representing number of genes differentially expressed between *BRAF*(+) PTCs and *RET*(+) PTCs, *RAS*(+) PTCs, PTCs(−), and healthy thyroids. Eighteen genes were significantly deregulated in all comparisons.
(PDF)

S6 Fig Comparison of thyroid differentiation scores (TDS) between BRAF(+) benign lesions, BRAF(-) benign lesions, borderline lesions and PTCs. Overall significance of differences between the groups p = 0.01 (Kruskal-Wallis test). Pair-wise comparisons assessed by Steel-Dwass post-hoc test are shown on the plot. Only the difference between PTCs and normal/benign BRAF(-) samples was deemed statistically significant, p = 0.0069.
(TIF)

S1 Table Histopathological evaluation of 117 mice. ^a^transgenic–positive for the transgene at the DNA level; non-transgenic–negative for the transgene at the DNA level; *BRAF*(+)–positive for the *BRAF*V600E at the RNA level; *BRAF*(-)–negative for the *BRAF*V600E at the RNA level; Age of mice at the moment of thyroid resection in analyzed groups: 1. Papillary thyroid carcinomas: 6–23 months (the mean age: 11.9 months). 2. Borderline thyroid lesions: 4–19 months (the mean age: 11.6 months). 3. Benign hyperplastic thyroid lesions: 4–12 months (the mean age: 9.5 months). 4. Asymptomatic thyroids: 4–17 months (the mean age: 10.5 months).
(DOC)

S2 Table The 532-gene signature of the *BRAF*V600E-induced PTC early carcinogenesis. (DOC)

S3 Table Comparison of *BRAF*(+) as well as *BRAF*(-) mouse thyroid lesions (including healthy thyroids, hyperplastic lesions, borderline lesions and PTC) to *BRAF*(-) asymptomatic thyroids for 18 genes designated as *BRAF*V600E-dependent. The results are summarized with human analysis of *BRAF*(+) PTCs versus healthy thyroids. a–tested for differences by U Mann-Whitney test (remaining columns were earlier selected for significance, thus are not tested here). b–denotes differences statistically significant at p-value p<0.05 (U Mann-Whitney test).
(DOC)

S4 Table Comparison of human *RET*(+), *RAS*(+) and PTC(-) to healthy thyroids. a- denotes differences statistically significant at FDR<0.05 (U Mann-Whitney test).
(DOC)
