# Supplementary figures and images for: BRAFV600E-Associated Gene Expression Profile: Early Changes in the Transcriptome, Based on a Transgenic Mouse Model of Papillary Thyroid Carcinoma
Source: PLoS One. 2015 Dec 1;10(12):e0143688. doi: 10.1371/journal.pone.0143688 (PMC4666467; doi:10.1371/journal.pone.0143688)

A

600

AGC(A)CAGTGAAATCTCGGTGGAGTGCGGT  
94 97 100 103 106 109 112 115 118 1

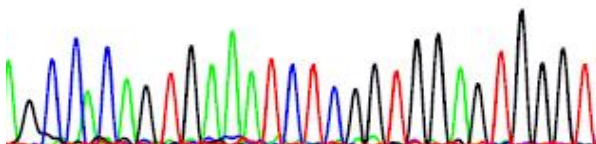

B

600

GCA CAGTGAAATCTCGGTGGAGTGCGGT  
65 69 73 77 81 85 89

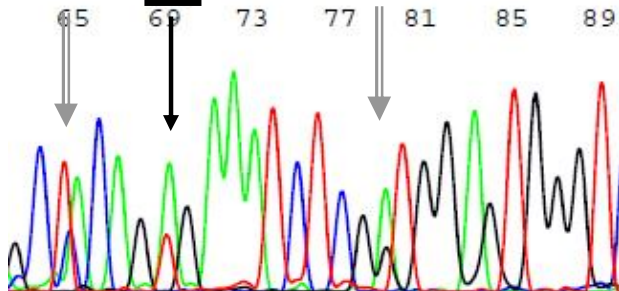

Supplement: S1 Fig — (A) from the wild-type mouse; (B) from transgenic mouse: overlapping of sequences of wild-type mouse gene and mutated human transgene–differences are indicated by grey arrows and the V600E mutation by a black arrow. (PDF) [file pone.0143688.s002.pdf]

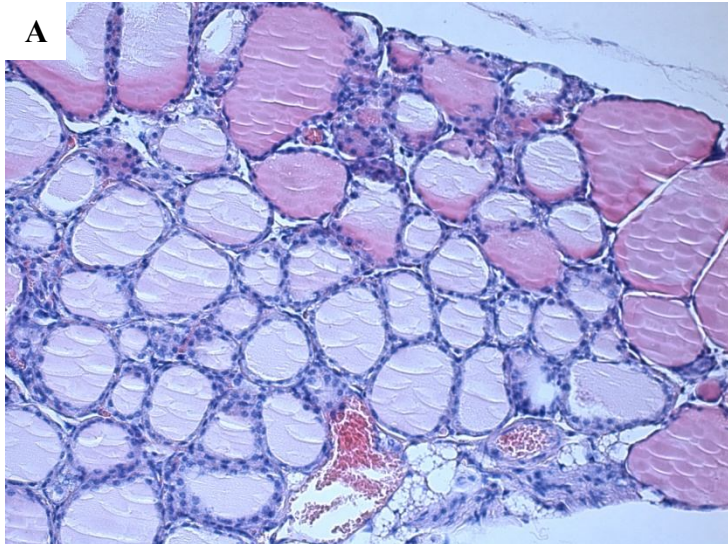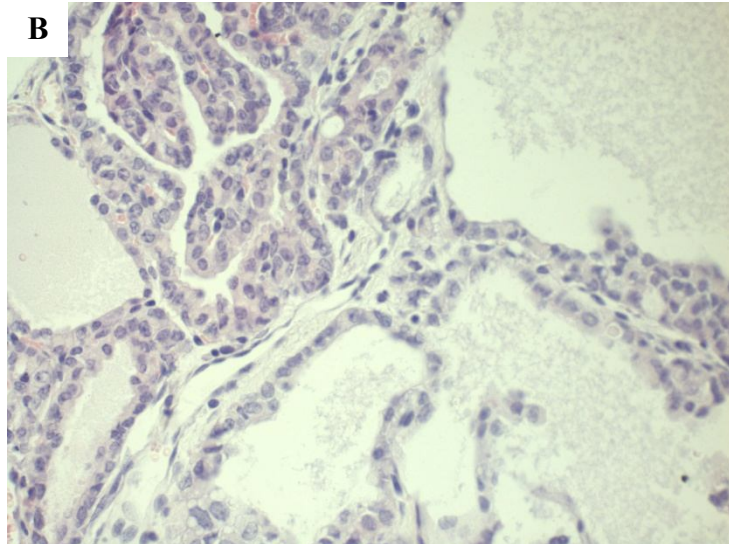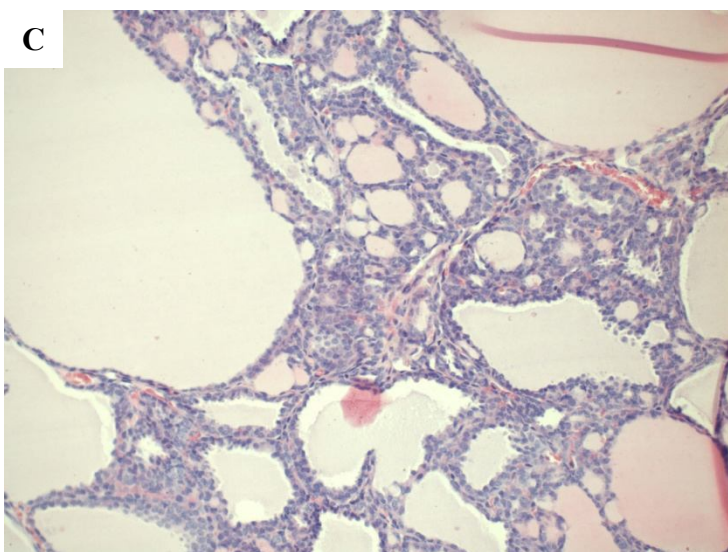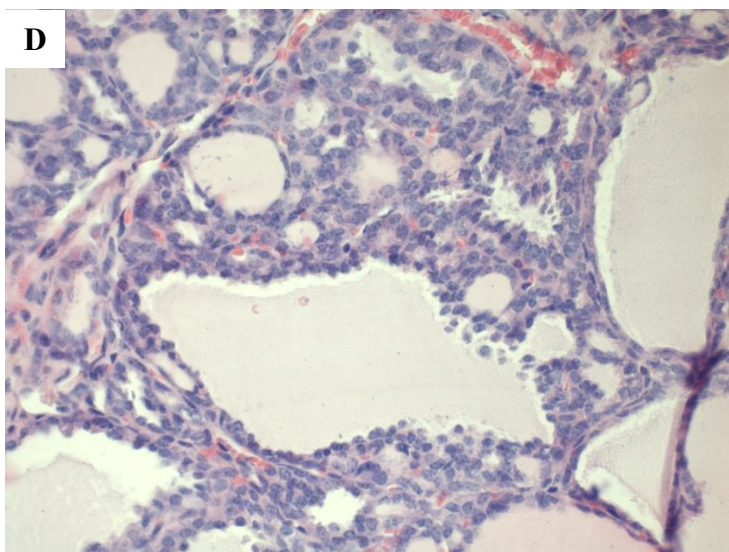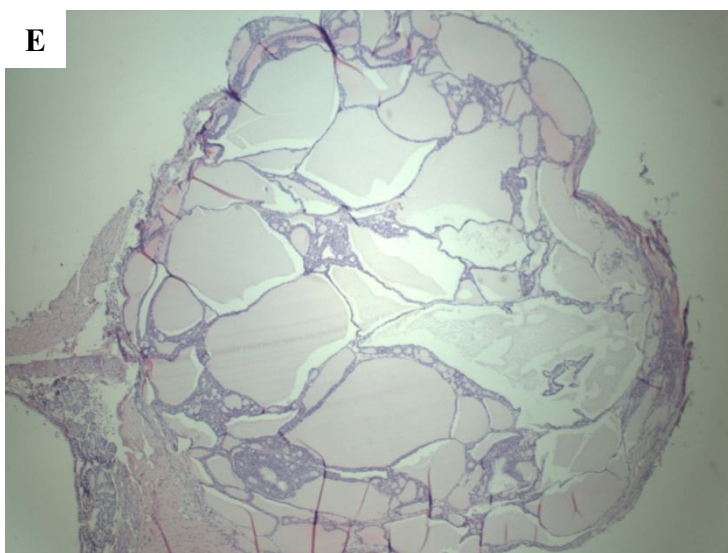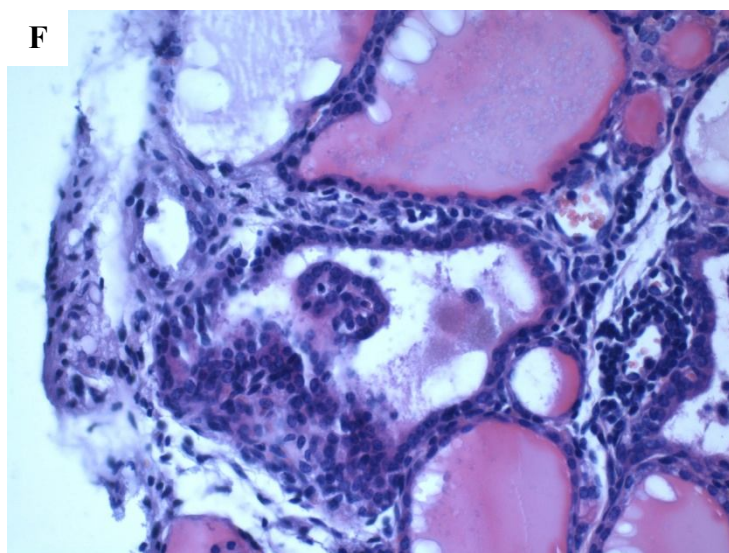

Supplement: S2 Fig — A. Apparently asymptomatic thyroid (magnification 200x). B. PTC with mixture of classical and follicular variants (magnification 400x). C. PTC with the structure typical for the cribriform variant (magnification 200x). D. PTC with Hobnail features (magnification 400x). E. Benign hyperplastic lesion (magnification 40x). F. Borderline lesion (magnification 400x). (PDF) [file pone.0143688.s003.pdf]

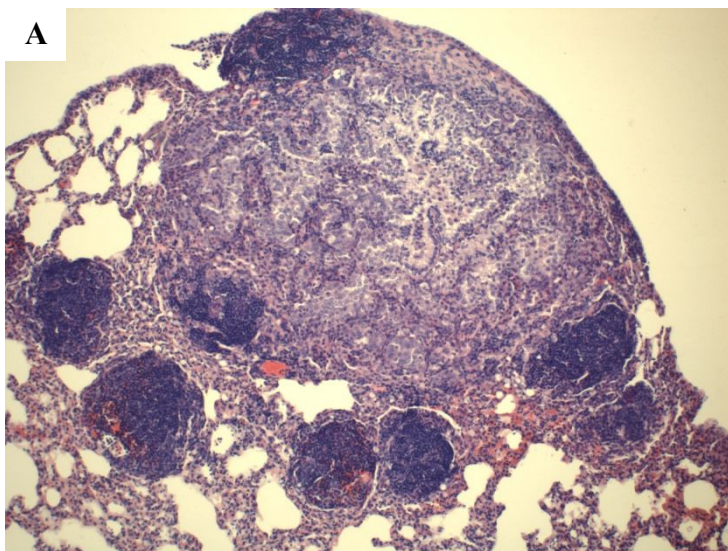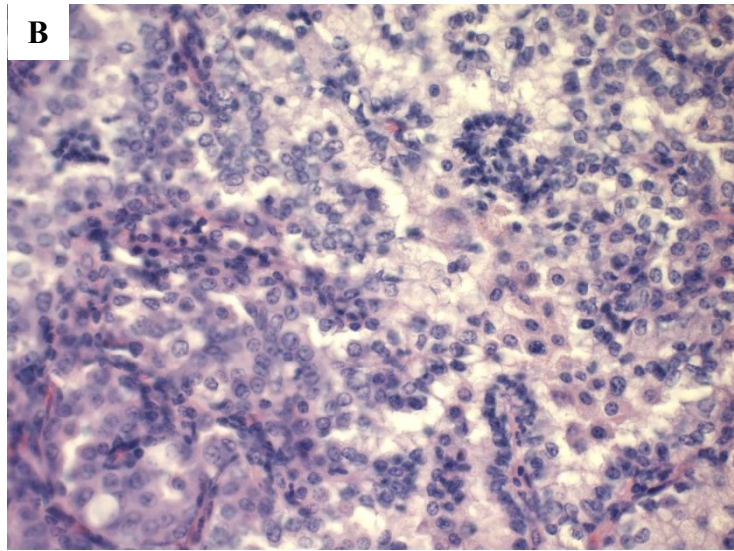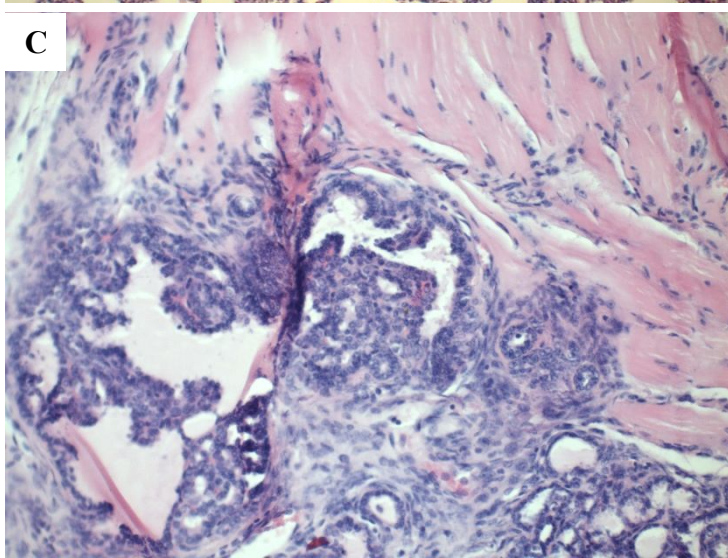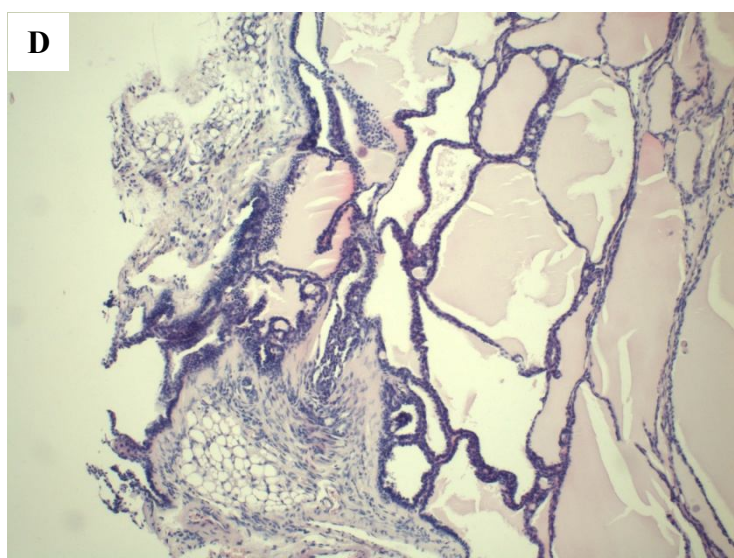

Supplement: S3 Fig — A. Metastatic PTC to the lung with visible inflammation foci (magnification 100x). B. Lung metastasis of PTC with the histopathological details of PTC (magnification 400x). C. Invasion of PTC to the muscle tissue (magnification 200x). D. Infiltration of the surrounding adipose tissue (magnification 100x). (PDF) [file pone.0143688.s004.pdf]

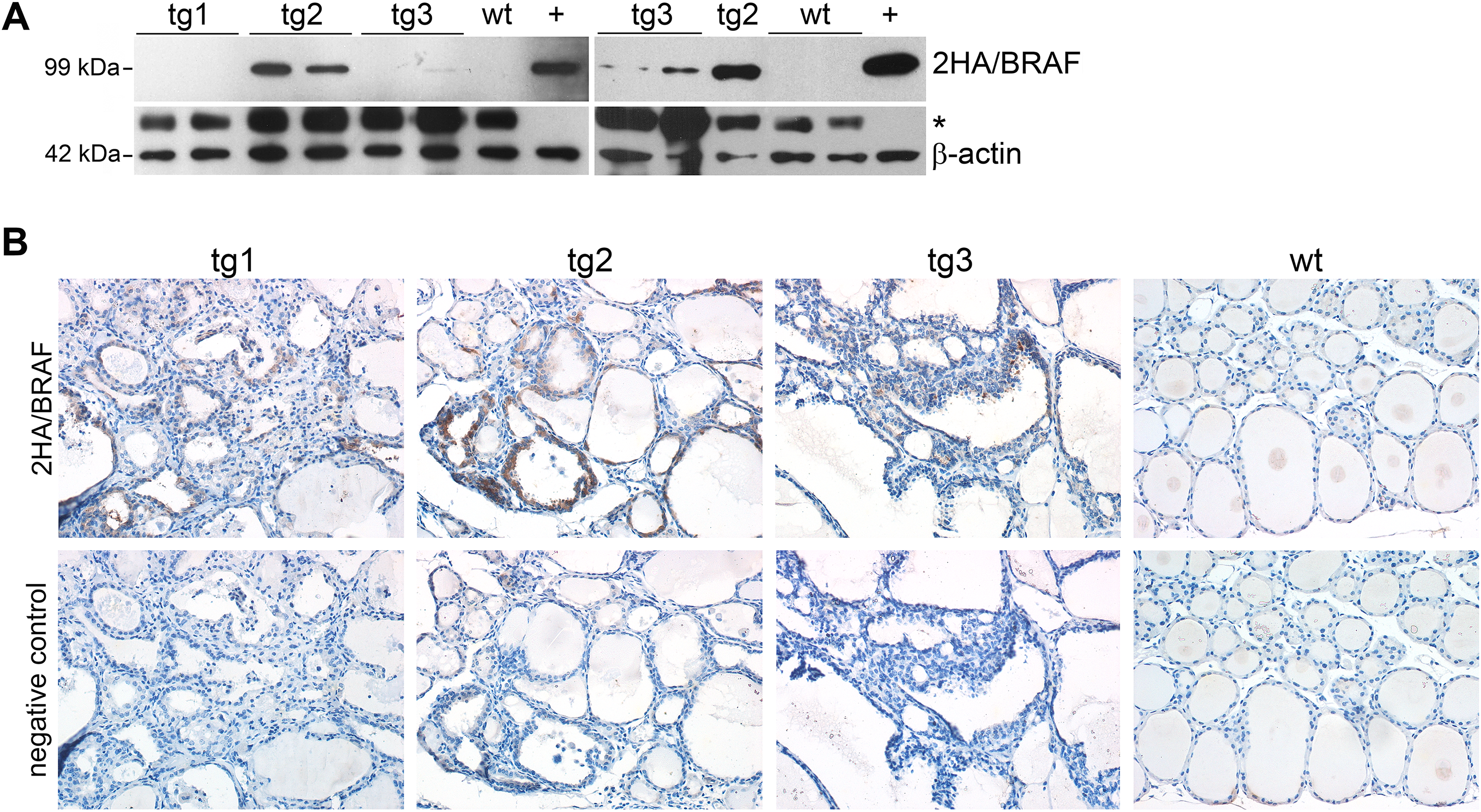

Supplement: S4 Fig — (A) Western blot detection of the 2HA/BRAF protein by anti-HA antibody. Actin was used as a loading control (asterisk indicates an unspecific bands). As a positive control (+) extracts from cells transiently transfected with pMEV-2HA/BRAF were used. (B) Immunohistochemical detection of the 2HA/BRAF protein by anti-HA antibody. The tg1 thyroid shown is the only one BRAF(+) case detected in this line (see S1 Table). DAB (brown) was used as chromogen. Negative controls were performed in parallel by omitting the primary antibody. Magnification 200x. (TIF) [file pone.0143688.s005.TIF]

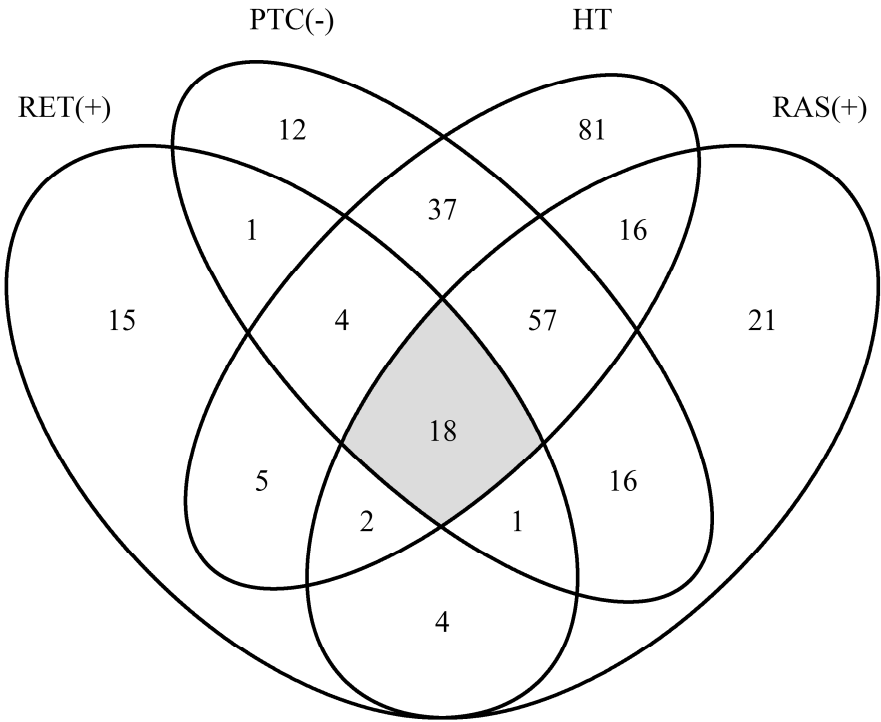

Supplement: S5 Fig — Eighteen genes were significantly deregulated in all comparisons. (PDF) [file pone.0143688.s006.pdf]

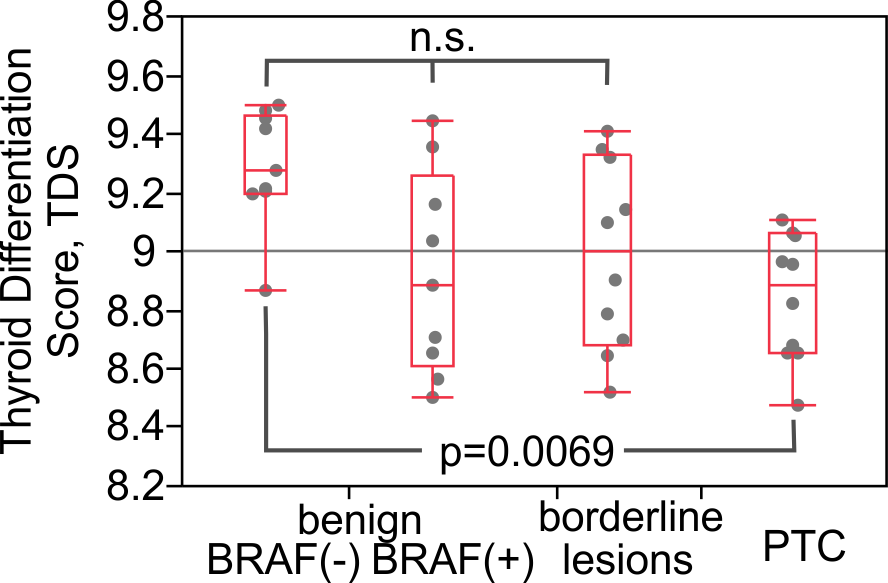

Supplement: S6 Fig — Overall significance of differences between the groups p = 0.01 (Kruskal-Wallis test). Pair-wise comparisons assessed by Steel-Dwass post-hoc test are shown on the plot. Only the difference between PTCs and normal/benign BRAF(-) samples was deemed statistically significant, p = 0.0069. (TIF) [file pone.0143688.s007.tif]
