# Supplementary material for: BRAFV600E-Associated Gene Expression Profile: Early Changes in the Transcriptome, Based on a Transgenic Mouse Model of Papillary Thyroid Carcinoma
Source: PLoS One. 2015 Dec 1;10(12):e0143688. doi: 10.1371/journal.pone.0143688 (PMC4666467; doi:10.1371/journal.pone.0143688)
Supplement: S1 Table — atransgenic–positive for the transgene at the DNA level; non-transgenic–negative for the transgene at the DNA level; BRAF(+)–positive for the BRAFV600E at the RNA level; BRAF(-)–negative for the BRAFV600E at the RNA level; Age of mice at the moment of thyroid resection in analyzed groups: 1. Papillary thyroid carcinomas: 6–23 months (the mean age: 11.9 months). 2. Borderline thyroid lesions: 4–19 months (the mean age: 11.6 months). 3. Benign hyperplastic thyroid lesions: 4–12 months (the mean age: 9.5 months). 4. Asymptomatic thyroids: 4–17 months (the mean age: 10.5 months). (DOC) [file pone.0143688.s008.doc]

**S1 Table Histopathological evaluation of 117 mice**

| **Presence of the transgene at the DNA and RNA level^a^** | **Type of thyroid lesion** | **Number of cases** | | | |
| --- | --- | --- | --- | --- | --- |
|  |  | **tg1 line** | **tg2 line** | **tg3 line** | **Total** |
| Transgenic  *BRAF*(+)  (64 cases) | Papillary thyroid carcinoma | 1/1  100% | 19/25  76% | 19/38  50% | 39/64  61% |
|  | Borderline thyroid lesion | - | 4/25  16% | 10/38  26% | 14/64  22% |
|  | Benign hyperplastic thyroid lesion | - | 1/25  4% | 4/38  11% | 5/64  8% |
|  | Asymptomatic thyroid | - | 1/25  4% | 5/38  13% | 6/64  9% |
| Transgenic  *BRAF*(-)  (25 cases) | Benign hyperplastic thyroid lesion | 1/25  4% | - | - | 1/25  4% |
|  | Asymptomatic thyroid | 24/25  96% | - | - | 24/25  96% |
| Non-transgenic  *BRAF*(+) | - | - | - | - | - |
| Non-transgenic  *BRAF*(-)  (28 cases) | Benign hyperplastic thyroid lesion | - | 2/8  24% | 3/9  33% | 5/28  18% |
|  | Asymptomatic thyroid | 11/11  100% | 6/8  75% | 6/9  67% | 23/28  82% |
| **Total number of cases** |  | **37** | **33** | **47** | **117** |

^a^ transgenic – positive for the transgene at the DNA level; non-transgenic – negative for the transgene at the DNA level; *BRAF*(+) – positive for the *BRAF*V600E at the RNA level; *BRAF*(-) – negative for the *BRAF*V600E at the RNA level;

Age of mice at the moment of thyroid resection in analyzed groups:

1. Papillary thyroid carcinomas: 6-23 months (the mean age: 11.9 months)
2. Borderline thyroid lesions: 4-19 months (the mean age: 11.6 months)
3. Benign hyperplastic thyroid lesions: 4-12 months (the mean age: 9.5 months)
4. Asymptomatic thyroids: 4-17 months (the mean age: 10.5 months)
