# Supplementary material for: BRAFV600E-Associated Gene Expression Profile: Early Changes in the Transcriptome, Based on a Transgenic Mouse Model of Papillary Thyroid Carcinoma
Source: PLoS One. 2015 Dec 1;10(12):e0143688. doi: 10.1371/journal.pone.0143688 (PMC4666467; doi:10.1371/journal.pone.0143688)
Supplement: S2 Table — (DOC) [file pone.0143688.s009.doc]

**S2 Table The 532-gene signature of the *BRAF***V600E-induced PTC early carcinogenesis

| **Gene symbol** | **Geometric mean expression of samples:** | | | | | **Statistical comparison of BRAF(+) samples to:** | | | | | | | | | | | |
| --- | --- | --- | --- | --- | --- | --- | --- | --- | --- | --- | --- | --- | --- | --- | --- | --- | --- |
|  |  |  |  |  |  | **RET(+) samples** | | | **RAS(+) samples** | | | **PTC(-) samples** | | | **healthy thyroid samples** | | |
|  | **BRAF (+)** | **RET**  **(+)** | **RAS**  **(+)** | **PTC**  **(-)** | **HT** | **P-value** | **FDR** | **Fold change** | **P-value** | **FDR** | **Fold change** | **P-value** | **FDR** | **Fold change** | **P-value** | **FDR** | **Fold change** |
| *DCSTAMP* | 850,3 | 34,6 | 4,7 | 5,2 | 13,7 | 2,1E-12 | 1,1E-09 | 24,57 | 4,3E-17 | 1,1E-14 | 181,51 | 2,2E-19 | 1,2E-16 | 162,14 | 6,6E-26 | 1,2E-23 | 61,86 |
| *F3* | 68,9 | 256,4 | 67,3 | 58,0 | 129,0 | 1,3E-07 | 3,5E-05 | 0,27 | 9,4E-01 | 9,7E-01 | 1,02 | 5,6E-01 | 7,5E-01 | 1,19 | 5,3E-04 | 2,0E-03 | 0,53 |
| *MMD* | 82,0 | 190,7 | 437,9 | 200,9 | 151,3 | 1,0E-06 | 1,4E-04 | 0,43 | 1,4E-08 | 4,0E-07 | 0,19 | 7,7E-06 | 1,2E-04 | 0,41 | 9,6E-06 | 5,9E-05 | 0,54 |
| *FAM70A* | 4,9 | 22,6 | 4,2 | 9,4 | 5,4 | 1,0E-06 | 1,4E-04 | 0,22 | 1,5E-01 | 3,6E-01 | 1,18 | 1,0E-02 | 3,8E-02 | 0,53 | 1,4E-01 | 2,4E-01 | 0,91 |
| *SLC34A2* | 3406,4 | 1078,8 | 54,7 | 171,5 | 106,5 | 1,8E-06 | 1,9E-04 | 3,16 | 7,8E-17 | 1,4E-14 | 62,24 | 2,9E-11 | 3,9E-09 | 19,86 | 5,0E-26 | 1,2E-23 | 31,99 |
| *ABCG1* | 9,1 | 16,8 | 23,4 | 10,8 | 8,9 | 6,3E-06 | 5,4E-04 | 0,54 | 6,7E-06 | 9,3E-05 | 0,39 | 2,3E-01 | 4,1E-01 | 0,84 | 8,6E-01 | 8,9E-01 | 1,02 |
| *STC1* | 17,0 | 50,8 | 25,9 | 38,6 | 52,4 | 7,1E-06 | 5,4E-04 | 0,34 | 1,5E-01 | 3,5E-01 | 0,66 | 5,5E-03 | 2,5E-02 | 0,44 | 4,6E-08 | 4,5E-07 | 0,33 |
| *SERPINE1* | 30,3 | 74,8 | 26,5 | 25,3 | 24,0 | 1,3E-05 | 8,8E-04 | 0,41 | 5,2E-01 | 7,6E-01 | 1,14 | 2,9E-01 | 5,0E-01 | 1,20 | 1,6E-01 | 2,7E-01 | 1,26 |
| *ANXA9* | 30,3 | 14,9 | 22,8 | 26,8 | 34,8 | 2,3E-05 | 1,3E-03 | 2,04 | 2,1E-01 | 4,4E-01 | 1,33 | 5,5E-01 | 7,4E-01 | 1,13 | 3,4E-01 | 4,6E-01 | 0,87 |
| *FN1* | 464,2 | 83,2 | 7,4 | 10,4 | 24,6 | 3,2E-05 | 1,7E-03 | 5,58 | 1,0E-10 | 6,0E-09 | 62,78 | 2,3E-10 | 2,5E-08 | 44,84 | 9,2E-15 | 3,3E-13 | 18,87 |
| *LGALS3BP* | 820,5 | 1471,2 | 1042,5 | 887,6 | 753,4 | 4,0E-05 | 1,9E-03 | 0,56 | 1,8E-01 | 4,1E-01 | 0,79 | 6,3E-01 | 8,1E-01 | 0,92 | 5,5E-01 | 6,5E-01 | 1,09 |
| *PLAUR* | 78,5 | 281,9 | 21,4 | 19,7 | 26,9 | 5,0E-05 | 2,2E-03 | 0,28 | 1,3E-03 | 8,7E-03 | 3,67 | 1,2E-04 | 1,1E-03 | 3,98 | 7,9E-05 | 3,5E-04 | 2,92 |
| *CACNA2D2* | 21,5 | 46,4 | 65,9 | 34,9 | 24,4 | 9,0E-05 | 3,7E-03 | 0,46 | 5,0E-06 | 7,4E-05 | 0,33 | 1,4E-02 | 5,1E-02 | 0,61 | 2,9E-01 | 4,2E-01 | 0,88 |
| *EFHD1* | 43,0 | 126,7 | 66,0 | 60,1 | 64,0 | 1,1E-04 | 4,2E-03 | 0,34 | 2,3E-01 | 4,6E-01 | 0,65 | 3,0E-01 | 5,0E-01 | 0,72 | 7,4E-02 | 1,5E-01 | 0,67 |
| *ITPR3* | 933,6 | 548,8 | 452,2 | 430,1 | 416,0 | 1,6E-04 | 5,4E-03 | 1,70 | 9,5E-04 | 6,7E-03 | 2,06 | 2,1E-05 | 2,6E-04 | 2,17 | 1,9E-09 | 2,6E-08 | 2,24 |
| *BCHE* | 6,1 | 32,9 | 33,0 | 5,5 | 5,9 | 1,6E-04 | 5,4E-03 | 0,19 | 1,9E-03 | 1,1E-02 | 0,18 | 7,9E-01 | 8,8E-01 | 1,10 | 9,0E-01 | 9,2E-01 | 1,03 |
| *GRB7* | 38,6 | 22,0 | 19,1 | 20,2 | 17,3 | 2,0E-04 | 5,9E-03 | 1,76 | 1,6E-03 | 1,1E-02 | 2,03 | 2,0E-03 | 1,1E-02 | 1,91 | 9,2E-09 | 1,1E-07 | 2,23 |
| *LTBP1* | 53,8 | 182,8 | 18,2 | 32,6 | 35,0 | 2,0E-04 | 5,9E-03 | 0,29 | 1,3E-02 | 5,1E-02 | 2,96 | 2,2E-01 | 4,1E-01 | 1,65 | 9,1E-02 | 1,7E-01 | 1,54 |
| *PDLIM4* | 503,2 | 302,0 | 30,1 | 39,8 | 47,1 | 2,2E-04 | 6,2E-03 | 1,67 | 1,6E-20 | 8,8E-18 | 16,73 | 1,0E-16 | 2,7E-14 | 12,63 | 1,2E-30 | 6,5E-28 | 10,69 |
| *PTGER4* | 82,1 | 326,3 | 41,7 | 42,9 | 102,7 | 2,4E-04 | 6,4E-03 | 0,25 | 2,6E-01 | 4,9E-01 | 1,97 | 2,0E-01 | 3,7E-01 | 1,91 | 5,1E-01 | 6,2E-01 | 0,80 |
| *HAS2* | 5,1 | 11,0 | 4,7 | 4,6 | 5,1 | 2,6E-04 | 6,4E-03 | 0,46 | 5,6E-01 | 8,0E-01 | 1,10 | 4,8E-01 | 6,8E-01 | 1,10 | 9,0E-01 | 9,2E-01 | 1,01 |
| *MET* | 475,6 | 233,4 | 61,9 | 105,9 | 52,3 | 2,7E-04 | 6,4E-03 | 2,04 | 7,6E-09 | 2,4E-07 | 7,68 | 1,1E-07 | 4,1E-06 | 4,49 | 5,2E-21 | 4,6E-19 | 9,09 |
| *FAM69A* | 16,3 | 36,2 | 14,0 | 23,8 | 38,2 | 3,3E-04 | 7,7E-03 | 0,45 | 6,2E-01 | 8,4E-01 | 1,17 | 1,9E-01 | 3,7E-01 | 0,68 | 4,2E-05 | 2,1E-04 | 0,43 |
| *AACS* | 109,2 | 163,0 | 250,8 | 252,7 | 184,3 | 4,2E-04 | 9,3E-03 | 0,67 | 5,0E-07 | 8,8E-06 | 0,44 | 5,1E-08 | 2,4E-06 | 0,43 | 5,1E-07 | 3,9E-06 | 0,59 |
| *RRAGD* | 172,0 | 55,8 | 122,8 | 424,0 | 275,4 | 6,7E-04 | 1,4E-02 | 3,08 | 4,9E-01 | 7,4E-01 | 1,40 | 2,9E-02 | 8,3E-02 | 0,41 | 8,0E-02 | 1,6E-01 | 0,62 |
| *ACACA* | 118,6 | 155,3 | 215,8 | 169,7 | 115,2 | 7,2E-04 | 1,5E-02 | 0,76 | 2,3E-06 | 3,7E-05 | 0,55 | 9,5E-04 | 5,8E-03 | 0,70 | 6,2E-01 | 7,1E-01 | 1,03 |
| *PHYH* | 304,9 | 176,6 | 375,7 | 507,8 | 491,6 | 7,9E-04 | 1,5E-02 | 1,73 | 3,9E-01 | 6,4E-01 | 0,81 | 9,4E-03 | 3,6E-02 | 0,60 | 4,7E-04 | 1,8E-03 | 0,62 |
| *KIF5C* | 9,2 | 17,5 | 44,6 | 14,6 | 12,5 | 8,2E-04 | 1,5E-02 | 0,52 | 1,8E-05 | 2,2E-04 | 0,21 | 6,5E-02 | 1,5E-01 | 0,63 | 1,0E-02 | 2,7E-02 | 0,73 |
| *RASA1* | 545,3 | 376,4 | 280,0 | 277,9 | 254,9 | 9,5E-04 | 1,7E-02 | 1,45 | 2,2E-05 | 2,5E-04 | 1,95 | 6,5E-07 | 1,8E-05 | 1,96 | 1,5E-12 | 3,3E-11 | 2,14 |
| *CYP2R1* | 4,7 | 4,7 | 4,7 | 4,7 | 4,7 | 9,7E-04 | 1,7E-02 | 1,00 | 2,1E-01 | 4,4E-01 | 1,00 | 1,8E-02 | 5,9E-02 | 1,00 | 1,6E-05 | 8,4E-05 | 1,00 |
| *LAD1* | 54,2 | 31,4 | 21,4 | 19,7 | 16,9 | 1,0E-03 | 1,8E-02 | 1,73 | 1,2E-04 | 1,1E-03 | 2,53 | 8,1E-06 | 1,2E-04 | 2,75 | 5,8E-13 | 1,5E-11 | 3,22 |
| *IQGAP2* | 32,8 | 70,6 | 337,5 | 135,1 | 168,9 | 1,3E-03 | 2,1E-02 | 0,46 | 4,3E-10 | 2,3E-08 | 0,10 | 1,6E-05 | 2,1E-04 | 0,24 | 1,2E-13 | 3,3E-12 | 0,19 |
| *NR4A2* | 68,0 | 297,5 | 37,9 | 40,3 | 89,8 | 1,3E-03 | 2,1E-02 | 0,23 | 3,2E-01 | 5,6E-01 | 1,79 | 3,1E-01 | 5,2E-01 | 1,69 | 4,4E-01 | 5,5E-01 | 0,76 |
| *PTPRD* | 5,0 | 9,0 | 4,3 | 5,9 | 5,5 | 1,3E-03 | 2,1E-02 | 0,56 | 1,9E-01 | 4,2E-01 | 1,16 | 2,4E-01 | 4,2E-01 | 0,85 | 2,1E-01 | 3,2E-01 | 0,92 |
| *ERBB3* | 201,3 | 80,6 | 38,7 | 69,2 | 39,2 | 1,9E-03 | 2,9E-02 | 2,50 | 7,5E-06 | 9,7E-05 | 5,20 | 9,3E-04 | 5,8E-03 | 2,91 | 6,0E-11 | 9,7E-10 | 5,13 |
| *PVRL3* | 37,9 | 84,1 | 120,7 | 162,0 | 117,3 | 2,0E-03 | 3,0E-02 | 0,45 | 6,7E-04 | 4,9E-03 | 0,31 | 4,4E-06 | 8,0E-05 | 0,23 | 4,0E-09 | 5,3E-08 | 0,32 |
| *LPL* | 124,6 | 309,0 | 438,1 | 86,9 | 75,4 | 2,2E-03 | 3,2E-02 | 0,40 | 7,3E-03 | 3,2E-02 | 0,28 | 3,4E-01 | 5,4E-01 | 1,43 | 1,4E-02 | 3,6E-02 | 1,65 |
| *ADAMTS1* | 163,5 | 361,7 | 84,7 | 94,0 | 245,3 | 2,5E-03 | 3,5E-02 | 0,45 | 5,6E-02 | 1,6E-01 | 1,93 | 8,2E-02 | 1,8E-01 | 1,74 | 7,3E-02 | 1,5E-01 | 0,67 |
| *CTGF* | 759,2 | 1577,3 | 326,3 | 702,3 | 1356,0 | 2,6E-03 | 3,6E-02 | 0,48 | 2,9E-02 | 9,4E-02 | 2,33 | 8,2E-01 | 8,9E-01 | 1,08 | 8,6E-03 | 2,3E-02 | 0,56 |
| *ITM2A* | 144,1 | 323,6 | 131,0 | 249,5 | 538,1 | 2,8E-03 | 3,6E-02 | 0,45 | 8,1E-01 | 8,8E-01 | 1,10 | 1,1E-01 | 2,2E-01 | 0,58 | 3,1E-08 | 3,2E-07 | 0,27 |
| *ERBB4* | 6,8 | 15,8 | 7,7 | 34,1 | 21,2 | 2,8E-03 | 3,6E-02 | 0,43 | 6,8E-01 | 8,5E-01 | 0,89 | 2,3E-05 | 2,7E-04 | 0,20 | 1,2E-07 | 1,1E-06 | 0,32 |
| *LZTS1* | 5,0 | 6,7 | 4,6 | 4,9 | 4,8 | 2,9E-03 | 3,6E-02 | 0,74 | 3,5E-01 | 6,0E-01 | 1,08 | 8,0E-01 | 8,8E-01 | 1,02 | 3,3E-01 | 4,6E-01 | 1,05 |
| *GGT5* | 54,3 | 84,5 | 25,2 | 37,6 | 44,7 | 2,9E-03 | 3,6E-02 | 0,64 | 2,2E-04 | 1,8E-03 | 2,15 | 6,3E-02 | 1,5E-01 | 1,44 | 1,2E-01 | 2,2E-01 | 1,22 |
| *EPHA2* | 106,1 | 173,8 | 37,4 | 33,2 | 35,6 | 3,3E-03 | 3,9E-02 | 0,61 | 1,4E-05 | 1,7E-04 | 2,84 | 1,0E-07 | 4,1E-06 | 3,19 | 3,1E-11 | 5,4E-10 | 2,98 |
| *DIO1* | 10,5 | 43,2 | 1230,3 | 320,2 | 601,0 | 3,6E-03 | 4,2E-02 | 0,24 | 2,2E-08 | 5,5E-07 | 0,01 | 2,2E-06 | 4,8E-05 | 0,03 | 3,0E-17 | 1,8E-15 | 0,02 |
| *PCSK5* | 8,7 | 19,6 | 18,7 | 9,9 | 9,0 | 4,0E-03 | 4,6E-02 | 0,44 | 2,4E-02 | 8,3E-02 | 0,47 | 6,2E-01 | 8,0E-01 | 0,88 | 8,3E-01 | 8,8E-01 | 0,97 |
| *PDLIM7* | 28,7 | 41,1 | 24,7 | 19,3 | 17,9 | 4,1E-03 | 4,6E-02 | 0,70 | 3,3E-01 | 5,7E-01 | 1,16 | 4,2E-03 | 2,0E-02 | 1,49 | 8,5E-06 | 5,4E-05 | 1,61 |
| *DDIT4* | 353,2 | 725,2 | 243,3 | 240,0 | 347,4 | 4,2E-03 | 4,6E-02 | 0,49 | 2,4E-01 | 4,7E-01 | 1,45 | 2,0E-01 | 3,7E-01 | 1,47 | 9,4E-01 | 9,5E-01 | 1,02 |
| *DOCK4* | 32,3 | 57,4 | 30,8 | 30,4 | 35,1 | 4,2E-03 | 4,6E-02 | 0,56 | 8,7E-01 | 9,2E-01 | 1,05 | 8,4E-01 | 9,0E-01 | 1,06 | 6,2E-01 | 7,1E-01 | 0,92 |
| *ALDH3B1* | 73,0 | 47,8 | 38,6 | 43,3 | 35,3 | 4,5E-03 | 4,8E-02 | 1,53 | 4,3E-03 | 2,1E-02 | 1,89 | 6,8E-03 | 2,9E-02 | 1,69 | 1,4E-07 | 1,3E-06 | 2,07 |
| *CYR61* | 689,8 | 1401,0 | 494,9 | 873,8 | 1644,9 | 5,0E-03 | 5,2E-02 | 0,49 | 3,7E-01 | 6,2E-01 | 1,39 | 4,7E-01 | 6,8E-01 | 0,79 | 6,0E-05 | 2,9E-04 | 0,42 |
| *FMOD* | 44,0 | 73,0 | 80,7 | 82,2 | 169,5 | 5,4E-03 | 5,4E-02 | 0,60 | 1,4E-02 | 5,3E-02 | 0,55 | 6,7E-03 | 2,9E-02 | 0,54 | 3,1E-14 | 9,7E-13 | 0,26 |
| *MICAL1* | 10,9 | 16,5 | 9,8 | 10,8 | 10,5 | 5,4E-03 | 5,4E-02 | 0,66 | 4,4E-01 | 6,9E-01 | 1,12 | 9,4E-01 | 9,6E-01 | 1,01 | 7,3E-01 | 8,0E-01 | 1,04 |
| *NT5DC2* | 46,7 | 86,7 | 84,4 | 40,7 | 41,7 | 6,6E-03 | 6,5E-02 | 0,54 | 5,3E-02 | 1,5E-01 | 0,55 | 6,0E-01 | 7,8E-01 | 1,15 | 5,3E-01 | 6,4E-01 | 1,12 |
| *HADHB* | 1139,9 | 920,9 | 1549,2 | 1706,4 | 1239,3 | 6,9E-03 | 6,6E-02 | 1,24 | 6,4E-03 | 2,8E-02 | 0,74 | 8,9E-05 | 9,0E-04 | 0,67 | 1,6E-01 | 2,7E-01 | 0,92 |
| *KCNK5* | 71,1 | 116,3 | 77,0 | 84,2 | 41,0 | 7,7E-03 | 7,4E-02 | 0,61 | 7,4E-01 | 8,7E-01 | 0,92 | 4,1E-01 | 6,1E-01 | 0,84 | 2,2E-04 | 8,9E-04 | 1,74 |
| *BNIP3* | 196,3 | 137,5 | 231,3 | 331,8 | 311,0 | 8,4E-03 | 7,8E-02 | 1,43 | 4,0E-01 | 6,6E-01 | 0,85 | 3,0E-03 | 1,5E-02 | 0,59 | 3,7E-05 | 1,8E-04 | 0,63 |
| *JHDM1D* | 56,5 | 78,0 | 70,8 | 60,9 | 62,8 | 8,5E-03 | 7,8E-02 | 0,72 | 2,2E-01 | 4,5E-01 | 0,80 | 5,9E-01 | 7,8E-01 | 0,93 | 3,5E-01 | 4,7E-01 | 0,90 |
| *SLC38A1* | 54,9 | 123,3 | 120,0 | 113,2 | 167,8 | 8,8E-03 | 7,8E-02 | 0,45 | 6,0E-02 | 1,7E-01 | 0,46 | 6,4E-02 | 1,5E-01 | 0,48 | 6,6E-05 | 3,1E-04 | 0,33 |
| *FAM65B* | 11,7 | 22,2 | 7,9 | 7,2 | 11,7 | 8,8E-03 | 7,8E-02 | 0,53 | 2,4E-01 | 4,7E-01 | 1,48 | 9,6E-02 | 2,0E-01 | 1,62 | 1,0E+00 | 1,0E+00 | 1,00 |
| *TSPAN15* | 6,1 | 7,1 | 6,4 | 7,1 | 6,5 | 9,4E-03 | 8,2E-02 | 0,86 | 4,0E-01 | 6,6E-01 | 0,95 | 1,5E-02 | 5,2E-02 | 0,86 | 8,1E-02 | 1,6E-01 | 0,94 |
| *PLP1* | 4,6 | 7,6 | 6,4 | 31,1 | 6,1 | 9,9E-03 | 8,5E-02 | 0,61 | 5,3E-03 | 2,4E-02 | 0,72 | 1,0E-06 | 2,7E-05 | 0,15 | 1,1E-06 | 7,9E-06 | 0,76 |
| *TAOK3* | 208,4 | 263,1 | 310,0 | 301,4 | 240,6 | 1,0E-02 | 8,5E-02 | 0,79 | 2,2E-03 | 1,2E-02 | 0,67 | 4,9E-04 | 3,5E-03 | 0,69 | 3,6E-02 | 8,3E-02 | 0,87 |
| *INSIG1* | 5,4 | 5,8 | 5,4 | 5,4 | 5,4 | 1,0E-02 | 8,6E-02 | 0,93 | 2,9E-01 | 5,3E-01 | 1,00 | 7,5E-02 | 1,7E-01 | 0,99 | 8,5E-01 | 8,9E-01 | 1,00 |
| *NDN* | 121,4 | 237,3 | 142,2 | 187,5 | 191,3 | 1,1E-02 | 9,1E-02 | 0,51 | 7,0E-01 | 8,6E-01 | 0,85 | 2,3E-01 | 4,1E-01 | 0,65 | 3,5E-02 | 8,0E-02 | 0,63 |
| *HBEGF* | 120,4 | 302,0 | 29,6 | 35,6 | 70,8 | 1,1E-02 | 9,2E-02 | 0,40 | 3,3E-03 | 1,7E-02 | 4,07 | 3,2E-03 | 1,6E-02 | 3,38 | 6,1E-02 | 1,3E-01 | 1,70 |
| *APOC1* | 384,4 | 1075,7 | 155,0 | 155,3 | 124,1 | 1,2E-02 | 9,2E-02 | 0,36 | 1,3E-01 | 3,1E-01 | 2,48 | 1,0E-01 | 2,1E-01 | 2,48 | 2,4E-03 | 7,7E-03 | 3,10 |
| *ADRB3* | 4,7 | 4,7 | 4,7 | 4,7 | 4,7 | 1,3E-02 | 9,9E-02 | 0,99 | 6,3E-03 | 2,8E-02 | 0,99 | 4,0E-01 | 6,1E-01 | 1,00 | 2,4E-03 | 7,7E-03 | 1,00 |
| *BACE2* | 62,0 | 90,7 | 88,3 | 59,1 | 49,3 | 1,4E-02 | 1,1E-01 | 0,68 | 1,2E-01 | 2,9E-01 | 0,70 | 8,1E-01 | 8,8E-01 | 1,05 | 8,8E-02 | 1,7E-01 | 1,26 |
| *PTGER3* | 4,7 | 4,9 | 4,7 | 4,7 | 4,6 | 1,5E-02 | 1,1E-01 | 0,95 | 2,6E-01 | 4,9E-01 | 1,00 | 5,6E-03 | 2,5E-02 | 0,99 | 2,1E-05 | 1,1E-04 | 1,01 |
| *DYRK3* | 54,4 | 65,4 | 67,4 | 73,2 | 68,8 | 1,5E-02 | 1,1E-01 | 0,83 | 4,1E-02 | 1,3E-01 | 0,81 | 1,8E-03 | 1,0E-02 | 0,74 | 1,1E-03 | 3,7E-03 | 0,79 |
| *COX6A1* | 3432,7 | 3983,1 | 5960,7 | 5091,0 | 4565,4 | 1,5E-02 | 1,1E-01 | 0,86 | 3,2E-09 | 1,1E-07 | 0,58 | 6,8E-06 | 1,1E-04 | 0,67 | 1,9E-06 | 1,4E-05 | 0,75 |
| *INHBA* | 24,0 | 51,2 | 10,2 | 9,6 | 7,7 | 1,7E-02 | 1,3E-01 | 0,47 | 3,0E-02 | 9,5E-02 | 2,36 | 8,2E-03 | 3,2E-02 | 2,49 | 4,2E-06 | 2,9E-05 | 3,13 |
| *ACTN1* | 494,6 | 657,6 | 261,4 | 182,1 | 251,4 | 1,8E-02 | 1,3E-01 | 0,75 | 1,0E-04 | 8,7E-04 | 1,89 | 2,5E-08 | 1,5E-06 | 2,72 | 1,2E-08 | 1,4E-07 | 1,97 |
| *TMEM45A* | 17,4 | 38,1 | 17,1 | 12,5 | 31,9 | 1,8E-02 | 1,3E-01 | 0,46 | 9,7E-01 | 9,8E-01 | 1,01 | 3,5E-01 | 5,5E-01 | 1,39 | 1,7E-02 | 4,2E-02 | 0,55 |
| *LCN2* | 144,1 | 39,9 | 5,6 | 6,5 | 10,5 | 1,8E-02 | 1,3E-01 | 3,61 | 1,6E-05 | 1,9E-04 | 25,92 | 3,4E-06 | 6,5E-05 | 22,14 | 8,2E-09 | 9,7E-08 | 13,66 |
| *TRIM36* | 6,9 | 17,1 | 24,9 | 26,9 | 7,8 | 1,9E-02 | 1,3E-01 | 0,40 | 9,3E-03 | 3,8E-02 | 0,27 | 7,5E-03 | 3,1E-02 | 0,25 | 5,9E-01 | 6,9E-01 | 0,88 |
| *RBP4* | 4,7 | 5,3 | 5,4 | 5,1 | 4,8 | 1,9E-02 | 1,3E-01 | 0,89 | 3,5E-02 | 1,1E-01 | 0,86 | 1,4E-01 | 2,8E-01 | 0,91 | 4,8E-02 | 1,0E-01 | 0,97 |
| *PLCG2* | 51,3 | 80,6 | 37,3 | 40,3 | 53,6 | 1,9E-02 | 1,3E-01 | 0,64 | 1,9E-01 | 4,2E-01 | 1,37 | 2,7E-01 | 4,7E-01 | 1,27 | 8,0E-01 | 8,5E-01 | 0,96 |
| *GSTA4* | 174,8 | 247,4 | 194,3 | 218,7 | 260,8 | 1,9E-02 | 1,3E-01 | 0,71 | 6,2E-01 | 8,4E-01 | 0,90 | 2,3E-01 | 4,2E-01 | 0,80 | 1,7E-03 | 5,7E-03 | 0,67 |
| *BSPRY* | 187,9 | 127,9 | 342,0 | 359,5 | 264,9 | 1,9E-02 | 1,3E-01 | 1,47 | 9,3E-03 | 3,8E-02 | 0,55 | 8,1E-04 | 5,2E-03 | 0,52 | 9,7E-03 | 2,5E-02 | 0,71 |
| *FGFR2* | 6,8 | 6,2 | 6,7 | 6,4 | 7,8 | 2,0E-02 | 1,3E-01 | 1,11 | 7,1E-01 | 8,6E-01 | 1,02 | 2,7E-01 | 4,7E-01 | 1,06 | 2,9E-02 | 6,9E-02 | 0,88 |
| *NDUFA1* | 1478,5 | 1853,1 | 1769,1 | 2192,9 | 2517,3 | 2,0E-02 | 1,3E-01 | 0,80 | 2,1E-01 | 4,4E-01 | 0,84 | 2,0E-03 | 1,1E-02 | 0,67 | 2,0E-08 | 2,1E-07 | 0,59 |
| *IL1B* | 6,3 | 8,9 | 5,8 | 6,0 | 7,8 | 2,1E-02 | 1,3E-01 | 0,70 | 6,1E-01 | 8,3E-01 | 1,08 | 7,5E-01 | 8,6E-01 | 1,04 | 1,5E-01 | 2,5E-01 | 0,80 |
| *SGMS1* | 318,0 | 221,5 | 132,5 | 109,9 | 183,8 | 2,4E-02 | 1,5E-01 | 1,44 | 1,4E-03 | 9,4E-03 | 2,40 | 2,1E-05 | 2,5E-04 | 2,89 | 1,1E-04 | 5,0E-04 | 1,73 |
| *HS3ST3A1* | 4,8 | 6,0 | 5,1 | 4,9 | 4,5 | 2,4E-02 | 1,5E-01 | 0,80 | 7,7E-02 | 2,0E-01 | 0,93 | 4,7E-01 | 6,8E-01 | 0,98 | 1,6E-03 | 5,3E-03 | 1,06 |
| *GCHFR* | 22,6 | 29,5 | 36,9 | 23,8 | 19,0 | 2,5E-02 | 1,5E-01 | 0,76 | 3,0E-03 | 1,6E-02 | 0,61 | 7,2E-01 | 8,5E-01 | 0,95 | 7,6E-02 | 1,5E-01 | 1,19 |
| *IGF1R* | 47,3 | 35,5 | 28,8 | 33,6 | 39,7 | 2,5E-02 | 1,5E-01 | 1,33 | 4,9E-03 | 2,3E-02 | 1,64 | 3,7E-02 | 9,6E-02 | 1,41 | 1,4E-01 | 2,4E-01 | 1,19 |
| *LIPE* | 4,7 | 4,7 | 4,7 | 4,7 | 4,7 | 2,6E-02 | 1,6E-01 | 0,99 | 7,8E-01 | 8,7E-01 | 1,00 | 7,9E-01 | 8,8E-01 | 1,00 | 3,4E-01 | 4,6E-01 | 1,00 |
| *MCTP1* | 5,0 | 6,1 | 4,8 | 4,9 | 5,3 | 2,7E-02 | 1,6E-01 | 0,82 | 6,7E-01 | 8,5E-01 | 1,03 | 8,6E-01 | 9,1E-01 | 1,01 | 4,1E-01 | 5,2E-01 | 0,93 |
| *IDH3A* | 52,2 | 60,3 | 85,5 | 60,1 | 62,7 | 2,8E-02 | 1,6E-01 | 0,86 | 1,8E-04 | 1,5E-03 | 0,61 | 7,4E-02 | 1,7E-01 | 0,87 | 2,3E-03 | 7,4E-03 | 0,83 |
| *GREB1* | 4,6 | 6,1 | 10,3 | 5,0 | 5,2 | 2,8E-02 | 1,6E-01 | 0,75 | 2,0E-03 | 1,2E-02 | 0,45 | 3,4E-01 | 5,4E-01 | 0,93 | 2,3E-04 | 9,4E-04 | 0,88 |
| *NDRG1* | 335,7 | 432,6 | 226,5 | 380,9 | 524,8 | 2,9E-02 | 1,6E-01 | 0,78 | 2,4E-02 | 8,1E-02 | 1,48 | 3,4E-01 | 5,4E-01 | 0,88 | 1,2E-05 | 7,2E-05 | 0,64 |
| *SDC1* | 106,0 | 171,7 | 163,9 | 111,2 | 68,3 | 2,9E-02 | 1,6E-01 | 0,62 | 1,5E-01 | 3,5E-01 | 0,65 | 8,7E-01 | 9,1E-01 | 0,95 | 1,7E-02 | 4,3E-02 | 1,55 |
| *GLUL* | 2696,7 | 3182,5 | 2714,7 | 4268,9 | 4277,4 | 2,9E-02 | 1,6E-01 | 0,85 | 9,5E-01 | 9,7E-01 | 0,99 | 1,7E-06 | 4,2E-05 | 0,63 | 6,2E-09 | 7,5E-08 | 0,63 |
| *PLCB4* | 59,9 | 154,2 | 431,3 | 121,9 | 158,1 | 3,2E-02 | 1,7E-01 | 0,39 | 4,0E-04 | 3,0E-03 | 0,14 | 1,3E-01 | 2,6E-01 | 0,49 | 1,5E-03 | 5,1E-03 | 0,38 |
| *AEBP1* | 95,7 | 172,5 | 46,7 | 32,3 | 74,8 | 3,2E-02 | 1,7E-01 | 0,56 | 6,1E-02 | 1,7E-01 | 2,05 | 1,4E-03 | 8,5E-03 | 2,96 | 2,7E-01 | 3,9E-01 | 1,28 |
| *EFNB2* | 16,8 | 29,4 | 15,3 | 29,5 | 23,4 | 3,2E-02 | 1,8E-01 | 0,57 | 7,8E-01 | 8,7E-01 | 1,10 | 5,8E-02 | 1,4E-01 | 0,57 | 1,0E-01 | 1,9E-01 | 0,72 |
| *MME* | 4,7 | 6,1 | 4,8 | 4,8 | 4,6 | 3,4E-02 | 1,8E-01 | 0,77 | 2,6E-01 | 4,9E-01 | 0,99 | 2,9E-02 | 8,3E-02 | 0,98 | 1,6E-01 | 2,6E-01 | 1,01 |
| *GPR126* | 5,0 | 7,3 | 4,9 | 5,6 | 5,0 | 3,5E-02 | 1,8E-01 | 0,68 | 9,4E-01 | 9,7E-01 | 1,01 | 1,7E-01 | 3,3E-01 | 0,88 | 8,7E-01 | 9,0E-01 | 0,99 |
| *CP* | 5,6 | 8,9 | 5,5 | 5,8 | 6,6 | 3,6E-02 | 1,9E-01 | 0,62 | 9,6E-01 | 9,7E-01 | 1,01 | 6,7E-01 | 8,4E-01 | 0,95 | 2,2E-01 | 3,3E-01 | 0,84 |
| *CLEC7A* | 28,4 | 43,3 | 12,3 | 16,7 | 18,8 | 3,7E-02 | 1,9E-01 | 0,66 | 2,0E-03 | 1,2E-02 | 2,32 | 3,6E-02 | 9,6E-02 | 1,70 | 2,2E-02 | 5,3E-02 | 1,51 |
| *MYO1E* | 15,3 | 17,7 | 14,6 | 14,8 | 12,5 | 3,7E-02 | 1,9E-01 | 0,87 | 5,6E-01 | 8,0E-01 | 1,05 | 7,4E-01 | 8,6E-01 | 1,03 | 2,4E-04 | 9,8E-04 | 1,22 |
| *FBP1* | 106,1 | 159,0 | 154,3 | 100,8 | 109,1 | 3,7E-02 | 1,9E-01 | 0,67 | 1,9E-01 | 4,1E-01 | 0,69 | 8,7E-01 | 9,1E-01 | 1,05 | 8,6E-01 | 9,0E-01 | 0,97 |
| *ACSL3* | 116,8 | 158,2 | 172,2 | 289,5 | 172,3 | 3,8E-02 | 1,9E-01 | 0,74 | 5,1E-02 | 1,5E-01 | 0,68 | 1,9E-06 | 4,5E-05 | 0,40 | 1,0E-03 | 3,6E-03 | 0,68 |
| *AOC3* | 17,6 | 29,6 | 18,1 | 41,3 | 44,4 | 3,9E-02 | 1,9E-01 | 0,60 | 9,4E-01 | 9,7E-01 | 0,98 | 2,2E-02 | 6,7E-02 | 0,43 | 1,3E-05 | 7,7E-05 | 0,40 |
| *FADS3* | 71,5 | 52,8 | 39,3 | 44,2 | 42,6 | 4,0E-02 | 2,0E-01 | 1,36 | 3,6E-03 | 1,8E-02 | 1,82 | 5,7E-03 | 2,6E-02 | 1,62 | 3,1E-05 | 1,6E-04 | 1,68 |
| *ACPP* | 4,7 | 4,8 | 5,9 | 5,0 | 4,7 | 4,2E-02 | 2,1E-01 | 0,97 | 8,5E-05 | 7,6E-04 | 0,79 | 2,7E-03 | 1,4E-02 | 0,94 | 6,1E-01 | 7,0E-01 | 1,00 |
| *UQCR11* | 907,1 | 1064,0 | 1452,6 | 1155,5 | 1208,4 | 4,2E-02 | 2,1E-01 | 0,85 | 7,8E-05 | 7,1E-04 | 0,62 | 1,9E-02 | 6,0E-02 | 0,79 | 7,2E-05 | 3,3E-04 | 0,75 |
| *FCHSD2* | 87,8 | 112,8 | 82,9 | 89,9 | 103,3 | 4,3E-02 | 2,1E-01 | 0,78 | 7,6E-01 | 8,7E-01 | 1,06 | 8,8E-01 | 9,2E-01 | 0,98 | 1,5E-01 | 2,5E-01 | 0,85 |
| *CALML3* | 4,8 | 5,3 | 4,7 | 4,6 | 4,7 | 4,3E-02 | 2,1E-01 | 0,91 | 5,7E-01 | 8,0E-01 | 1,03 | 2,8E-01 | 4,8E-01 | 1,04 | 3,0E-01 | 4,3E-01 | 1,03 |
| *CRLF1* | 130,1 | 390,0 | 22,4 | 7,9 | 30,2 | 4,4E-02 | 2,1E-01 | 0,33 | 2,4E-02 | 8,3E-02 | 5,80 | 3,1E-05 | 3,5E-04 | 16,45 | 6,7E-04 | 2,4E-03 | 4,31 |
| *JAG1* | 387,1 | 505,4 | 399,1 | 253,5 | 389,9 | 4,4E-02 | 2,1E-01 | 0,77 | 8,5E-01 | 9,0E-01 | 0,97 | 3,2E-02 | 8,8E-02 | 1,53 | 9,4E-01 | 9,5E-01 | 0,99 |
| *HADH* | 233,1 | 193,8 | 323,4 | 314,9 | 337,3 | 4,5E-02 | 2,1E-01 | 1,20 | 1,3E-02 | 5,0E-02 | 0,72 | 1,1E-02 | 4,2E-02 | 0,74 | 5,8E-06 | 3,9E-05 | 0,69 |
| *CHL1* | 5,1 | 7,8 | 4,8 | 7,3 | 5,1 | 4,6E-02 | 2,1E-01 | 0,65 | 6,7E-01 | 8,5E-01 | 1,07 | 1,4E-01 | 2,9E-01 | 0,69 | 9,6E-01 | 9,6E-01 | 1,00 |
| *GLB1L2* | 25,4 | 19,4 | 51,6 | 32,6 | 42,6 | 4,7E-02 | 2,1E-01 | 1,31 | 3,0E-04 | 2,3E-03 | 0,49 | 2,0E-01 | 3,7E-01 | 0,78 | 2,4E-04 | 9,8E-04 | 0,60 |
| *NOV* | 16,3 | 12,1 | 11,5 | 11,9 | 22,7 | 4,7E-02 | 2,1E-01 | 1,35 | 7,8E-02 | 2,1E-01 | 1,42 | 7,1E-02 | 1,7E-01 | 1,36 | 2,8E-02 | 6,7E-02 | 0,72 |
| *S100A14* | 352,2 | 249,2 | 388,8 | 327,4 | 242,9 | 4,8E-02 | 2,1E-01 | 1,41 | 6,9E-01 | 8,5E-01 | 0,91 | 7,5E-01 | 8,6E-01 | 1,08 | 1,5E-02 | 3,8E-02 | 1,45 |
| *BST1* | 4,8 | 5,2 | 4,7 | 4,8 | 4,7 | 4,8E-02 | 2,1E-01 | 0,91 | 7,9E-01 | 8,7E-01 | 1,01 | 9,8E-01 | 9,8E-01 | 1,00 | 6,6E-01 | 7,4E-01 | 1,01 |
| *PON1* | 4,7 | 4,7 | 4,7 | 4,7 | 4,7 | 4,9E-02 | 2,1E-01 | 0,99 | 8,7E-01 | 9,2E-01 | 1,00 | 5,2E-02 | 1,3E-01 | 1,00 | 2,2E-01 | 3,3E-01 | 1,00 |
| *MIF* | 2021,7 | 2395,8 | 2189,1 | 2016,6 | 2108,4 | 4,9E-02 | 2,1E-01 | 0,84 | 5,6E-01 | 8,0E-01 | 0,92 | 9,8E-01 | 9,8E-01 | 1,00 | 6,0E-01 | 6,9E-01 | 0,96 |
| *IL10RA* | 19,0 | 36,9 | 8,5 | 12,5 | 17,8 | 4,9E-02 | 2,1E-01 | 0,51 | 8,4E-02 | 2,2E-01 | 2,23 | 3,1E-01 | 5,1E-01 | 1,52 | 8,5E-01 | 8,9E-01 | 1,06 |
| *PHLDA2* | 191,1 | 276,5 | 58,3 | 64,5 | 41,1 | 5,1E-02 | 2,2E-01 | 0,69 | 6,9E-06 | 9,3E-05 | 3,28 | 7,6E-05 | 7,9E-04 | 2,96 | 8,6E-13 | 2,0E-11 | 4,65 |
| *FSCN1* | 8,8 | 11,7 | 8,2 | 8,3 | 9,1 | 5,1E-02 | 2,2E-01 | 0,75 | 6,7E-01 | 8,5E-01 | 1,07 | 6,9E-01 | 8,5E-01 | 1,06 | 7,9E-01 | 8,5E-01 | 0,97 |
| *TFRC* | 280,3 | 351,2 | 268,4 | 288,3 | 343,8 | 5,5E-02 | 2,3E-01 | 0,80 | 8,0E-01 | 8,8E-01 | 1,04 | 8,6E-01 | 9,1E-01 | 0,97 | 6,5E-02 | 1,3E-01 | 0,82 |
| *TRPV6* | 10,6 | 9,0 | 11,5 | 9,6 | 8,6 | 5,6E-02 | 2,3E-01 | 1,18 | 5,7E-01 | 8,0E-01 | 0,93 | 3,5E-01 | 5,4E-01 | 1,11 | 5,0E-03 | 1,4E-02 | 1,24 |
| *FXYD3* | 8,0 | 13,5 | 5,3 | 5,3 | 5,4 | 5,7E-02 | 2,4E-01 | 0,59 | 1,4E-01 | 3,3E-01 | 1,50 | 8,3E-02 | 1,8E-01 | 1,50 | 1,4E-02 | 3,5E-02 | 1,47 |
| *GPR176* | 4,7 | 4,9 | 5,0 | 4,7 | 4,7 | 5,7E-02 | 2,4E-01 | 0,95 | 7,5E-03 | 3,2E-02 | 0,93 | 4,1E-04 | 3,0E-03 | 0,99 | 9,3E-01 | 9,3E-01 | 1,00 |
| *UQCRQ* | 1419,8 | 1599,7 | 2072,9 | 1786,7 | 1678,5 | 5,9E-02 | 2,5E-01 | 0,89 | 1,6E-04 | 1,3E-03 | 0,68 | 6,4E-03 | 2,8E-02 | 0,79 | 5,2E-03 | 1,4E-02 | 0,85 |
| *ARL6IP1* | 1265,6 | 1449,5 | 1360,6 | 1474,8 | 1695,2 | 6,2E-02 | 2,5E-01 | 0,87 | 5,3E-01 | 7,8E-01 | 0,93 | 9,6E-02 | 2,0E-01 | 0,86 | 1,4E-05 | 7,9E-05 | 0,75 |
| *PTPN13* | 176,7 | 241,6 | 165,7 | 216,6 | 210,4 | 6,3E-02 | 2,6E-01 | 0,73 | 7,4E-01 | 8,7E-01 | 1,07 | 3,5E-01 | 5,4E-01 | 0,82 | 1,7E-01 | 2,8E-01 | 0,84 |
| *SDHB* | 254,7 | 210,8 | 324,2 | 305,1 | 327,4 | 6,5E-02 | 2,6E-01 | 1,21 | 6,6E-02 | 1,8E-01 | 0,79 | 1,4E-01 | 2,7E-01 | 0,83 | 2,9E-03 | 9,1E-03 | 0,78 |
| *PPAP2A* | 447,8 | 605,9 | 694,7 | 712,7 | 794,0 | 6,5E-02 | 2,6E-01 | 0,74 | 6,3E-02 | 1,7E-01 | 0,64 | 1,9E-02 | 6,0E-02 | 0,63 | 2,8E-05 | 1,4E-04 | 0,56 |
| *FAM3C* | 1599,3 | 1311,0 | 1536,0 | 1262,5 | 1225,1 | 6,5E-02 | 2,6E-01 | 1,22 | 8,1E-01 | 8,8E-01 | 1,04 | 8,9E-02 | 1,9E-01 | 1,27 | 5,2E-03 | 1,4E-02 | 1,31 |
| *SLC7A11* | 21,0 | 35,7 | 58,5 | 17,6 | 23,9 | 6,7E-02 | 2,6E-01 | 0,59 | 1,9E-02 | 6,7E-02 | 0,36 | 6,5E-01 | 8,3E-01 | 1,19 | 6,1E-01 | 7,0E-01 | 0,88 |
| *EPHA5* | 4,7 | 5,0 | 4,7 | 4,7 | 4,7 | 6,7E-02 | 2,6E-01 | 0,94 | 9,6E-01 | 9,7E-01 | 1,00 | 8,6E-01 | 9,1E-01 | 1,00 | 3,9E-01 | 5,1E-01 | 1,01 |
| *CYTH4* | 8,5 | 10,4 | 7,4 | 7,7 | 8,1 | 6,8E-02 | 2,6E-01 | 0,81 | 2,1E-01 | 4,4E-01 | 1,15 | 2,8E-01 | 4,8E-01 | 1,11 | 5,9E-01 | 6,9E-01 | 1,05 |
| *ITGAX* | 5,1 | 5,8 | 4,8 | 4,9 | 5,0 | 6,9E-02 | 2,6E-01 | 0,87 | 2,9E-01 | 5,2E-01 | 1,07 | 5,0E-01 | 6,9E-01 | 1,04 | 7,1E-01 | 7,9E-01 | 1,02 |
| *CFP* | 4,7 | 4,9 | 4,7 | 4,8 | 4,9 | 6,9E-02 | 2,6E-01 | 0,97 | 7,4E-01 | 8,7E-01 | 1,00 | 3,4E-01 | 5,4E-01 | 0,99 | 1,4E-01 | 2,5E-01 | 0,96 |
| *AGRN* | 643,4 | 791,8 | 550,3 | 440,7 | 386,4 | 6,9E-02 | 2,6E-01 | 0,81 | 2,7E-01 | 5,1E-01 | 1,17 | 7,4E-03 | 3,1E-02 | 1,46 | 7,5E-05 | 3,4E-04 | 1,67 |
| *ORM1* | 4,7 | 4,8 | 4,7 | 4,8 | 4,7 | 7,1E-02 | 2,7E-01 | 0,97 | 8,4E-01 | 9,0E-01 | 1,00 | 2,6E-02 | 7,7E-02 | 0,97 | 1,2E-01 | 2,2E-01 | 1,00 |
| *MICAL2* | 297,5 | 411,0 | 52,6 | 52,2 | 108,2 | 8,1E-02 | 3,0E-01 | 0,72 | 5,7E-08 | 1,2E-06 | 5,65 | 1,7E-07 | 6,2E-06 | 5,70 | 4,4E-09 | 5,7E-08 | 2,75 |
| *PFKFB1* | 4,7 | 4,7 | 4,7 | 4,7 | 4,7 | 8,6E-02 | 3,2E-01 | 1,00 | 2,0E-01 | 4,4E-01 | 1,00 | 9,6E-03 | 3,7E-02 | 1,00 | 2,6E-01 | 3,9E-01 | 0,99 |
| *NTRK3* | 4,7 | 4,7 | 5,0 | 6,2 | 4,8 | 8,8E-02 | 3,2E-01 | 0,98 | 2,3E-02 | 8,1E-02 | 0,93 | 1,6E-04 | 1,4E-03 | 0,75 | 7,0E-05 | 3,3E-04 | 0,97 |
| *CACNA2D1* | 4,7 | 4,8 | 4,7 | 4,8 | 4,7 | 8,9E-02 | 3,2E-01 | 0,98 | 7,5E-02 | 2,0E-01 | 0,99 | 1,7E-02 | 5,9E-02 | 0,97 | 2,9E-01 | 4,2E-01 | 1,00 |
| *SFTPB* | 1616,6 | 735,5 | 7,5 | 25,3 | 52,6 | 8,9E-02 | 3,2E-01 | 2,20 | 2,3E-13 | 2,4E-11 | 214,79 | 1,5E-09 | 1,3E-07 | 63,81 | 2,1E-13 | 5,6E-12 | 30,75 |
| *GFRA1* | 4,7 | 4,7 | 4,7 | 4,7 | 4,7 | 8,9E-02 | 3,2E-01 | 1,00 | 2,1E-01 | 4,4E-01 | 1,00 | 1,8E-02 | 5,9E-02 | 1,00 | 1,6E-05 | 8,4E-05 | 1,00 |
| *SCGB1A1* | 4,7 | 4,7 | 4,7 | 4,7 | 4,7 | 8,9E-02 | 3,2E-01 | 1,00 | 2,1E-01 | 4,4E-01 | 1,00 | 1,8E-02 | 5,9E-02 | 1,00 | 1,6E-05 | 8,4E-05 | 1,00 |
| *EYA1* | 4,7 | 4,9 | 4,7 | 4,7 | 4,6 | 8,9E-02 | 3,2E-01 | 0,96 | 2,1E-01 | 4,4E-01 | 1,00 | 1,8E-02 | 5,9E-02 | 0,99 | 1,6E-05 | 8,4E-05 | 1,01 |
| *KCNIP2* | 4,7 | 4,7 | 4,7 | 4,7 | 4,7 | 9,0E-02 | 3,2E-01 | 1,00 | 1,8E-02 | 6,6E-02 | 0,99 | 4,7E-02 | 1,2E-01 | 0,98 | 5,4E-05 | 2,6E-04 | 1,00 |
| *DFNA5* | 126,4 | 172,8 | 116,5 | 90,6 | 144,1 | 9,0E-02 | 3,2E-01 | 0,73 | 7,6E-01 | 8,7E-01 | 1,09 | 2,1E-01 | 3,9E-01 | 1,40 | 3,9E-01 | 5,1E-01 | 0,88 |
| *SLC16A10* | 4,8 | 5,6 | 6,1 | 6,0 | 4,9 | 9,0E-02 | 3,2E-01 | 0,86 | 5,6E-02 | 1,6E-01 | 0,78 | 6,8E-02 | 1,6E-01 | 0,80 | 5,1E-01 | 6,2E-01 | 0,97 |
| *SLC2A4* | 4,7 | 4,7 | 4,7 | 4,7 | 4,7 | 9,1E-02 | 3,2E-01 | 0,99 | 2,1E-01 | 4,4E-01 | 1,00 | 1,8E-02 | 5,9E-02 | 1,00 | 6,9E-02 | 1,4E-01 | 1,00 |
| *EPHX2* | 43,2 | 35,8 | 65,7 | 46,0 | 58,8 | 9,2E-02 | 3,2E-01 | 1,21 | 2,6E-03 | 1,4E-02 | 0,66 | 7,3E-01 | 8,6E-01 | 0,94 | 1,0E-03 | 3,5E-03 | 0,73 |
| *DMBT1* | 5,1 | 6,0 | 4,9 | 5,3 | 5,0 | 9,2E-02 | 3,2E-01 | 0,84 | 6,7E-01 | 8,5E-01 | 1,03 | 4,7E-01 | 6,8E-01 | 0,95 | 6,0E-01 | 6,9E-01 | 1,02 |
| *SLC4A7* | 13,1 | 16,4 | 8,2 | 7,8 | 10,3 | 9,4E-02 | 3,2E-01 | 0,80 | 3,7E-03 | 1,8E-02 | 1,60 | 8,3E-04 | 5,3E-03 | 1,68 | 4,3E-02 | 9,5E-02 | 1,28 |
| *ADCY3* | 20,1 | 23,2 | 20,4 | 18,1 | 21,8 | 9,6E-02 | 3,3E-01 | 0,86 | 8,7E-01 | 9,2E-01 | 0,98 | 2,2E-01 | 4,1E-01 | 1,11 | 2,4E-01 | 3,6E-01 | 0,92 |
| *TRAM2* | 47,1 | 68,5 | 45,0 | 49,3 | 115,8 | 9,7E-02 | 3,3E-01 | 0,69 | 9,0E-01 | 9,4E-01 | 1,04 | 8,7E-01 | 9,1E-01 | 0,95 | 1,3E-05 | 7,8E-05 | 0,41 |
| *IL18* | 13,6 | 21,0 | 7,0 | 9,0 | 9,5 | 9,9E-02 | 3,3E-01 | 0,65 | 4,2E-02 | 1,3E-01 | 1,93 | 1,5E-01 | 3,0E-01 | 1,51 | 8,8E-02 | 1,7E-01 | 1,42 |
| *ADRA1A* | 5,0 | 4,7 | 4,7 | 4,7 | 4,7 | 9,9E-02 | 3,3E-01 | 1,07 | 2,4E-01 | 4,7E-01 | 1,07 | 1,8E-01 | 3,5E-01 | 1,07 | 5,1E-02 | 1,1E-01 | 1,07 |
| *AR* | 4,7 | 4,9 | 5,1 | 6,8 | 5,1 | 9,9E-02 | 3,3E-01 | 0,95 | 2,8E-02 | 9,1E-02 | 0,91 | 2,0E-04 | 1,6E-03 | 0,69 | 5,5E-04 | 2,0E-03 | 0,93 |
| *TRAF1* | 7,3 | 8,4 | 6,8 | 6,9 | 7,2 | 1,0E-01 | 3,3E-01 | 0,87 | 4,9E-01 | 7,4E-01 | 1,07 | 4,9E-01 | 6,9E-01 | 1,06 | 8,6E-01 | 8,9E-01 | 1,02 |
| *MMP12* | 6,2 | 9,4 | 4,7 | 4,8 | 5,1 | 1,0E-01 | 3,3E-01 | 0,66 | 3,1E-01 | 5,5E-01 | 1,31 | 2,8E-01 | 4,8E-01 | 1,29 | 2,5E-01 | 3,6E-01 | 1,21 |
| *KCNIP1* | 4,7 | 4,7 | 4,7 | 4,7 | 4,7 | 1,0E-01 | 3,3E-01 | 1,00 | 2,1E-01 | 4,4E-01 | 1,00 | 3,0E-02 | 8,5E-02 | 1,00 | 3,9E-02 | 8,7E-02 | 1,00 |
| *STXBP1* | 109,5 | 167,7 | 267,9 | 386,0 | 159,9 | 1,0E-01 | 3,3E-01 | 0,65 | 1,3E-02 | 5,0E-02 | 0,41 | 8,6E-05 | 8,8E-04 | 0,28 | 8,2E-02 | 1,6E-01 | 0,68 |
| *ALDH1A2* | 5,3 | 4,7 | 5,2 | 5,2 | 4,8 | 1,0E-01 | 3,3E-01 | 1,14 | 8,7E-01 | 9,2E-01 | 1,02 | 8,6E-01 | 9,1E-01 | 1,02 | 1,7E-01 | 2,8E-01 | 1,10 |
| *HYAL1* | 5,0 | 5,2 | 5,4 | 5,2 | 6,0 | 1,0E-01 | 3,3E-01 | 0,96 | 7,8E-03 | 3,3E-02 | 0,91 | 1,8E-02 | 5,9E-02 | 0,95 | 9,1E-04 | 3,2E-03 | 0,83 |
| *B4GALT6* | 7,2 | 10,4 | 7,5 | 23,2 | 6,6 | 1,1E-01 | 3,4E-01 | 0,69 | 9,0E-01 | 9,4E-01 | 0,96 | 2,0E-04 | 1,6E-03 | 0,31 | 6,0E-01 | 6,9E-01 | 1,09 |
| *CTSC* | 2034,9 | 2568,8 | 263,7 | 576,2 | 526,5 | 1,1E-01 | 3,5E-01 | 0,79 | 2,5E-12 | 1,9E-10 | 7,72 | 2,2E-06 | 4,8E-05 | 3,53 | 3,8E-14 | 1,1E-12 | 3,86 |
| *PTPRN* | 5,0 | 4,9 | 5,1 | 4,9 | 4,8 | 1,1E-01 | 3,6E-01 | 1,02 | 2,9E-01 | 5,3E-01 | 0,98 | 7,6E-01 | 8,6E-01 | 1,00 | 1,8E-02 | 4,4E-02 | 1,02 |
| *SEMA3C* | 12,7 | 23,1 | 5,6 | 6,2 | 7,6 | 1,2E-01 | 3,6E-01 | 0,55 | 1,0E-01 | 2,6E-01 | 2,25 | 9,5E-02 | 2,0E-01 | 2,04 | 7,6E-02 | 1,5E-01 | 1,67 |
| *CYP2E1* | 6,5 | 5,6 | 5,4 | 5,6 | 5,1 | 1,2E-01 | 3,6E-01 | 1,16 | 1,7E-01 | 4,0E-01 | 1,20 | 1,9E-01 | 3,7E-01 | 1,17 | 2,8E-03 | 8,7E-03 | 1,27 |
| *FSTL4* | 4,7 | 4,7 | 4,7 | 5,1 | 4,7 | 1,2E-01 | 3,6E-01 | 0,99 | 5,0E-01 | 7,5E-01 | 1,00 | 3,4E-02 | 9,2E-02 | 0,92 | 2,4E-01 | 3,6E-01 | 1,00 |
| *DCHS1* | 19,4 | 24,2 | 32,6 | 26,3 | 23,3 | 1,2E-01 | 3,6E-01 | 0,80 | 2,2E-02 | 7,7E-02 | 0,60 | 9,5E-02 | 2,0E-01 | 0,74 | 1,8E-01 | 2,9E-01 | 0,83 |
| *MBOAT2* | 227,5 | 174,5 | 87,7 | 90,1 | 111,9 | 1,2E-01 | 3,6E-01 | 1,30 | 5,5E-04 | 4,1E-03 | 2,59 | 1,5E-04 | 1,4E-03 | 2,52 | 1,8E-05 | 9,5E-05 | 2,03 |
| *ACAA2* | 24,1 | 21,0 | 24,3 | 20,0 | 19,5 | 1,2E-01 | 3,6E-01 | 1,15 | 9,6E-01 | 9,7E-01 | 0,99 | 7,6E-02 | 1,7E-01 | 1,20 | 5,4E-03 | 1,5E-02 | 1,24 |
| *DUSP6* | 885,4 | 1150,0 | 154,2 | 205,7 | 149,0 | 1,2E-01 | 3,7E-01 | 0,77 | 2,2E-09 | 9,6E-08 | 5,74 | 5,1E-07 | 1,5E-05 | 4,30 | 9,0E-16 | 4,4E-14 | 5,94 |
| *SLCO4C1* | 5,9 | 4,7 | 9,9 | 13,5 | 4,9 | 1,3E-01 | 3,8E-01 | 1,25 | 1,4E-01 | 3,3E-01 | 0,60 | 3,5E-03 | 1,7E-02 | 0,44 | 1,4E-01 | 2,5E-01 | 1,20 |
| *TGFBI* | 583,5 | 828,2 | 117,0 | 184,5 | 260,7 | 1,3E-01 | 3,8E-01 | 0,70 | 5,5E-05 | 5,4E-04 | 4,99 | 5,8E-04 | 4,0E-03 | 3,16 | 1,6E-04 | 6,8E-04 | 2,24 |
| *MRPL42* | 167,7 | 140,9 | 182,3 | 206,3 | 200,0 | 1,3E-01 | 3,8E-01 | 1,19 | 5,7E-01 | 8,0E-01 | 0,92 | 1,3E-01 | 2,7E-01 | 0,81 | 6,6E-02 | 1,4E-01 | 0,84 |
| *FAM46C* | 49,3 | 74,0 | 39,1 | 46,9 | 89,3 | 1,3E-01 | 3,9E-01 | 0,67 | 5,4E-01 | 7,9E-01 | 1,26 | 8,8E-01 | 9,2E-01 | 1,05 | 7,1E-03 | 1,9E-02 | 0,55 |
| *PLAG1* | 79,0 | 57,1 | 39,8 | 32,7 | 24,1 | 1,3E-01 | 3,9E-01 | 1,38 | 3,3E-02 | 1,1E-01 | 1,99 | 9,2E-03 | 3,6E-02 | 2,42 | 1,4E-08 | 1,5E-07 | 3,28 |
| *NETO2* | 7,1 | 7,9 | 57,5 | 19,1 | 24,7 | 1,3E-01 | 3,9E-01 | 0,89 | 4,5E-13 | 4,0E-11 | 0,12 | 7,1E-06 | 1,1E-04 | 0,37 | 1,7E-18 | 1,3E-16 | 0,29 |
| *FGFBP1* | 7,5 | 11,5 | 4,8 | 4,7 | 5,7 | 1,3E-01 | 3,9E-01 | 0,65 | 2,0E-01 | 4,3E-01 | 1,55 | 1,2E-01 | 2,5E-01 | 1,58 | 1,8E-01 | 2,8E-01 | 1,30 |
| *ABHD2* | 4,8 | 5,2 | 4,7 | 4,7 | 4,6 | 1,4E-01 | 3,9E-01 | 0,92 | 6,9E-01 | 8,5E-01 | 1,01 | 7,2E-01 | 8,5E-01 | 1,01 | 1,5E-01 | 2,5E-01 | 1,03 |
| *CDCP1* | 77,1 | 91,9 | 31,4 | 38,0 | 42,4 | 1,4E-01 | 3,9E-01 | 0,84 | 3,3E-06 | 5,1E-05 | 2,46 | 6,2E-06 | 1,1E-04 | 2,03 | 7,8E-08 | 7,6E-07 | 1,82 |
| *ALCAM* | 1001,2 | 861,5 | 734,8 | 854,3 | 849,2 | 1,4E-01 | 3,9E-01 | 1,16 | 2,3E-02 | 8,1E-02 | 1,36 | 2,5E-01 | 4,4E-01 | 1,17 | 8,4E-02 | 1,6E-01 | 1,18 |
| *NUDT11* | 21,0 | 13,2 | 8,0 | 18,0 | 10,6 | 1,4E-01 | 3,9E-01 | 1,59 | 2,9E-02 | 9,4E-02 | 2,63 | 7,3E-01 | 8,6E-01 | 1,17 | 7,2E-03 | 1,9E-02 | 1,98 |
| *TNFRSF21* | 170,8 | 246,5 | 28,1 | 34,0 | 45,7 | 1,4E-01 | 3,9E-01 | 0,69 | 9,9E-07 | 1,7E-05 | 6,09 | 4,8E-06 | 8,4E-05 | 5,02 | 1,9E-09 | 2,6E-08 | 3,73 |
| *PRELP* | 34,4 | 49,3 | 21,9 | 26,7 | 45,8 | 1,4E-01 | 3,9E-01 | 0,70 | 1,4E-01 | 3,3E-01 | 1,57 | 3,2E-01 | 5,3E-01 | 1,29 | 1,4E-01 | 2,5E-01 | 0,75 |
| *PAMR1* | 16,4 | 20,5 | 17,8 | 22,9 | 25,8 | 1,4E-01 | 3,9E-01 | 0,80 | 6,9E-01 | 8,5E-01 | 0,92 | 7,0E-02 | 1,6E-01 | 0,72 | 7,7E-04 | 2,8E-03 | 0,64 |
| *TSPAN4* | 239,4 | 276,3 | 232,6 | 217,8 | 220,6 | 1,4E-01 | 3,9E-01 | 0,87 | 8,3E-01 | 8,9E-01 | 1,03 | 4,4E-01 | 6,5E-01 | 1,10 | 3,3E-01 | 4,6E-01 | 1,09 |
| *EDA2R* | 4,9 | 4,7 | 4,9 | 4,8 | 4,7 | 1,4E-01 | 3,9E-01 | 1,06 | 9,4E-01 | 9,7E-01 | 1,00 | 5,0E-01 | 6,9E-01 | 1,03 | 8,2E-02 | 1,6E-01 | 1,06 |
| *ACSM3* | 4,7 | 4,7 | 4,7 | 4,7 | 4,7 | 1,4E-01 | 3,9E-01 | 0,99 | 1,4E-01 | 3,4E-01 | 0,99 | 9,8E-01 | 9,8E-01 | 1,00 | 4,7E-01 | 5,8E-01 | 1,00 |
| *PAPPA2* | 6,1 | 5,6 | 6,3 | 5,6 | 5,4 | 1,5E-01 | 4,1E-01 | 1,09 | 6,9E-01 | 8,5E-01 | 0,97 | 2,1E-01 | 3,9E-01 | 1,09 | 9,4E-03 | 2,4E-02 | 1,13 |
| *SFRP1* | 4,9 | 5,5 | 7,5 | 10,5 | 10,0 | 1,5E-01 | 4,1E-01 | 0,90 | 1,5E-02 | 5,6E-02 | 0,66 | 8,2E-04 | 5,2E-03 | 0,47 | 3,2E-07 | 2,6E-06 | 0,49 |
| *EFEMP2* | 195,7 | 249,5 | 186,2 | 153,6 | 219,4 | 1,5E-01 | 4,1E-01 | 0,78 | 8,4E-01 | 9,0E-01 | 1,05 | 3,4E-01 | 5,4E-01 | 1,27 | 4,5E-01 | 5,6E-01 | 0,89 |
| *KCNJ16* | 2212,5 | 1513,7 | 1987,0 | 2714,7 | 2468,0 | 1,5E-01 | 4,1E-01 | 1,46 | 6,8E-01 | 8,5E-01 | 1,11 | 1,7E-01 | 3,3E-01 | 0,81 | 2,8E-01 | 4,0E-01 | 0,90 |
| *FABP4* | 20,0 | 35,7 | 123,8 | 154,8 | 400,0 | 1,5E-01 | 4,1E-01 | 0,56 | 2,1E-03 | 1,2E-02 | 0,16 | 2,6E-04 | 2,1E-03 | 0,13 | 5,6E-15 | 2,3E-13 | 0,05 |
| *SLC12A2* | 37,7 | 34,1 | 31,6 | 35,0 | 31,5 | 1,6E-01 | 4,1E-01 | 1,10 | 6,6E-02 | 1,8E-01 | 1,19 | 4,4E-01 | 6,5E-01 | 1,08 | 2,3E-03 | 7,5E-03 | 1,20 |
| *GNG4* | 4,7 | 4,7 | 5,1 | 4,7 | 4,6 | 1,6E-01 | 4,1E-01 | 1,02 | 3,8E-02 | 1,2E-01 | 0,94 | 8,1E-01 | 8,8E-01 | 1,00 | 2,0E-02 | 4,8E-02 | 1,03 |
| *IGF2BP2* | 108,9 | 83,0 | 63,7 | 50,9 | 26,7 | 1,6E-01 | 4,1E-01 | 1,31 | 4,4E-02 | 1,3E-01 | 1,71 | 7,9E-03 | 3,2E-02 | 2,14 | 2,2E-11 | 4,0E-10 | 4,08 |
| *CYP24A1* | 5,8 | 8,1 | 11,4 | 7,9 | 5,9 | 1,6E-01 | 4,1E-01 | 0,72 | 2,6E-02 | 8,7E-02 | 0,51 | 3,7E-01 | 5,7E-01 | 0,73 | 9,1E-01 | 9,2E-01 | 0,98 |
| *SFTPD* | 7,8 | 9,3 | 17,2 | 11,0 | 9,6 | 1,6E-01 | 4,2E-01 | 0,85 | 4,7E-05 | 4,7E-04 | 0,46 | 2,6E-02 | 7,6E-02 | 0,71 | 8,6E-03 | 2,3E-02 | 0,81 |
| *PTGS1* | 19,4 | 24,7 | 11,7 | 13,3 | 14,6 | 1,6E-01 | 4,2E-01 | 0,79 | 1,1E-02 | 4,3E-02 | 1,66 | 2,5E-02 | 7,6E-02 | 1,46 | 1,8E-02 | 4,5E-02 | 1,33 |
| *HCN2* | 4,7 | 4,7 | 4,7 | 4,7 | 4,7 | 1,7E-01 | 4,3E-01 | 0,99 | 2,6E-01 | 4,9E-01 | 0,99 | 1,6E-01 | 3,2E-01 | 0,99 | 5,3E-01 | 6,4E-01 | 1,00 |
| *MLXIPL* | 5,2 | 4,7 | 5,4 | 4,9 | 4,5 | 1,7E-01 | 4,4E-01 | 1,10 | 8,0E-01 | 8,8E-01 | 0,97 | 4,3E-01 | 6,3E-01 | 1,08 | 1,7E-02 | 4,2E-02 | 1,16 |
| *RTN1* | 5,0 | 5,7 | 5,4 | 5,8 | 6,1 | 1,8E-01 | 4,5E-01 | 0,87 | 2,0E-01 | 4,4E-01 | 0,92 | 4,6E-02 | 1,1E-01 | 0,86 | 1,9E-03 | 6,2E-03 | 0,81 |
| *RASA2* | 5,3 | 5,2 | 5,2 | 5,1 | 5,3 | 1,8E-01 | 4,5E-01 | 1,02 | 2,9E-01 | 5,2E-01 | 1,03 | 8,3E-02 | 1,8E-01 | 1,04 | 5,3E-01 | 6,4E-01 | 1,01 |
| *SEMA4G* | 9,9 | 8,5 | 8,3 | 10,3 | 7,5 | 1,8E-01 | 4,6E-01 | 1,17 | 2,7E-01 | 5,0E-01 | 1,20 | 8,2E-01 | 8,9E-01 | 0,97 | 4,6E-03 | 1,3E-02 | 1,33 |
| *DPEP2* | 4,7 | 4,8 | 4,8 | 4,8 | 5,0 | 1,9E-01 | 4,8E-01 | 0,97 | 2,3E-01 | 4,5E-01 | 0,99 | 8,0E-03 | 3,2E-02 | 0,98 | 3,5E-02 | 8,1E-02 | 0,93 |
| *BTK* | 6,4 | 7,4 | 5,8 | 5,9 | 6,6 | 1,9E-01 | 4,8E-01 | 0,87 | 3,4E-01 | 5,9E-01 | 1,11 | 3,4E-01 | 5,4E-01 | 1,09 | 8,7E-01 | 9,0E-01 | 0,98 |
| *PTK2B* | 55,1 | 68,7 | 24,6 | 30,3 | 27,3 | 1,9E-01 | 4,8E-01 | 0,80 | 2,0E-03 | 1,2E-02 | 2,24 | 7,7E-03 | 3,1E-02 | 1,82 | 1,0E-05 | 6,1E-05 | 2,02 |
| *OXTR* | 5,1 | 5,4 | 4,8 | 4,8 | 4,9 | 1,9E-01 | 4,8E-01 | 0,93 | 3,4E-01 | 5,9E-01 | 1,06 | 3,2E-01 | 5,3E-01 | 1,06 | 3,5E-01 | 4,7E-01 | 1,04 |
| *IGFBP2* | 66,7 | 95,3 | 37,1 | 35,8 | 47,5 | 2,0E-01 | 4,8E-01 | 0,70 | 1,4E-01 | 3,3E-01 | 1,80 | 8,2E-02 | 1,8E-01 | 1,86 | 1,7E-01 | 2,7E-01 | 1,41 |
| *PLIN1* | 4,7 | 4,8 | 4,7 | 4,7 | 4,8 | 2,0E-01 | 4,8E-01 | 0,98 | 7,6E-01 | 8,7E-01 | 1,00 | 9,5E-01 | 9,6E-01 | 1,00 | 1,2E-01 | 2,2E-01 | 0,98 |
| *B3GALT2* | 4,7 | 4,7 | 7,3 | 4,8 | 5,9 | 2,0E-01 | 4,8E-01 | 1,01 | 3,4E-03 | 1,7E-02 | 0,64 | 5,6E-02 | 1,4E-01 | 0,98 | 2,3E-02 | 5,6E-02 | 0,79 |
| *DNAJC13* | 218,6 | 197,1 | 239,6 | 225,1 | 275,9 | 2,0E-01 | 4,9E-01 | 1,11 | 4,9E-01 | 7,4E-01 | 0,91 | 7,5E-01 | 8,6E-01 | 0,97 | 1,7E-03 | 5,6E-03 | 0,79 |
| *ASPA* | 4,7 | 4,9 | 4,8 | 4,9 | 5,4 | 2,0E-01 | 4,9E-01 | 0,96 | 2,4E-01 | 4,6E-01 | 0,98 | 2,1E-02 | 6,4E-02 | 0,96 | 3,9E-03 | 1,1E-02 | 0,87 |
| *ARHGEF10* | 5,1 | 4,7 | 4,6 | 4,8 | 4,8 | 2,0E-01 | 4,9E-01 | 1,08 | 2,7E-01 | 5,0E-01 | 1,10 | 5,4E-01 | 7,3E-01 | 1,05 | 2,3E-01 | 3,5E-01 | 1,06 |
| *ROS1* | 4,7 | 4,8 | 4,7 | 4,7 | 4,6 | 2,0E-01 | 4,9E-01 | 0,98 | 8,0E-01 | 8,8E-01 | 1,00 | 9,2E-01 | 9,5E-01 | 1,00 | 5,6E-02 | 1,2E-01 | 1,02 |
| *MPPED2* | 6,6 | 9,3 | 77,1 | 70,4 | 163,8 | 2,1E-01 | 4,9E-01 | 0,71 | 5,8E-08 | 1,2E-06 | 0,09 | 2,6E-07 | 8,8E-06 | 0,09 | 1,0E-23 | 1,3E-21 | 0,04 |
| *HEY2* | 103,8 | 144,1 | 78,0 | 90,5 | 35,1 | 2,1E-01 | 5,0E-01 | 0,72 | 4,2E-01 | 6,7E-01 | 1,33 | 6,7E-01 | 8,4E-01 | 1,15 | 5,0E-07 | 3,9E-06 | 2,95 |
| *ACVRL1* | 8,5 | 8,0 | 8,6 | 8,8 | 10,7 | 2,1E-01 | 5,0E-01 | 1,05 | 7,3E-01 | 8,7E-01 | 0,98 | 3,5E-01 | 5,4E-01 | 0,96 | 3,1E-04 | 1,2E-03 | 0,79 |
| *ADAMTSL2* | 4,7 | 4,7 | 4,7 | 4,7 | 4,8 | 2,1E-01 | 5,0E-01 | 1,00 | 1,8E-03 | 1,1E-02 | 0,99 | 1,9E-03 | 1,1E-02 | 0,99 | 2,5E-01 | 3,6E-01 | 0,98 |
| *CFD* | 38,2 | 56,7 | 38,2 | 54,8 | 204,2 | 2,1E-01 | 5,0E-01 | 0,67 | 1,0E+00 | 1,0E+00 | 1,00 | 3,3E-01 | 5,3E-01 | 0,70 | 1,3E-08 | 1,5E-07 | 0,19 |
| *MAPK10* | 30,7 | 33,4 | 59,7 | 39,5 | 36,4 | 2,1E-01 | 5,0E-01 | 0,92 | 3,7E-06 | 5,6E-05 | 0,51 | 1,5E-02 | 5,2E-02 | 0,78 | 3,3E-03 | 9,8E-03 | 0,84 |
| *ETV1* | 9,1 | 8,2 | 10,1 | 7,6 | 7,7 | 2,2E-01 | 5,0E-01 | 1,11 | 4,3E-01 | 6,8E-01 | 0,91 | 7,7E-02 | 1,7E-01 | 1,20 | 9,7E-03 | 2,5E-02 | 1,18 |
| *MYL1* | 7,2 | 4,5 | 5,5 | 6,0 | 4,9 | 2,2E-01 | 5,0E-01 | 1,61 | 6,4E-01 | 8,5E-01 | 1,31 | 7,0E-01 | 8,5E-01 | 1,21 | 3,0E-01 | 4,2E-01 | 1,47 |
| *TTC12* | 20,5 | 25,7 | 44,4 | 32,9 | 37,9 | 2,2E-01 | 5,0E-01 | 0,80 | 3,9E-03 | 1,9E-02 | 0,46 | 4,5E-02 | 1,1E-01 | 0,62 | 2,0E-04 | 8,2E-04 | 0,54 |
| *RNF39* | 4,8 | 4,7 | 4,7 | 4,7 | 4,7 | 2,2E-01 | 5,1E-01 | 1,02 | 4,0E-01 | 6,6E-01 | 1,02 | 3,5E-01 | 5,4E-01 | 1,02 | 8,6E-02 | 1,7E-01 | 1,02 |
| *NPTX2* | 5,0 | 5,7 | 4,9 | 7,5 | 4,8 | 2,2E-01 | 5,1E-01 | 0,88 | 7,5E-01 | 8,7E-01 | 1,04 | 2,5E-02 | 7,6E-02 | 0,67 | 4,0E-01 | 5,2E-01 | 1,06 |
| *GPD2* | 8,0 | 7,0 | 9,6 | 10,1 | 7,7 | 2,2E-01 | 5,1E-01 | 1,14 | 2,4E-01 | 4,7E-01 | 0,83 | 8,6E-02 | 1,9E-01 | 0,79 | 7,2E-01 | 7,9E-01 | 1,03 |
| *ALPL* | 5,1 | 5,9 | 5,0 | 5,1 | 4,4 | 2,2E-01 | 5,1E-01 | 0,87 | 6,9E-01 | 8,5E-01 | 1,04 | 9,1E-01 | 9,4E-01 | 1,01 | 4,5E-03 | 1,3E-02 | 1,16 |
| *PYGM* | 6,4 | 4,5 | 7,2 | 5,6 | 5,1 | 2,3E-01 | 5,1E-01 | 1,41 | 7,7E-01 | 8,7E-01 | 0,88 | 7,0E-01 | 8,5E-01 | 1,15 | 3,9E-01 | 5,0E-01 | 1,26 |
| *MID1IP1* | 66,1 | 77,3 | 58,3 | 69,1 | 57,4 | 2,3E-01 | 5,2E-01 | 0,86 | 5,0E-01 | 7,4E-01 | 1,13 | 8,2E-01 | 8,9E-01 | 0,96 | 2,2E-01 | 3,3E-01 | 1,15 |
| *CYSLTR1* | 4,7 | 4,7 | 4,7 | 4,7 | 4,8 | 2,3E-01 | 5,2E-01 | 1,00 | 3,5E-01 | 6,0E-01 | 1,00 | 2,3E-02 | 6,9E-02 | 1,00 | 2,4E-01 | 3,6E-01 | 0,98 |
| *PIK3CG* | 4,7 | 4,8 | 4,7 | 4,8 | 4,9 | 2,3E-01 | 5,2E-01 | 0,97 | 7,1E-01 | 8,6E-01 | 1,00 | 2,7E-01 | 4,7E-01 | 0,99 | 1,2E-01 | 2,2E-01 | 0,96 |
| *EGR1* | 762,3 | 936,0 | 646,7 | 671,9 | 975,6 | 2,4E-01 | 5,3E-01 | 0,81 | 4,9E-01 | 7,4E-01 | 1,18 | 5,2E-01 | 7,1E-01 | 1,13 | 8,6E-02 | 1,6E-01 | 0,78 |
| *SLC6A12* | 6,2 | 5,4 | 4,7 | 4,8 | 5,2 | 2,4E-01 | 5,3E-01 | 1,15 | 1,0E-01 | 2,6E-01 | 1,31 | 7,4E-02 | 1,7E-01 | 1,29 | 5,7E-02 | 1,2E-01 | 1,20 |
| *PCK1* | 4,7 | 4,7 | 4,7 | 4,7 | 4,7 | 2,4E-01 | 5,3E-01 | 1,00 | 1,9E-03 | 1,1E-02 | 0,99 | 2,7E-02 | 7,8E-02 | 1,00 | 1,3E-01 | 2,3E-01 | 0,99 |
| *CDO1* | 82,9 | 59,8 | 39,6 | 41,7 | 79,7 | 2,4E-01 | 5,4E-01 | 1,39 | 6,1E-02 | 1,7E-01 | 2,10 | 4,1E-02 | 1,0E-01 | 1,99 | 8,6E-01 | 8,9E-01 | 1,04 |
| *LAMB1* | 449,3 | 546,0 | 236,0 | 343,2 | 423,9 | 2,5E-01 | 5,4E-01 | 0,82 | 7,3E-03 | 3,2E-02 | 1,90 | 3,3E-01 | 5,3E-01 | 1,31 | 6,8E-01 | 7,6E-01 | 1,06 |
| *CCL7* | 6,1 | 5,6 | 5,5 | 5,7 | 5,5 | 2,5E-01 | 5,5E-01 | 1,09 | 4,0E-01 | 6,6E-01 | 1,10 | 5,6E-01 | 7,4E-01 | 1,06 | 1,4E-01 | 2,4E-01 | 1,10 |
| *TFCP2L1* | 5,0 | 4,6 | 5,6 | 6,0 | 11,1 | 2,5E-01 | 5,5E-01 | 1,08 | 2,0E-01 | 4,3E-01 | 0,89 | 1,5E-02 | 5,2E-02 | 0,83 | 5,4E-09 | 6,9E-08 | 0,45 |
| *LYST* | 119,5 | 103,9 | 37,1 | 70,4 | 73,4 | 2,6E-01 | 5,5E-01 | 1,15 | 4,8E-08 | 1,2E-06 | 3,22 | 1,7E-03 | 9,9E-03 | 1,70 | 1,1E-04 | 4,7E-04 | 1,63 |
| *NAV2* | 951,1 | 750,8 | 675,5 | 444,1 | 498,9 | 2,6E-01 | 5,5E-01 | 1,27 | 1,9E-01 | 4,1E-01 | 1,41 | 1,8E-03 | 1,0E-02 | 2,14 | 1,3E-04 | 5,4E-04 | 1,91 |
| *FHL2* | 78,9 | 115,2 | 33,7 | 33,2 | 183,6 | 2,6E-01 | 5,6E-01 | 0,68 | 8,3E-02 | 2,2E-01 | 2,34 | 3,8E-02 | 9,9E-02 | 2,38 | 2,1E-03 | 6,7E-03 | 0,43 |
| *GRB14* | 4,8 | 5,0 | 5,0 | 5,9 | 6,7 | 2,6E-01 | 5,6E-01 | 0,95 | 8,7E-02 | 2,2E-01 | 0,96 | 2,4E-03 | 1,2E-02 | 0,81 | 1,0E-04 | 4,5E-04 | 0,71 |
| *GLRX5* | 432,2 | 470,4 | 606,0 | 517,6 | 562,0 | 2,6E-01 | 5,6E-01 | 0,92 | 1,9E-03 | 1,1E-02 | 0,71 | 5,5E-02 | 1,4E-01 | 0,83 | 4,6E-04 | 1,8E-03 | 0,77 |
| *GCNT4* | 4,8 | 4,7 | 4,7 | 4,7 | 4,7 | 2,6E-01 | 5,6E-01 | 1,03 | 4,7E-01 | 7,2E-01 | 1,03 | 4,1E-01 | 6,1E-01 | 1,03 | 1,6E-01 | 2,7E-01 | 1,03 |
| *ALMS1* | 39,5 | 36,0 | 28,5 | 32,3 | 42,8 | 2,6E-01 | 5,6E-01 | 1,10 | 6,9E-03 | 3,1E-02 | 1,38 | 8,4E-02 | 1,8E-01 | 1,22 | 2,8E-01 | 4,1E-01 | 0,92 |
| *PGM5* | 4,7 | 4,7 | 4,7 | 4,8 | 5,0 | 2,6E-01 | 5,6E-01 | 1,01 | 2,6E-01 | 4,9E-01 | 0,99 | 2,7E-02 | 7,9E-02 | 0,99 | 4,6E-03 | 1,3E-02 | 0,94 |
| *PNP* | 247,9 | 167,4 | 52,0 | 52,9 | 74,7 | 2,7E-01 | 5,6E-01 | 1,48 | 1,8E-03 | 1,1E-02 | 4,76 | 9,5E-04 | 5,8E-03 | 4,68 | 7,9E-05 | 3,5E-04 | 3,32 |
| *ZFP36L2* | 1451,8 | 1682,1 | 1949,1 | 1934,7 | 3197,7 | 2,7E-01 | 5,6E-01 | 0,86 | 1,1E-01 | 2,7E-01 | 0,74 | 1,1E-01 | 2,2E-01 | 0,75 | 4,4E-11 | 7,3E-10 | 0,45 |
| *VASH2* | 4,7 | 4,7 | 4,7 | 4,7 | 4,7 | 2,8E-01 | 5,6E-01 | 1,00 | 2,1E-01 | 4,4E-01 | 1,00 | 3,6E-02 | 9,6E-02 | 1,00 | 1,6E-05 | 8,4E-05 | 1,00 |
| *NPPC* | 4,7 | 4,7 | 4,6 | 5,6 | 4,8 | 2,8E-01 | 5,6E-01 | 0,99 | 5,5E-01 | 8,0E-01 | 1,01 | 3,6E-02 | 9,6E-02 | 0,83 | 1,6E-05 | 8,4E-05 | 0,98 |
| *GPX2* | 4,6 | 4,7 | 4,4 | 9,5 | 5,0 | 2,8E-01 | 5,6E-01 | 0,97 | 2,1E-01 | 4,4E-01 | 1,04 | 3,6E-02 | 9,6E-02 | 0,48 | 1,6E-05 | 8,4E-05 | 0,92 |
| *PIP5K1B* | 4,7 | 4,7 | 4,7 | 4,7 | 4,7 | 2,8E-01 | 5,6E-01 | 1,00 | 2,1E-01 | 4,4E-01 | 1,00 | 3,6E-02 | 9,6E-02 | 1,00 | 1,6E-05 | 8,4E-05 | 1,00 |
| *RALYL* | 4,7 | 4,7 | 4,7 | 4,7 | 4,7 | 2,8E-01 | 5,6E-01 | 1,00 | 2,1E-01 | 4,4E-01 | 1,00 | 1,8E-02 | 5,9E-02 | 1,00 | 2,4E-01 | 3,6E-01 | 1,00 |
| *PCDH7* | 4,7 | 4,7 | 4,8 | 4,7 | 4,7 | 2,8E-01 | 5,6E-01 | 1,00 | 1,4E-02 | 5,2E-02 | 0,98 | 6,1E-04 | 4,1E-03 | 0,99 | 1,6E-05 | 8,4E-05 | 1,00 |
| *PIGZ* | 5,1 | 4,8 | 4,9 | 5,1 | 4,7 | 2,8E-01 | 5,6E-01 | 1,07 | 5,8E-01 | 8,1E-01 | 1,05 | 9,8E-01 | 9,8E-01 | 1,00 | 1,6E-01 | 2,7E-01 | 1,08 |
| *OSMR* | 10,2 | 7,9 | 4,4 | 5,0 | 6,9 | 2,9E-01 | 5,8E-01 | 1,29 | 1,4E-02 | 5,3E-02 | 2,34 | 1,8E-02 | 5,9E-02 | 2,05 | 4,4E-02 | 9,8E-02 | 1,49 |
| *PILRA* | 9,6 | 10,6 | 8,3 | 8,6 | 8,6 | 3,0E-01 | 5,9E-01 | 0,91 | 1,6E-01 | 3,6E-01 | 1,16 | 2,0E-01 | 3,8E-01 | 1,12 | 8,3E-02 | 1,6E-01 | 1,12 |
| *STRA6* | 10,4 | 13,0 | 4,9 | 5,7 | 6,5 | 3,0E-01 | 5,9E-01 | 0,80 | 7,6E-03 | 3,2E-02 | 2,10 | 1,6E-02 | 5,4E-02 | 1,82 | 3,0E-03 | 9,4E-03 | 1,59 |
| *EHF* | 9,1 | 6,4 | 3,7 | 3,3 | 6,5 | 3,0E-01 | 6,0E-01 | 1,41 | 5,7E-02 | 1,6E-01 | 2,47 | 1,5E-02 | 5,3E-02 | 2,73 | 2,0E-01 | 3,2E-01 | 1,41 |
| *TRPM3* | 4,8 | 4,7 | 4,9 | 4,7 | 4,7 | 3,0E-01 | 6,0E-01 | 1,02 | 3,0E-01 | 5,4E-01 | 0,97 | 7,2E-01 | 8,5E-01 | 1,01 | 9,3E-02 | 1,8E-01 | 1,02 |
| *EDNRA* | 82,1 | 98,3 | 55,2 | 63,5 | 75,1 | 3,0E-01 | 6,0E-01 | 0,83 | 9,3E-02 | 2,4E-01 | 1,49 | 2,7E-01 | 4,7E-01 | 1,29 | 5,1E-01 | 6,2E-01 | 1,09 |
| *ATP10A* | 4,7 | 4,7 | 4,7 | 4,7 | 4,7 | 3,0E-01 | 6,0E-01 | 1,00 | 5,7E-01 | 8,0E-01 | 1,00 | 5,5E-01 | 7,4E-01 | 1,00 | 1,2E-01 | 2,2E-01 | 1,00 |
| *PAPSS2* | 12,6 | 15,6 | 13,8 | 23,3 | 80,3 | 3,0E-01 | 6,0E-01 | 0,80 | 7,4E-01 | 8,7E-01 | 0,91 | 1,2E-02 | 4,4E-02 | 0,54 | 3,8E-15 | 1,7E-13 | 0,16 |
| *SLC5A3* | 1517,1 | 1227,6 | 925,4 | 1408,4 | 1679,7 | 3,1E-01 | 6,0E-01 | 1,24 | 6,0E-02 | 1,7E-01 | 1,64 | 7,4E-01 | 8,6E-01 | 1,08 | 5,2E-01 | 6,2E-01 | 0,90 |
| *AMPH* | 4,7 | 4,7 | 4,7 | 4,7 | 4,7 | 3,1E-01 | 6,0E-01 | 1,01 | 6,5E-01 | 8,5E-01 | 1,01 | 6,8E-01 | 8,4E-01 | 1,00 | 1,2E-01 | 2,1E-01 | 1,01 |
| *KCND3* | 4,7 | 4,8 | 4,7 | 4,7 | 4,7 | 3,1E-01 | 6,0E-01 | 0,99 | 8,2E-01 | 8,9E-01 | 1,00 | 9,1E-01 | 9,3E-01 | 1,00 | 1,6E-01 | 2,7E-01 | 1,01 |
| *ARNT2* | 29,1 | 37,9 | 67,7 | 30,8 | 30,5 | 3,1E-01 | 6,0E-01 | 0,77 | 3,7E-02 | 1,1E-01 | 0,43 | 8,6E-01 | 9,1E-01 | 0,94 | 8,2E-01 | 8,7E-01 | 0,95 |
| *CYP39A1* | 8,6 | 10,0 | 7,0 | 10,6 | 14,1 | 3,1E-01 | 6,0E-01 | 0,86 | 3,1E-01 | 5,5E-01 | 1,24 | 3,1E-01 | 5,2E-01 | 0,81 | 5,4E-04 | 2,0E-03 | 0,61 |
| *MAP6D1* | 4,8 | 4,7 | 4,9 | 5,0 | 4,7 | 3,1E-01 | 6,0E-01 | 1,02 | 4,4E-01 | 6,9E-01 | 0,98 | 1,5E-01 | 3,0E-01 | 0,96 | 5,5E-01 | 6,6E-01 | 1,01 |
| *SFXN3* | 151,1 | 163,3 | 66,0 | 102,6 | 76,7 | 3,1E-01 | 6,0E-01 | 0,93 | 1,4E-08 | 4,0E-07 | 2,29 | 2,5E-04 | 2,0E-03 | 1,47 | 1,7E-14 | 5,6E-13 | 1,97 |
| *CTSS* | 256,3 | 198,1 | 88,0 | 105,1 | 113,6 | 3,2E-01 | 6,1E-01 | 1,29 | 3,2E-03 | 1,6E-02 | 2,91 | 9,0E-03 | 3,5E-02 | 2,44 | 9,9E-04 | 3,5E-03 | 2,26 |
| *NDUFB6* | 592,6 | 626,1 | 818,1 | 753,6 | 808,8 | 3,2E-01 | 6,1E-01 | 0,95 | 4,5E-05 | 4,6E-04 | 0,72 | 4,9E-04 | 3,5E-03 | 0,79 | 2,0E-08 | 2,1E-07 | 0,73 |
| *CHN2* | 17,8 | 16,3 | 31,0 | 16,9 | 17,4 | 3,2E-01 | 6,1E-01 | 1,09 | 2,8E-02 | 9,2E-02 | 0,57 | 7,1E-01 | 8,5E-01 | 1,05 | 7,9E-01 | 8,5E-01 | 1,02 |
| *FAM60A* | 583,9 | 647,6 | 561,8 | 493,2 | 451,4 | 3,3E-01 | 6,2E-01 | 0,90 | 8,0E-01 | 8,8E-01 | 1,04 | 2,6E-01 | 4,6E-01 | 1,18 | 8,7E-03 | 2,3E-02 | 1,29 |
| *HPGD* | 8,4 | 9,0 | 18,9 | 11,4 | 9,6 | 3,3E-01 | 6,2E-01 | 0,93 | 2,4E-05 | 2,6E-04 | 0,44 | 1,7E-02 | 5,9E-02 | 0,73 | 7,2E-02 | 1,5E-01 | 0,87 |
| *ACACB* | 30,8 | 36,7 | 107,1 | 100,2 | 104,7 | 3,3E-01 | 6,2E-01 | 0,84 | 5,2E-06 | 7,5E-05 | 0,29 | 8,6E-06 | 1,2E-04 | 0,31 | 1,6E-11 | 3,0E-10 | 0,29 |
| *DSC2* | 17,4 | 14,4 | 7,9 | 9,0 | 10,1 | 3,3E-01 | 6,2E-01 | 1,20 | 3,1E-03 | 1,6E-02 | 2,19 | 4,3E-03 | 2,0E-02 | 1,93 | 4,5E-04 | 1,7E-03 | 1,73 |
| *SERINC5* | 232,8 | 208,5 | 167,6 | 139,0 | 161,6 | 3,4E-01 | 6,2E-01 | 1,12 | 6,1E-02 | 1,7E-01 | 1,39 | 1,1E-03 | 6,3E-03 | 1,67 | 2,0E-04 | 8,2E-04 | 1,44 |
| *ABAT* | 554,6 | 444,5 | 1632,8 | 1731,8 | 943,2 | 3,4E-01 | 6,2E-01 | 1,25 | 2,4E-04 | 1,9E-03 | 0,34 | 1,6E-05 | 2,1E-04 | 0,32 | 3,2E-03 | 9,8E-03 | 0,59 |
| *ANGPT2* | 11,2 | 13,2 | 34,5 | 22,1 | 12,5 | 3,4E-01 | 6,2E-01 | 0,85 | 7,0E-06 | 9,3E-05 | 0,32 | 4,1E-03 | 1,9E-02 | 0,51 | 3,8E-01 | 4,9E-01 | 0,89 |
| *DENND3* | 4,9 | 5,2 | 4,7 | 4,7 | 4,6 | 3,4E-01 | 6,2E-01 | 0,95 | 3,7E-01 | 6,2E-01 | 1,04 | 3,2E-01 | 5,2E-01 | 1,04 | 5,4E-02 | 1,1E-01 | 1,06 |
| *PLN* | 11,1 | 15,3 | 10,4 | 11,3 | 35,4 | 3,4E-01 | 6,3E-01 | 0,73 | 8,9E-01 | 9,3E-01 | 1,07 | 9,7E-01 | 9,8E-01 | 0,99 | 1,7E-04 | 7,0E-04 | 0,31 |
| *BMP15* | 4,7 | 4,7 | 4,7 | 4,7 | 4,7 | 3,4E-01 | 6,3E-01 | 1,01 | 6,6E-01 | 8,5E-01 | 1,01 | 9,7E-01 | 9,8E-01 | 1,00 | 1,6E-01 | 2,6E-01 | 1,01 |
| *TACR3* | 4,7 | 4,7 | 4,7 | 4,7 | 4,7 | 3,5E-01 | 6,4E-01 | 1,00 | 6,3E-01 | 8,5E-01 | 1,00 | 5,7E-01 | 7,5E-01 | 1,00 | 4,9E-01 | 6,0E-01 | 1,00 |
| *RPS6KA2* | 237,8 | 204,5 | 159,3 | 212,1 | 120,1 | 3,5E-01 | 6,4E-01 | 1,16 | 4,8E-02 | 1,4E-01 | 1,49 | 5,0E-01 | 6,9E-01 | 1,12 | 7,6E-07 | 5,6E-06 | 1,98 |
| *DLK1* | 4,7 | 4,7 | 4,7 | 4,7 | 4,7 | 3,5E-01 | 6,4E-01 | 1,01 | 6,8E-01 | 8,5E-01 | 1,01 | 7,1E-01 | 8,5E-01 | 1,01 | 1,5E-01 | 2,6E-01 | 1,01 |
| *DHRS9* | 9,2 | 10,9 | 6,1 | 6,0 | 8,4 | 3,5E-01 | 6,4E-01 | 0,85 | 5,8E-02 | 1,7E-01 | 1,51 | 2,4E-02 | 7,3E-02 | 1,53 | 4,5E-01 | 5,6E-01 | 1,11 |
| *NCF2* | 17,2 | 22,2 | 7,0 | 8,7 | 11,8 | 3,6E-01 | 6,4E-01 | 0,78 | 8,4E-03 | 3,5E-02 | 2,47 | 2,0E-02 | 6,4E-02 | 1,98 | 8,0E-02 | 1,6E-01 | 1,46 |
| *SLC24A2* | 4,7 | 4,7 | 4,7 | 4,7 | 4,7 | 3,6E-01 | 6,4E-01 | 1,01 | 7,0E-01 | 8,6E-01 | 1,00 | 8,9E-01 | 9,2E-01 | 1,00 | 1,7E-01 | 2,7E-01 | 1,01 |
| *GJA1* | 1968,5 | 2360,4 | 1626,0 | 1868,8 | 3350,5 | 3,6E-01 | 6,4E-01 | 0,83 | 5,0E-01 | 7,4E-01 | 1,21 | 8,3E-01 | 8,9E-01 | 1,05 | 1,8E-03 | 5,9E-03 | 0,59 |
| *GPR63* | 4,7 | 4,7 | 4,7 | 4,7 | 4,7 | 3,6E-01 | 6,4E-01 | 1,00 | 6,8E-01 | 8,5E-01 | 1,00 | 7,1E-01 | 8,5E-01 | 1,00 | 1,6E-01 | 2,6E-01 | 1,01 |
| *TIMP1* | 4452,4 | 4820,9 | 987,0 | 420,1 | 897,2 | 3,6E-01 | 6,4E-01 | 0,92 | 2,2E-15 | 2,9E-13 | 4,51 | 4,9E-08 | 2,4E-06 | 10,60 | 5,1E-21 | 4,6E-19 | 4,96 |
| *VSIG4* | 47,4 | 60,6 | 18,6 | 23,1 | 33,6 | 3,8E-01 | 6,7E-01 | 0,78 | 1,0E-02 | 4,1E-02 | 2,55 | 2,4E-02 | 7,3E-02 | 2,05 | 1,5E-01 | 2,5E-01 | 1,41 |
| *SLC44A4* | 4,8 | 4,7 | 6,4 | 5,1 | 4,8 | 3,8E-01 | 6,7E-01 | 1,03 | 2,3E-02 | 7,9E-02 | 0,76 | 3,5E-01 | 5,4E-01 | 0,95 | 9,1E-01 | 9,2E-01 | 1,00 |
| *RAB27B* | 4,7 | 4,7 | 4,8 | 4,7 | 4,7 | 3,8E-01 | 6,7E-01 | 1,01 | 1,5E-01 | 3,5E-01 | 0,98 | 4,4E-01 | 6,4E-01 | 1,01 | 3,4E-01 | 4,6E-01 | 1,01 |
| *FA2H* | 5,0 | 4,7 | 7,7 | 5,3 | 4,9 | 3,8E-01 | 6,7E-01 | 1,06 | 3,1E-03 | 1,6E-02 | 0,65 | 4,9E-01 | 6,9E-01 | 0,94 | 9,0E-01 | 9,2E-01 | 1,01 |
| *BRAF* | 10,7 | 10,0 | 12,4 | 11,3 | 10,3 | 3,8E-01 | 6,7E-01 | 1,07 | 2,5E-01 | 4,8E-01 | 0,86 | 5,9E-01 | 7,7E-01 | 0,94 | 5,7E-01 | 6,7E-01 | 1,04 |
| *SELP* | 19,1 | 22,8 | 12,2 | 14,5 | 17,5 | 3,8E-01 | 6,7E-01 | 0,84 | 8,4E-02 | 2,2E-01 | 1,57 | 2,4E-01 | 4,3E-01 | 1,32 | 5,9E-01 | 6,9E-01 | 1,09 |
| *P2RX6* | 4,8 | 4,7 | 4,7 | 4,7 | 4,7 | 3,8E-01 | 6,7E-01 | 1,02 | 6,2E-01 | 8,4E-01 | 1,02 | 6,1E-01 | 7,9E-01 | 1,02 | 1,4E-01 | 2,4E-01 | 1,04 |
| *DDT* | 128,3 | 141,7 | 143,2 | 117,4 | 169,8 | 3,8E-01 | 6,7E-01 | 0,91 | 5,4E-01 | 7,9E-01 | 0,90 | 5,5E-01 | 7,4E-01 | 1,09 | 6,9E-03 | 1,9E-02 | 0,76 |
| *CLIC5* | 58,7 | 77,7 | 17,2 | 27,7 | 53,1 | 3,9E-01 | 6,8E-01 | 0,76 | 2,6E-03 | 1,4E-02 | 3,42 | 4,2E-02 | 1,1E-01 | 2,12 | 6,6E-01 | 7,4E-01 | 1,11 |
| *KCNC3* | 4,7 | 4,7 | 4,7 | 4,7 | 4,7 | 4,0E-01 | 6,8E-01 | 1,00 | 6,8E-01 | 8,5E-01 | 1,00 | 7,1E-01 | 8,5E-01 | 1,00 | 1,5E-01 | 2,6E-01 | 1,00 |
| *PKNOX2* | 4,8 | 4,7 | 4,7 | 4,7 | 4,7 | 4,0E-01 | 6,8E-01 | 1,01 | 6,9E-01 | 8,5E-01 | 1,01 | 8,9E-01 | 9,2E-01 | 1,00 | 5,4E-01 | 6,4E-01 | 1,01 |
| *CNTFR* | 7,1 | 7,1 | 7,1 | 8,1 | 7,2 | 4,0E-01 | 6,9E-01 | 1,00 | 2,2E-01 | 4,4E-01 | 1,01 | 3,7E-02 | 9,7E-02 | 0,88 | 6,5E-04 | 2,3E-03 | 0,99 |
| *GABRG3* | 4,9 | 4,7 | 7,6 | 5,0 | 4,8 | 4,0E-01 | 6,9E-01 | 1,05 | 5,2E-03 | 2,4E-02 | 0,65 | 9,4E-01 | 9,6E-01 | 0,99 | 7,0E-01 | 7,8E-01 | 1,02 |
| *PTGS2* | 61,8 | 86,7 | 6,4 | 9,0 | 45,1 | 4,1E-01 | 7,0E-01 | 0,71 | 5,7E-05 | 5,4E-04 | 9,67 | 1,2E-04 | 1,2E-03 | 6,84 | 3,7E-01 | 4,8E-01 | 1,37 |
| *ST8SIA3* | 5,0 | 4,7 | 4,8 | 4,8 | 4,5 | 4,1E-01 | 7,0E-01 | 1,08 | 7,1E-01 | 8,6E-01 | 1,05 | 7,3E-01 | 8,6E-01 | 1,04 | 1,9E-01 | 3,1E-01 | 1,11 |
| *ST3GAL1* | 67,6 | 80,1 | 181,5 | 111,0 | 65,9 | 4,2E-01 | 7,0E-01 | 0,84 | 8,0E-04 | 5,7E-03 | 0,37 | 7,9E-02 | 1,8E-01 | 0,61 | 8,8E-01 | 9,1E-01 | 1,03 |
| *ATP1A2* | 6,7 | 5,6 | 6,1 | 6,0 | 6,4 | 4,2E-01 | 7,0E-01 | 1,20 | 7,7E-01 | 8,7E-01 | 1,10 | 7,0E-01 | 8,5E-01 | 1,11 | 8,5E-01 | 8,9E-01 | 1,04 |
| *CAMKV* | 4,7 | 4,7 | 4,7 | 4,7 | 4,7 | 4,2E-01 | 7,0E-01 | 1,00 | 7,2E-01 | 8,7E-01 | 1,00 | 7,4E-01 | 8,6E-01 | 1,00 | 2,2E-01 | 3,3E-01 | 1,01 |
| *TMEM97* | 14,5 | 13,0 | 26,3 | 12,0 | 17,7 | 4,2E-01 | 7,1E-01 | 1,12 | 1,4E-02 | 5,2E-02 | 0,55 | 2,3E-01 | 4,1E-01 | 1,21 | 9,6E-02 | 1,8E-01 | 0,82 |
| *DUSP5* | 1069,6 | 1260,5 | 84,7 | 74,1 | 161,5 | 4,3E-01 | 7,1E-01 | 0,85 | 1,9E-11 | 1,3E-09 | 12,62 | 4,6E-12 | 8,1E-10 | 14,43 | 3,2E-11 | 5,4E-10 | 6,62 |
| *GIPC2* | 5,0 | 4,7 | 4,8 | 4,8 | 4,6 | 4,3E-01 | 7,1E-01 | 1,07 | 8,1E-01 | 8,8E-01 | 1,03 | 7,6E-01 | 8,6E-01 | 1,03 | 2,9E-01 | 4,2E-01 | 1,08 |
| *SLC16A4* | 42,1 | 45,8 | 43,5 | 44,1 | 35,8 | 4,3E-01 | 7,2E-01 | 0,92 | 8,4E-01 | 9,0E-01 | 0,97 | 7,8E-01 | 8,7E-01 | 0,95 | 6,5E-02 | 1,4E-01 | 1,18 |
| *IL17RB* | 5,1 | 5,0 | 6,5 | 5,0 | 5,4 | 4,3E-01 | 7,2E-01 | 1,02 | 2,0E-03 | 1,2E-02 | 0,78 | 2,8E-01 | 4,8E-01 | 1,04 | 1,3E-01 | 2,3E-01 | 0,96 |
| *FETUB* | 4,7 | 4,7 | 4,7 | 4,7 | 4,7 | 4,4E-01 | 7,2E-01 | 1,00 | 5,8E-01 | 8,1E-01 | 1,00 | 5,2E-01 | 7,1E-01 | 1,00 | 3,9E-01 | 5,0E-01 | 1,00 |
| *F7* | 4,7 | 4,7 | 4,7 | 4,7 | 4,7 | 4,4E-01 | 7,2E-01 | 1,00 | 5,5E-01 | 8,0E-01 | 1,00 | 4,7E-01 | 6,8E-01 | 1,00 | 4,1E-01 | 5,2E-01 | 1,00 |
| *NCOA4* | 1708,3 | 1618,1 | 1832,6 | 1602,3 | 2001,8 | 4,4E-01 | 7,2E-01 | 1,06 | 4,0E-01 | 6,6E-01 | 0,93 | 4,0E-01 | 6,1E-01 | 1,07 | 4,0E-03 | 1,1E-02 | 0,85 |
| *TYROBP* | 271,8 | 326,5 | 60,3 | 75,3 | 134,0 | 4,4E-01 | 7,2E-01 | 0,83 | 2,0E-05 | 2,4E-04 | 4,51 | 1,3E-04 | 1,2E-03 | 3,61 | 3,3E-03 | 1,0E-02 | 2,03 |
| *NDRG2* | 8,0 | 7,0 | 16,4 | 20,0 | 20,0 | 4,4E-01 | 7,2E-01 | 1,14 | 1,2E-02 | 4,8E-02 | 0,49 | 9,4E-05 | 9,3E-04 | 0,40 | 6,6E-06 | 4,4E-05 | 0,40 |
| *CTSW* | 7,1 | 8,4 | 5,7 | 5,9 | 6,8 | 4,5E-01 | 7,3E-01 | 0,84 | 4,6E-01 | 7,1E-01 | 1,24 | 4,5E-01 | 6,6E-01 | 1,21 | 8,7E-01 | 9,0E-01 | 1,03 |
| *PHF16* | 23,9 | 25,9 | 26,2 | 30,5 | 21,4 | 4,5E-01 | 7,3E-01 | 0,92 | 5,6E-01 | 8,0E-01 | 0,91 | 6,2E-02 | 1,5E-01 | 0,78 | 2,3E-01 | 3,4E-01 | 1,11 |
| *NDUFB8* | 1009,4 | 1079,2 | 1297,1 | 1357,3 | 1221,5 | 4,6E-01 | 7,4E-01 | 0,94 | 4,1E-02 | 1,3E-01 | 0,78 | 6,8E-03 | 2,9E-02 | 0,74 | 9,3E-03 | 2,4E-02 | 0,83 |
| *KCNA1* | 4,7 | 4,7 | 4,7 | 4,7 | 4,7 | 4,6E-01 | 7,4E-01 | 1,00 | 7,4E-01 | 8,7E-01 | 1,00 | 7,6E-01 | 8,6E-01 | 1,00 | 2,6E-01 | 3,8E-01 | 1,00 |
| *LAPTM5* | 723,6 | 870,7 | 202,4 | 227,4 | 463,4 | 4,6E-01 | 7,4E-01 | 0,83 | 2,3E-04 | 1,8E-03 | 3,58 | 5,0E-04 | 3,5E-03 | 3,18 | 5,4E-02 | 1,1E-01 | 1,56 |
| *EREG* | 6,0 | 7,3 | 4,8 | 4,8 | 4,5 | 4,6E-01 | 7,4E-01 | 0,82 | 4,2E-01 | 6,8E-01 | 1,26 | 3,8E-01 | 5,8E-01 | 1,25 | 9,5E-02 | 1,8E-01 | 1,32 |
| *TGM1* | 4,8 | 4,7 | 4,7 | 4,7 | 4,6 | 4,7E-01 | 7,4E-01 | 1,02 | 6,9E-01 | 8,5E-01 | 1,01 | 7,2E-01 | 8,5E-01 | 1,01 | 1,6E-01 | 2,6E-01 | 1,03 |
| *CIDEC* | 13,2 | 12,9 | 13,0 | 13,5 | 13,1 | 4,7E-01 | 7,4E-01 | 1,02 | 7,0E-01 | 8,5E-01 | 1,02 | 5,7E-01 | 7,5E-01 | 0,98 | 7,6E-01 | 8,2E-01 | 1,01 |
| *FAM184A* | 29,1 | 23,9 | 66,6 | 76,1 | 36,4 | 4,8E-01 | 7,4E-01 | 1,22 | 3,8E-02 | 1,2E-01 | 0,44 | 6,8E-03 | 2,9E-02 | 0,38 | 3,4E-01 | 4,6E-01 | 0,80 |
| *ADIPOQ* | 4,8 | 4,7 | 4,8 | 4,9 | 5,3 | 4,8E-01 | 7,4E-01 | 1,03 | 9,5E-01 | 9,7E-01 | 1,00 | 7,0E-01 | 8,5E-01 | 0,98 | 1,8E-01 | 2,9E-01 | 0,91 |
| *RUNX1* | 144,1 | 162,5 | 29,0 | 33,8 | 29,1 | 4,8E-01 | 7,4E-01 | 0,89 | 8,5E-10 | 4,1E-08 | 4,96 | 2,5E-09 | 1,9E-07 | 4,26 | 1,3E-16 | 7,0E-15 | 4,95 |
| *FASN* | 11,9 | 12,9 | 20,3 | 14,9 | 11,1 | 4,8E-01 | 7,4E-01 | 0,92 | 6,0E-03 | 2,7E-02 | 0,58 | 1,9E-01 | 3,7E-01 | 0,80 | 5,3E-01 | 6,4E-01 | 1,07 |
| *PAPPA* | 5,1 | 5,0 | 5,0 | 4,9 | 5,0 | 4,8E-01 | 7,4E-01 | 1,02 | 4,3E-01 | 6,8E-01 | 1,03 | 3,2E-01 | 5,2E-01 | 1,03 | 3,8E-01 | 4,9E-01 | 1,02 |
| *GBE1* | 184,8 | 197,8 | 151,4 | 194,8 | 234,5 | 4,8E-01 | 7,4E-01 | 0,93 | 1,7E-01 | 4,0E-01 | 1,22 | 6,8E-01 | 8,4E-01 | 0,95 | 6,7E-03 | 1,8E-02 | 0,79 |
| *ONECUT2* | 5,8 | 5,7 | 6,0 | 5,7 | 5,6 | 4,9E-01 | 7,4E-01 | 1,01 | 2,2E-01 | 4,5E-01 | 0,96 | 5,4E-01 | 7,4E-01 | 1,01 | 1,7E-01 | 2,8E-01 | 1,02 |
| *CSF2RB* | 14,4 | 18,5 | 6,6 | 7,0 | 12,5 | 4,9E-01 | 7,4E-01 | 0,78 | 7,4E-02 | 2,0E-01 | 2,20 | 5,7E-02 | 1,4E-01 | 2,06 | 6,3E-01 | 7,2E-01 | 1,16 |
| *AGPAT2* | 61,5 | 54,6 | 74,3 | 59,7 | 45,6 | 4,9E-01 | 7,4E-01 | 1,13 | 4,0E-01 | 6,6E-01 | 0,83 | 8,8E-01 | 9,2E-01 | 1,03 | 5,0E-02 | 1,1E-01 | 1,35 |
| *POU6F2* | 4,7 | 4,7 | 4,7 | 4,7 | 4,7 | 4,9E-01 | 7,4E-01 | 1,00 | 5,1E-01 | 7,6E-01 | 1,01 | 4,0E-01 | 6,1E-01 | 1,01 | 5,9E-01 | 6,9E-01 | 1,00 |
| *DLGAP1* | 4,8 | 4,7 | 4,6 | 6,1 | 4,8 | 4,9E-01 | 7,4E-01 | 1,02 | 3,4E-01 | 5,9E-01 | 1,03 | 3,2E-02 | 8,9E-02 | 0,78 | 6,9E-01 | 7,7E-01 | 0,99 |
| *KCNC2* | 4,7 | 4,7 | 4,7 | 4,7 | 4,7 | 4,9E-01 | 7,4E-01 | 0,99 | 8,4E-01 | 9,0E-01 | 1,00 | 9,2E-01 | 9,4E-01 | 1,00 | 1,9E-01 | 3,0E-01 | 1,01 |
| *WNT4* | 4,7 | 4,7 | 4,8 | 4,8 | 5,1 | 4,9E-01 | 7,4E-01 | 1,01 | 5,2E-02 | 1,5E-01 | 0,98 | 1,0E-02 | 3,8E-02 | 0,98 | 2,7E-02 | 6,5E-02 | 0,91 |
| *MAP3K13* | 15,8 | 15,2 | 13,1 | 16,3 | 13,1 | 5,0E-01 | 7,4E-01 | 1,04 | 5,4E-02 | 1,6E-01 | 1,20 | 6,8E-01 | 8,4E-01 | 0,97 | 3,0E-03 | 9,2E-03 | 1,20 |
| *HEPH* | 4,7 | 4,7 | 4,7 | 4,7 | 4,7 | 5,0E-01 | 7,4E-01 | 1,00 | 7,8E-01 | 8,7E-01 | 1,00 | 9,3E-01 | 9,5E-01 | 1,00 | 4,3E-01 | 5,4E-01 | 1,00 |
| *DCX* | 4,7 | 4,7 | 4,7 | 4,7 | 4,7 | 5,0E-01 | 7,4E-01 | 1,00 | 7,7E-01 | 8,7E-01 | 1,00 | 7,8E-01 | 8,8E-01 | 1,00 | 3,0E-01 | 4,2E-01 | 1,00 |
| *LRAT* | 4,7 | 4,7 | 4,7 | 4,7 | 4,6 | 5,0E-01 | 7,4E-01 | 1,02 | 7,8E-01 | 8,7E-01 | 1,01 | 8,0E-01 | 8,8E-01 | 1,01 | 3,6E-01 | 4,7E-01 | 1,02 |
| *PCDH11X* | 4,8 | 4,7 | 4,7 | 4,7 | 4,6 | 5,0E-01 | 7,4E-01 | 1,02 | 6,9E-01 | 8,5E-01 | 1,02 | 7,4E-01 | 8,6E-01 | 1,01 | 1,5E-01 | 2,5E-01 | 1,04 |
| *APOBEC2* | 6,9 | 6,2 | 6,7 | 6,3 | 5,4 | 5,0E-01 | 7,4E-01 | 1,11 | 9,2E-01 | 9,5E-01 | 1,02 | 6,4E-01 | 8,2E-01 | 1,10 | 9,6E-02 | 1,8E-01 | 1,26 |
| *ART3* | 4,9 | 4,7 | 4,7 | 4,8 | 4,6 | 5,0E-01 | 7,4E-01 | 1,05 | 7,7E-01 | 8,7E-01 | 1,03 | 8,0E-01 | 8,8E-01 | 1,03 | 3,6E-01 | 4,8E-01 | 1,06 |
| *ITGAM* | 37,9 | 45,0 | 9,6 | 13,5 | 20,4 | 5,1E-01 | 7,4E-01 | 0,84 | 1,6E-04 | 1,4E-03 | 3,94 | 1,1E-03 | 6,8E-03 | 2,81 | 4,7E-03 | 1,3E-02 | 1,86 |
| *AQP6* | 6,5 | 6,3 | 6,6 | 6,1 | 6,0 | 5,1E-01 | 7,4E-01 | 1,03 | 8,2E-01 | 8,9E-01 | 0,98 | 4,1E-01 | 6,1E-01 | 1,05 | 5,0E-02 | 1,1E-01 | 1,09 |
| *IL1RL1* | 36,9 | 26,4 | 8,6 | 4,4 | 11,1 | 5,1E-01 | 7,4E-01 | 1,39 | 4,1E-02 | 1,3E-01 | 4,26 | 3,5E-04 | 2,7E-03 | 8,43 | 3,8E-03 | 1,1E-02 | 3,33 |
| *KRT20* | 5,2 | 5,0 | 4,9 | 5,0 | 4,7 | 5,1E-01 | 7,4E-01 | 1,03 | 4,1E-01 | 6,7E-01 | 1,06 | 4,6E-01 | 6,7E-01 | 1,05 | 1,9E-02 | 4,6E-02 | 1,11 |
| *STYK1* | 5,1 | 5,0 | 5,6 | 5,1 | 4,8 | 5,1E-01 | 7,4E-01 | 1,03 | 1,1E-01 | 2,7E-01 | 0,91 | 8,9E-01 | 9,2E-01 | 1,01 | 3,2E-02 | 7,4E-02 | 1,07 |
| *KCNB1* | 4,9 | 4,7 | 4,8 | 4,9 | 4,7 | 5,1E-01 | 7,4E-01 | 1,03 | 7,5E-01 | 8,7E-01 | 1,02 | 9,1E-01 | 9,3E-01 | 1,01 | 3,6E-01 | 4,8E-01 | 1,04 |
| *SLC38A3* | 4,7 | 4,7 | 4,7 | 4,7 | 4,7 | 5,1E-01 | 7,4E-01 | 1,00 | 7,8E-01 | 8,7E-01 | 1,00 | 8,0E-01 | 8,8E-01 | 1,00 | 3,5E-01 | 4,6E-01 | 1,00 |
| *ARHGDIG* | 4,7 | 4,7 | 4,7 | 4,7 | 4,7 | 5,1E-01 | 7,4E-01 | 1,00 | 7,8E-01 | 8,7E-01 | 1,00 | 8,0E-01 | 8,8E-01 | 1,00 | 3,6E-01 | 4,7E-01 | 1,00 |
| *COX5A* | 1243,6 | 1187,5 | 1746,9 | 1370,9 | 1356,6 | 5,1E-01 | 7,4E-01 | 1,05 | 2,3E-03 | 1,3E-02 | 0,71 | 2,8E-01 | 4,8E-01 | 0,91 | 2,3E-01 | 3,5E-01 | 0,92 |
| *KCNB2* | 4,7 | 4,7 | 4,7 | 4,7 | 4,7 | 5,1E-01 | 7,4E-01 | 1,00 | 4,8E-01 | 7,3E-01 | 1,00 | 5,0E-01 | 6,9E-01 | 1,00 | 7,1E-01 | 7,8E-01 | 1,00 |
| *FMO1* | 4,8 | 5,0 | 4,9 | 5,0 | 5,4 | 5,1E-01 | 7,4E-01 | 0,97 | 7,6E-01 | 8,7E-01 | 0,99 | 4,1E-01 | 6,1E-01 | 0,97 | 1,8E-02 | 4,5E-02 | 0,89 |
| *RLN1* | 4,7 | 4,7 | 4,7 | 4,7 | 4,7 | 5,1E-01 | 7,4E-01 | 1,01 | 7,8E-01 | 8,7E-01 | 1,01 | 8,0E-01 | 8,8E-01 | 1,00 | 3,5E-01 | 4,7E-01 | 1,01 |
| *ABCD2* | 4,7 | 4,7 | 4,7 | 4,7 | 4,7 | 5,2E-01 | 7,4E-01 | 1,00 | 7,7E-01 | 8,7E-01 | 1,00 | 7,9E-01 | 8,8E-01 | 1,00 | 3,2E-01 | 4,4E-01 | 1,00 |
| *NEFL* | 4,9 | 4,7 | 8,7 | 4,6 | 4,8 | 5,2E-01 | 7,4E-01 | 1,04 | 1,8E-02 | 6,5E-02 | 0,56 | 3,8E-01 | 5,8E-01 | 1,07 | 7,4E-01 | 8,1E-01 | 1,02 |
| *PPP4R4* | 4,7 | 4,7 | 4,7 | 4,7 | 4,7 | 5,2E-01 | 7,4E-01 | 1,00 | 7,7E-01 | 8,7E-01 | 1,00 | 8,8E-01 | 9,2E-01 | 1,00 | 3,3E-01 | 4,5E-01 | 1,01 |
| *CYTL1* | 4,8 | 4,7 | 4,7 | 4,7 | 4,6 | 5,2E-01 | 7,5E-01 | 1,04 | 7,8E-01 | 8,7E-01 | 1,03 | 7,9E-01 | 8,8E-01 | 1,02 | 3,3E-01 | 4,5E-01 | 1,05 |
| *DTNA* | 4,8 | 4,9 | 6,6 | 7,9 | 5,0 | 5,2E-01 | 7,5E-01 | 0,98 | 1,3E-03 | 8,9E-03 | 0,73 | 8,0E-06 | 1,2E-04 | 0,61 | 2,3E-01 | 3,5E-01 | 0,97 |
| *IFI30* | 882,0 | 1024,8 | 700,8 | 479,1 | 529,6 | 5,2E-01 | 7,5E-01 | 0,86 | 4,3E-01 | 6,8E-01 | 1,26 | 2,6E-02 | 7,6E-02 | 1,84 | 1,2E-02 | 3,1E-02 | 1,67 |
| *SEC14L4* | 4,7 | 4,7 | 4,7 | 4,7 | 4,7 | 5,3E-01 | 7,5E-01 | 1,00 | 7,0E-01 | 8,5E-01 | 1,00 | 7,3E-01 | 8,6E-01 | 1,00 | 1,4E-01 | 2,5E-01 | 1,01 |
| *TGM3* | 4,9 | 4,8 | 4,8 | 4,8 | 4,8 | 5,3E-01 | 7,5E-01 | 1,01 | 7,5E-01 | 8,7E-01 | 1,01 | 7,8E-01 | 8,7E-01 | 1,01 | 2,7E-01 | 3,9E-01 | 1,02 |
| *AHR* | 365,0 | 398,0 | 96,6 | 148,7 | 202,3 | 5,3E-01 | 7,5E-01 | 0,92 | 3,1E-09 | 1,1E-07 | 3,78 | 9,2E-06 | 1,2E-04 | 2,45 | 2,4E-06 | 1,7E-05 | 1,80 |
| *SPCS3* | 8,5 | 7,8 | 11,1 | 9,1 | 12,3 | 5,3E-01 | 7,5E-01 | 1,09 | 2,0E-01 | 4,3E-01 | 0,77 | 7,0E-01 | 8,5E-01 | 0,94 | 4,4E-03 | 1,3E-02 | 0,69 |
| *ETV4* | 68,7 | 62,8 | 64,5 | 46,5 | 38,4 | 5,3E-01 | 7,5E-01 | 1,09 | 7,6E-01 | 8,7E-01 | 1,07 | 3,0E-02 | 8,4E-02 | 1,48 | 8,8E-06 | 5,5E-05 | 1,79 |
| *SLC5A5* | 14,7 | 14,3 | 14,6 | 15,5 | 18,2 | 5,3E-01 | 7,5E-01 | 1,03 | 9,0E-01 | 9,4E-01 | 1,01 | 1,8E-01 | 3,6E-01 | 0,95 | 7,7E-02 | 1,5E-01 | 0,81 |
| *THBS2* | 155,7 | 203,4 | 25,0 | 20,3 | 55,5 | 5,4E-01 | 7,6E-01 | 0,77 | 2,8E-03 | 1,5E-02 | 6,23 | 1,8E-04 | 1,6E-03 | 7,67 | 3,4E-03 | 1,0E-02 | 2,80 |
| *EHHADH* | 11,3 | 12,0 | 13,0 | 18,1 | 14,9 | 5,5E-01 | 7,6E-01 | 0,94 | 3,6E-01 | 6,2E-01 | 0,87 | 3,1E-03 | 1,5E-02 | 0,63 | 1,5E-03 | 5,1E-03 | 0,76 |
| *SCNN1B* | 5,9 | 6,2 | 10,4 | 6,4 | 9,8 | 5,5E-01 | 7,6E-01 | 0,95 | 1,1E-03 | 7,6E-03 | 0,57 | 3,3E-01 | 5,4E-01 | 0,92 | 2,7E-05 | 1,4E-04 | 0,60 |
| *SERPINB8* | 4,9 | 5,0 | 4,9 | 4,7 | 4,8 | 5,5E-01 | 7,7E-01 | 0,98 | 9,6E-01 | 9,7E-01 | 1,00 | 3,1E-01 | 5,2E-01 | 1,04 | 4,9E-01 | 6,0E-01 | 1,02 |
| *PHKG1* | 4,7 | 4,7 | 4,7 | 4,7 | 4,6 | 5,6E-01 | 7,7E-01 | 1,01 | 7,8E-01 | 8,7E-01 | 1,01 | 8,0E-01 | 8,8E-01 | 1,01 | 3,1E-01 | 4,3E-01 | 1,02 |
| *CD74* | 3635,0 | 3182,4 | 942,1 | 1041,6 | 2264,9 | 5,6E-01 | 7,8E-01 | 1,14 | 7,5E-05 | 6,9E-04 | 3,86 | 3,7E-05 | 4,0E-04 | 3,49 | 1,9E-02 | 4,5E-02 | 1,60 |
| *MAT1A* | 4,7 | 4,7 | 4,7 | 4,7 | 4,7 | 5,7E-01 | 7,8E-01 | 1,00 | 9,6E-01 | 9,7E-01 | 1,00 | 7,1E-01 | 8,5E-01 | 1,00 | 4,1E-01 | 5,2E-01 | 0,99 |
| *GPX3* | 4272,9 | 3742,8 | 5579,6 | 5863,9 | 7748,9 | 5,7E-01 | 7,8E-01 | 1,14 | 3,0E-01 | 5,4E-01 | 0,77 | 2,2E-01 | 4,1E-01 | 0,73 | 4,8E-04 | 1,8E-03 | 0,55 |
| *CCDC109B* | 88,1 | 101,5 | 52,0 | 15,4 | 43,8 | 5,7E-01 | 7,8E-01 | 0,87 | 1,4E-01 | 3,3E-01 | 1,70 | 2,0E-06 | 4,5E-05 | 5,71 | 3,2E-03 | 9,7E-03 | 2,01 |
| *GUCY1A2* | 4,7 | 4,7 | 4,7 | 4,7 | 4,7 | 5,7E-01 | 7,8E-01 | 1,00 | 7,1E-01 | 8,6E-01 | 1,00 | 6,8E-01 | 8,4E-01 | 1,00 | 4,3E-01 | 5,5E-01 | 1,00 |
| *TKT* | 1305,1 | 1403,8 | 1970,4 | 1990,1 | 1124,9 | 5,8E-01 | 7,9E-01 | 0,93 | 2,9E-02 | 9,4E-02 | 0,66 | 1,6E-02 | 5,7E-02 | 0,66 | 2,2E-01 | 3,3E-01 | 1,16 |
| *TBC1D8B* | 9,9 | 10,5 | 10,2 | 11,1 | 14,4 | 5,8E-01 | 7,9E-01 | 0,95 | 8,2E-01 | 8,9E-01 | 0,97 | 3,4E-01 | 5,4E-01 | 0,89 | 5,2E-04 | 2,0E-03 | 0,69 |
| *SNTG2* | 4,7 | 4,7 | 4,7 | 4,7 | 4,7 | 5,8E-01 | 7,9E-01 | 1,00 | 6,1E-01 | 8,3E-01 | 1,00 | 5,1E-01 | 7,1E-01 | 1,00 | 8,7E-01 | 9,0E-01 | 1,00 |
| *PDE7B* | 5,1 | 5,0 | 5,3 | 6,2 | 5,1 | 5,8E-01 | 7,9E-01 | 1,01 | 6,0E-02 | 1,7E-01 | 0,95 | 7,7E-03 | 3,1E-02 | 0,82 | 1,9E-01 | 3,1E-01 | 0,98 |
| *CLDN6* | 4,7 | 4,7 | 4,7 | 4,7 | 4,7 | 5,9E-01 | 8,0E-01 | 1,00 | 5,8E-01 | 8,1E-01 | 1,00 | 4,6E-01 | 6,7E-01 | 1,00 | 7,4E-01 | 8,1E-01 | 1,00 |
| *S100B* | 7,6 | 8,9 | 5,2 | 5,5 | 4,1 | 5,9E-01 | 8,0E-01 | 0,85 | 3,1E-01 | 5,5E-01 | 1,45 | 3,1E-01 | 5,1E-01 | 1,38 | 4,8E-03 | 1,4E-02 | 1,84 |
| *CD44* | 534,4 | 576,9 | 447,4 | 713,1 | 316,4 | 6,0E-01 | 8,0E-01 | 0,93 | 3,8E-01 | 6,4E-01 | 1,19 | 9,1E-02 | 2,0E-01 | 0,75 | 1,1E-05 | 6,3E-05 | 1,69 |
| *ALPK1* | 19,6 | 18,0 | 15,8 | 15,5 | 12,9 | 6,0E-01 | 8,0E-01 | 1,09 | 3,1E-01 | 5,5E-01 | 1,24 | 2,0E-01 | 3,8E-01 | 1,27 | 1,3E-03 | 4,4E-03 | 1,52 |
| *SLC14A2* | 4,7 | 4,7 | 4,7 | 4,7 | 4,7 | 6,0E-01 | 8,0E-01 | 1,00 | 8,0E-02 | 2,1E-01 | 0,99 | 6,3E-01 | 8,1E-01 | 1,00 | 5,8E-01 | 6,8E-01 | 1,00 |
| *ERO1LB* | 5,0 | 5,0 | 7,3 | 7,9 | 6,9 | 6,1E-01 | 8,1E-01 | 1,02 | 3,6E-05 | 3,7E-04 | 0,69 | 5,9E-04 | 4,0E-03 | 0,64 | 6,9E-06 | 4,6E-05 | 0,74 |
| *EXOC2* | 18,8 | 18,2 | 19,1 | 22,9 | 23,4 | 6,1E-01 | 8,1E-01 | 1,04 | 8,9E-01 | 9,3E-01 | 0,99 | 4,1E-02 | 1,0E-01 | 0,82 | 1,4E-03 | 4,7E-03 | 0,80 |
| *MPP6* | 5,1 | 5,2 | 5,5 | 6,5 | 6,2 | 6,1E-01 | 8,1E-01 | 0,98 | 8,4E-02 | 2,2E-01 | 0,92 | 2,6E-02 | 7,7E-02 | 0,78 | 9,3E-06 | 5,7E-05 | 0,82 |
| *GPRC5A* | 695,0 | 626,8 | 255,4 | 416,9 | 336,9 | 6,1E-01 | 8,1E-01 | 1,11 | 3,4E-05 | 3,7E-04 | 2,72 | 8,2E-03 | 3,2E-02 | 1,67 | 7,8E-06 | 5,1E-05 | 2,06 |
| *PNPLA3* | 4,7 | 4,7 | 4,7 | 4,7 | 4,7 | 6,1E-01 | 8,1E-01 | 1,00 | 7,7E-01 | 8,7E-01 | 1,00 | 8,4E-01 | 9,0E-01 | 1,00 | 1,4E-01 | 2,4E-01 | 1,00 |
| *DRAM1* | 213,5 | 234,8 | 50,6 | 78,1 | 77,7 | 6,1E-01 | 8,1E-01 | 0,91 | 2,8E-07 | 5,3E-06 | 4,22 | 3,2E-05 | 3,5E-04 | 2,73 | 5,6E-09 | 6,9E-08 | 2,75 |
| *IVL* | 6,0 | 6,6 | 4,5 | 4,5 | 4,9 | 6,2E-01 | 8,1E-01 | 0,91 | 2,6E-01 | 4,9E-01 | 1,33 | 1,7E-01 | 3,4E-01 | 1,34 | 1,4E-01 | 2,5E-01 | 1,23 |
| *GREM1* | 7,6 | 8,9 | 5,2 | 5,2 | 6,3 | 6,2E-01 | 8,1E-01 | 0,85 | 3,6E-01 | 6,2E-01 | 1,45 | 2,8E-01 | 4,8E-01 | 1,46 | 4,6E-01 | 5,7E-01 | 1,21 |
| *PGLYRP1* | 4,7 | 4,7 | 4,7 | 4,7 | 4,7 | 6,2E-01 | 8,1E-01 | 1,00 | 6,0E-01 | 8,3E-01 | 1,00 | 4,9E-01 | 6,9E-01 | 1,00 | 7,5E-01 | 8,2E-01 | 1,00 |
| *LBP* | 4,8 | 4,7 | 4,8 | 4,7 | 4,7 | 6,2E-01 | 8,1E-01 | 1,01 | 7,8E-01 | 8,7E-01 | 1,01 | 6,5E-01 | 8,3E-01 | 1,01 | 1,0E-01 | 1,9E-01 | 1,03 |
| *CDKL1* | 4,7 | 4,7 | 4,7 | 4,7 | 4,7 | 6,2E-01 | 8,1E-01 | 1,00 | 7,8E-01 | 8,7E-01 | 1,00 | 8,1E-01 | 8,8E-01 | 1,00 | 3,1E-01 | 4,3E-01 | 1,01 |
| *ACSS3* | 16,1 | 15,2 | 18,4 | 16,9 | 15,0 | 6,2E-01 | 8,1E-01 | 1,06 | 4,5E-01 | 7,1E-01 | 0,87 | 7,6E-01 | 8,6E-01 | 0,95 | 4,0E-01 | 5,2E-01 | 1,08 |
| *ALOX5AP* | 181,7 | 205,3 | 28,6 | 36,6 | 69,5 | 6,3E-01 | 8,2E-01 | 0,89 | 6,9E-07 | 1,2E-05 | 6,35 | 2,9E-06 | 5,8E-05 | 4,96 | 7,9E-05 | 3,5E-04 | 2,62 |
| *LILRB4* | 7,6 | 8,2 | 6,5 | 5,7 | 6,0 | 6,3E-01 | 8,2E-01 | 0,93 | 4,2E-01 | 6,7E-01 | 1,17 | 9,1E-02 | 2,0E-01 | 1,32 | 3,4E-02 | 7,8E-02 | 1,27 |
| *OTUB2* | 4,8 | 4,8 | 5,0 | 4,8 | 4,7 | 6,4E-01 | 8,2E-01 | 1,01 | 4,0E-01 | 6,6E-01 | 0,97 | 6,7E-01 | 8,4E-01 | 1,01 | 3,3E-01 | 4,5E-01 | 1,03 |
| *IL18R1* | 5,9 | 5,5 | 9,7 | 5,1 | 5,3 | 6,4E-01 | 8,2E-01 | 1,07 | 1,4E-01 | 3,4E-01 | 0,61 | 3,5E-01 | 5,4E-01 | 1,18 | 3,1E-01 | 4,3E-01 | 1,12 |
| *CIITA* | 11,3 | 10,5 | 8,7 | 8,2 | 9,0 | 6,4E-01 | 8,2E-01 | 1,08 | 1,9E-01 | 4,2E-01 | 1,30 | 6,4E-02 | 1,5E-01 | 1,37 | 8,3E-02 | 1,6E-01 | 1,26 |
| *PRKAR2B* | 7,5 | 8,3 | 7,5 | 5,9 | 10,7 | 6,4E-01 | 8,2E-01 | 0,91 | 9,9E-01 | 9,9E-01 | 1,00 | 2,7E-01 | 4,7E-01 | 1,29 | 3,7E-02 | 8,4E-02 | 0,71 |
| *CXCL12* | 52,8 | 60,0 | 22,2 | 32,2 | 65,1 | 6,4E-01 | 8,2E-01 | 0,88 | 2,5E-02 | 8,6E-02 | 2,38 | 1,3E-01 | 2,7E-01 | 1,64 | 3,6E-01 | 4,8E-01 | 0,81 |
| *CLU* | 13407,4 | 14678,2 | 2562,7 | 3352,1 | 5784,1 | 6,5E-01 | 8,2E-01 | 0,91 | 1,9E-08 | 5,1E-07 | 5,23 | 2,3E-06 | 4,8E-05 | 4,00 | 2,5E-07 | 2,2E-06 | 2,32 |
| *DNA2* | 4,7 | 4,7 | 5,3 | 4,7 | 4,7 | 6,5E-01 | 8,2E-01 | 1,00 | 6,6E-04 | 4,9E-03 | 0,88 | 8,1E-01 | 8,8E-01 | 1,00 | 1,1E-01 | 2,1E-01 | 0,99 |
| *RPRM* | 4,7 | 4,7 | 5,4 | 5,3 | 4,8 | 6,5E-01 | 8,3E-01 | 1,01 | 1,7E-03 | 1,1E-02 | 0,87 | 1,6E-02 | 5,7E-02 | 0,90 | 4,5E-01 | 5,5E-01 | 0,99 |
| *UCP3* | 4,8 | 4,7 | 4,7 | 4,7 | 4,7 | 6,5E-01 | 8,3E-01 | 1,02 | 5,6E-01 | 8,0E-01 | 1,04 | 4,8E-01 | 6,8E-01 | 1,04 | 4,0E-01 | 5,2E-01 | 1,03 |
| *ITGA4* | 6,9 | 6,3 | 4,6 | 4,6 | 5,9 | 6,6E-01 | 8,3E-01 | 1,10 | 1,8E-01 | 4,0E-01 | 1,49 | 1,2E-01 | 2,5E-01 | 1,49 | 4,4E-01 | 5,5E-01 | 1,16 |
| *MEG3* | 4,8 | 4,7 | 4,6 | 4,6 | 4,7 | 6,7E-01 | 8,3E-01 | 1,01 | 6,1E-01 | 8,3E-01 | 1,02 | 5,0E-01 | 7,0E-01 | 1,03 | 7,3E-01 | 8,0E-01 | 1,01 |
| *FCGR2B* | 25,8 | 29,5 | 10,5 | 11,9 | 23,1 | 6,7E-01 | 8,3E-01 | 0,88 | 2,2E-02 | 7,7E-02 | 2,45 | 2,2E-02 | 6,7E-02 | 2,18 | 6,6E-01 | 7,4E-01 | 1,12 |
| *PRR5L* | 4,8 | 4,9 | 4,7 | 4,7 | 4,7 | 6,7E-01 | 8,3E-01 | 0,98 | 6,6E-01 | 8,5E-01 | 1,02 | 7,1E-01 | 8,5E-01 | 1,01 | 5,6E-01 | 6,7E-01 | 1,02 |
| *TAC1* | 4,9 | 4,7 | 4,7 | 4,7 | 5,2 | 6,7E-01 | 8,3E-01 | 1,04 | 7,0E-01 | 8,5E-01 | 1,05 | 6,5E-01 | 8,3E-01 | 1,05 | 5,4E-01 | 6,4E-01 | 0,94 |
| *TRPA1* | 4,7 | 4,7 | 4,7 | 4,7 | 4,7 | 6,7E-01 | 8,3E-01 | 1,00 | 7,7E-01 | 8,7E-01 | 1,00 | 7,7E-01 | 8,7E-01 | 1,00 | 3,4E-01 | 4,6E-01 | 1,00 |
| *HMGA2* | 177,5 | 213,0 | 79,9 | 53,1 | 10,6 | 6,8E-01 | 8,3E-01 | 0,83 | 1,7E-01 | 3,9E-01 | 2,22 | 3,6E-02 | 9,6E-02 | 3,34 | 1,6E-12 | 3,3E-11 | 16,74 |
| *ICAM1* | 400,8 | 434,0 | 107,4 | 107,4 | 107,0 | 6,8E-01 | 8,3E-01 | 0,92 | 4,0E-07 | 7,4E-06 | 3,73 | 5,2E-07 | 1,5E-05 | 3,73 | 4,1E-12 | 8,3E-11 | 3,75 |
| *ATP5L* | 533,0 | 513,8 | 590,2 | 676,5 | 685,8 | 6,8E-01 | 8,3E-01 | 1,04 | 4,1E-01 | 6,6E-01 | 0,90 | 3,5E-02 | 9,6E-02 | 0,79 | 6,2E-04 | 2,3E-03 | 0,78 |
| *NTF3* | 4,9 | 4,8 | 4,7 | 4,8 | 4,6 | 6,8E-01 | 8,3E-01 | 1,03 | 7,3E-01 | 8,7E-01 | 1,03 | 7,5E-01 | 8,6E-01 | 1,03 | 3,6E-01 | 4,8E-01 | 1,05 |
| *IL1RL2* | 5,2 | 5,1 | 5,4 | 4,9 | 4,8 | 6,8E-01 | 8,4E-01 | 1,02 | 6,9E-01 | 8,5E-01 | 0,97 | 2,3E-01 | 4,2E-01 | 1,07 | 3,7E-02 | 8,4E-02 | 1,09 |
| *LGR5* | 4,7 | 4,7 | 4,7 | 4,7 | 4,7 | 6,9E-01 | 8,4E-01 | 1,00 | 4,7E-01 | 7,2E-01 | 1,01 | 3,4E-01 | 5,4E-01 | 1,01 | 6,7E-01 | 7,5E-01 | 1,00 |
| *WISP2* | 6,2 | 6,9 | 5,3 | 5,6 | 4,5 | 6,9E-01 | 8,4E-01 | 0,90 | 6,3E-01 | 8,4E-01 | 1,18 | 7,1E-01 | 8,5E-01 | 1,11 | 1,1E-01 | 2,0E-01 | 1,38 |
| *GDF10* | 5,6 | 5,4 | 6,2 | 10,4 | 12,8 | 7,0E-01 | 8,5E-01 | 1,03 | 2,2E-01 | 4,5E-01 | 0,90 | 6,2E-03 | 2,8E-02 | 0,54 | 2,8E-10 | 4,3E-09 | 0,44 |
| *LILRA6* | 4,8 | 4,7 | 4,7 | 4,7 | 4,7 | 7,0E-01 | 8,5E-01 | 1,01 | 6,6E-01 | 8,5E-01 | 1,01 | 7,2E-01 | 8,5E-01 | 1,01 | 2,6E-01 | 3,8E-01 | 1,02 |
| *CISD1* | 155,8 | 165,2 | 194,3 | 122,6 | 192,6 | 7,0E-01 | 8,5E-01 | 0,94 | 3,1E-01 | 5,5E-01 | 0,80 | 2,1E-01 | 3,9E-01 | 1,27 | 1,1E-01 | 2,1E-01 | 0,81 |
| *MMP13* | 5,3 | 5,7 | 4,7 | 4,7 | 4,6 | 7,0E-01 | 8,5E-01 | 0,93 | 5,9E-01 | 8,2E-01 | 1,12 | 5,5E-01 | 7,4E-01 | 1,12 | 2,7E-01 | 3,9E-01 | 1,15 |
| *KLF5* | 120,3 | 111,0 | 94,3 | 55,3 | 61,0 | 7,0E-01 | 8,5E-01 | 1,08 | 3,6E-01 | 6,2E-01 | 1,27 | 1,9E-03 | 1,1E-02 | 2,18 | 2,7E-04 | 1,1E-03 | 1,97 |
| *PDGFD* | 60,6 | 68,0 | 149,5 | 216,0 | 188,6 | 7,1E-01 | 8,5E-01 | 0,89 | 2,6E-02 | 8,6E-02 | 0,41 | 3,7E-04 | 2,8E-03 | 0,28 | 3,1E-06 | 2,1E-05 | 0,32 |
| *BMP7* | 146,4 | 157,6 | 172,0 | 248,9 | 301,3 | 7,1E-01 | 8,5E-01 | 0,93 | 3,7E-01 | 6,2E-01 | 0,85 | 9,6E-04 | 5,8E-03 | 0,59 | 1,4E-07 | 1,3E-06 | 0,49 |
| *NCF4* | 15,4 | 16,8 | 7,2 | 7,3 | 12,9 | 7,1E-01 | 8,5E-01 | 0,91 | 1,8E-02 | 6,6E-02 | 2,12 | 7,6E-03 | 3,1E-02 | 2,10 | 4,1E-01 | 5,2E-01 | 1,19 |
| *EDIL3* | 4,8 | 4,7 | 4,6 | 4,7 | 4,7 | 7,1E-01 | 8,5E-01 | 1,01 | 5,1E-01 | 7,6E-01 | 1,04 | 6,1E-01 | 7,9E-01 | 1,02 | 7,0E-01 | 7,8E-01 | 1,01 |
| *LPHN3* | 5,1 | 4,9 | 4,6 | 4,5 | 4,9 | 7,2E-01 | 8,6E-01 | 1,06 | 6,0E-01 | 8,3E-01 | 1,13 | 5,1E-01 | 7,1E-01 | 1,14 | 7,4E-01 | 8,1E-01 | 1,05 |
| *IFI44* | 72,9 | 80,4 | 34,2 | 29,8 | 65,1 | 7,2E-01 | 8,6E-01 | 0,91 | 5,1E-02 | 1,5E-01 | 2,13 | 9,8E-03 | 3,7E-02 | 2,45 | 6,4E-01 | 7,2E-01 | 1,12 |
| *SEC14L5* | 4,7 | 4,7 | 4,7 | 4,7 | 4,7 | 7,2E-01 | 8,6E-01 | 1,00 | 6,7E-01 | 8,5E-01 | 1,01 | 7,1E-01 | 8,5E-01 | 1,00 | 2,1E-01 | 3,2E-01 | 1,01 |
| *NCAM1* | 27,8 | 24,8 | 36,0 | 108,9 | 188,5 | 7,2E-01 | 8,6E-01 | 1,12 | 5,6E-01 | 8,0E-01 | 0,77 | 2,0E-03 | 1,1E-02 | 0,26 | 2,9E-10 | 4,4E-09 | 0,15 |
| *SNTG1* | 4,7 | 4,7 | 4,7 | 4,7 | 4,7 | 7,2E-01 | 8,6E-01 | 1,00 | 1,2E-01 | 3,0E-01 | 1,00 | 4,0E-01 | 6,1E-01 | 1,00 | 9,1E-01 | 9,2E-01 | 1,00 |
| *RAB17* | 88,3 | 91,3 | 172,5 | 178,0 | 146,4 | 7,2E-01 | 8,6E-01 | 0,97 | 5,2E-08 | 1,2E-06 | 0,51 | 1,3E-08 | 8,8E-07 | 0,50 | 1,5E-07 | 1,3E-06 | 0,60 |
| *FOXF2* | 5,8 | 5,3 | 5,0 | 9,4 | 9,7 | 7,2E-01 | 8,6E-01 | 1,09 | 7,1E-01 | 8,6E-01 | 1,15 | 1,9E-01 | 3,6E-01 | 0,61 | 3,0E-02 | 7,1E-02 | 0,59 |
| *PRRG3* | 4,7 | 4,7 | 4,7 | 4,7 | 4,7 | 7,3E-01 | 8,6E-01 | 1,00 | 6,3E-01 | 8,5E-01 | 1,00 | 5,4E-01 | 7,3E-01 | 1,01 | 7,5E-01 | 8,2E-01 | 1,00 |
| *SCEL* | 134,8 | 120,4 | 5,0 | 17,6 | 10,5 | 7,3E-01 | 8,6E-01 | 1,12 | 6,1E-09 | 2,0E-07 | 26,86 | 5,5E-05 | 5,8E-04 | 7,66 | 6,5E-15 | 2,5E-13 | 12,89 |
| *DGKB* | 4,7 | 4,7 | 4,7 | 4,7 | 4,7 | 7,3E-01 | 8,6E-01 | 1,00 | 1,9E-02 | 6,9E-02 | 1,00 | 4,3E-01 | 6,3E-01 | 1,00 | 9,2E-01 | 9,3E-01 | 1,00 |
| *ARL5A* | 339,2 | 347,4 | 528,4 | 539,0 | 469,0 | 7,3E-01 | 8,6E-01 | 0,98 | 6,1E-05 | 5,7E-04 | 0,64 | 6,6E-06 | 1,1E-04 | 0,63 | 1,4E-06 | 9,7E-06 | 0,72 |
| *CORO2A* | 5,8 | 5,7 | 6,6 | 6,0 | 5,1 | 7,4E-01 | 8,6E-01 | 1,03 | 4,3E-01 | 6,8E-01 | 0,89 | 8,7E-01 | 9,1E-01 | 0,98 | 4,0E-02 | 9,0E-02 | 1,15 |
| *CCND1* | 185,7 | 199,6 | 141,4 | 121,8 | 67,8 | 7,4E-01 | 8,6E-01 | 0,93 | 3,5E-01 | 6,0E-01 | 1,31 | 8,8E-02 | 1,9E-01 | 1,52 | 1,1E-07 | 1,0E-06 | 2,74 |
| *TPPP* | 5,2 | 5,1 | 5,8 | 6,7 | 10,6 | 7,4E-01 | 8,6E-01 | 1,02 | 1,7E-01 | 4,0E-01 | 0,90 | 6,1E-04 | 4,1E-03 | 0,78 | 4,1E-07 | 3,2E-06 | 0,50 |
| *MYCN* | 5,4 | 5,3 | 5,1 | 6,3 | 6,5 | 7,4E-01 | 8,6E-01 | 1,03 | 6,4E-01 | 8,5E-01 | 1,06 | 2,9E-01 | 4,9E-01 | 0,86 | 4,5E-02 | 9,8E-02 | 0,84 |
| *RGS20* | 4,7 | 4,7 | 4,7 | 4,8 | 4,7 | 7,4E-01 | 8,6E-01 | 1,00 | 5,7E-01 | 8,0E-01 | 1,00 | 4,1E-02 | 1,1E-01 | 0,98 | 1,7E-01 | 2,8E-01 | 1,00 |
| *GJB4* | 4,7 | 4,7 | 4,7 | 4,7 | 4,7 | 7,5E-01 | 8,6E-01 | 1,00 | 8,4E-01 | 9,0E-01 | 1,00 | 7,4E-01 | 8,6E-01 | 1,00 | 2,2E-01 | 3,3E-01 | 1,01 |
| *SPP1* | 127,9 | 146,4 | 20,5 | 59,7 | 43,2 | 7,5E-01 | 8,6E-01 | 0,87 | 1,7E-03 | 1,1E-02 | 6,23 | 2,0E-01 | 3,7E-01 | 2,14 | 3,6E-03 | 1,1E-02 | 2,96 |
| *LUZP2* | 4,8 | 4,8 | 4,8 | 4,8 | 4,8 | 7,5E-01 | 8,6E-01 | 1,00 | 7,4E-01 | 8,7E-01 | 1,00 | 6,8E-01 | 8,4E-01 | 1,00 | 7,4E-01 | 8,1E-01 | 1,00 |
| *PENK* | 4,9 | 4,9 | 4,8 | 4,8 | 4,7 | 7,5E-01 | 8,6E-01 | 1,01 | 5,5E-01 | 8,0E-01 | 1,02 | 5,6E-01 | 7,4E-01 | 1,02 | 8,0E-02 | 1,6E-01 | 1,04 |
| *INHBB* | 270,7 | 300,7 | 37,4 | 57,4 | 78,6 | 7,5E-01 | 8,6E-01 | 0,90 | 5,4E-05 | 5,4E-04 | 7,23 | 6,3E-04 | 4,1E-03 | 4,72 | 8,3E-06 | 5,3E-05 | 3,44 |
| *CASP4* | 6,7 | 6,4 | 5,5 | 5,5 | 6,4 | 7,5E-01 | 8,6E-01 | 1,04 | 2,4E-01 | 4,6E-01 | 1,21 | 1,5E-01 | 3,0E-01 | 1,22 | 7,9E-01 | 8,5E-01 | 1,03 |
| *SFRP5* | 4,8 | 4,9 | 4,7 | 4,8 | 4,6 | 7,6E-01 | 8,6E-01 | 0,99 | 6,6E-01 | 8,5E-01 | 1,02 | 7,5E-01 | 8,6E-01 | 1,01 | 4,0E-02 | 9,0E-02 | 1,05 |
| *XYLB* | 4,7 | 4,7 | 4,7 | 4,7 | 4,8 | 7,6E-01 | 8,6E-01 | 1,01 | 6,7E-01 | 8,5E-01 | 1,01 | 6,1E-01 | 7,9E-01 | 1,02 | 7,8E-01 | 8,4E-01 | 0,99 |
| *PRR16* | 4,7 | 4,7 | 4,7 | 4,8 | 4,7 | 7,6E-01 | 8,7E-01 | 1,00 | 9,3E-01 | 9,6E-01 | 1,00 | 5,8E-01 | 7,7E-01 | 0,99 | 5,1E-01 | 6,2E-01 | 1,01 |
| *CCL17* | 5,9 | 5,6 | 4,8 | 4,9 | 4,7 | 7,7E-01 | 8,7E-01 | 1,05 | 3,9E-01 | 6,5E-01 | 1,22 | 3,4E-01 | 5,4E-01 | 1,21 | 8,2E-02 | 1,6E-01 | 1,27 |
| *CD80* | 5,4 | 5,4 | 5,3 | 5,3 | 5,2 | 7,7E-01 | 8,7E-01 | 0,99 | 6,2E-01 | 8,4E-01 | 1,02 | 6,8E-01 | 8,4E-01 | 1,02 | 7,9E-02 | 1,6E-01 | 1,04 |
| *COL5A2* | 256,4 | 279,6 | 151,3 | 89,0 | 107,0 | 7,7E-01 | 8,7E-01 | 0,92 | 1,8E-01 | 4,0E-01 | 1,69 | 4,0E-03 | 1,9E-02 | 2,88 | 3,1E-04 | 1,2E-03 | 2,40 |
| *MALT1* | 26,3 | 27,2 | 17,2 | 20,1 | 25,6 | 7,8E-01 | 8,8E-01 | 0,97 | 4,7E-03 | 2,2E-02 | 1,53 | 3,8E-02 | 9,9E-02 | 1,31 | 8,2E-01 | 8,7E-01 | 1,03 |
| *PTPN2* | 4,7 | 4,7 | 4,8 | 5,3 | 4,8 | 7,8E-01 | 8,8E-01 | 1,00 | 4,5E-01 | 6,9E-01 | 0,99 | 3,0E-04 | 2,3E-03 | 0,90 | 1,1E-01 | 2,0E-01 | 0,98 |
| *ALDOC* | 10,5 | 11,0 | 12,1 | 7,1 | 10,2 | 7,8E-01 | 8,8E-01 | 0,96 | 6,4E-01 | 8,5E-01 | 0,87 | 4,0E-02 | 1,0E-01 | 1,49 | 8,0E-01 | 8,5E-01 | 1,04 |
| *SOCS3* | 18,8 | 19,2 | 17,9 | 15,4 | 17,5 | 7,9E-01 | 8,9E-01 | 0,98 | 6,6E-01 | 8,5E-01 | 1,05 | 2,7E-02 | 7,7E-02 | 1,22 | 4,3E-01 | 5,4E-01 | 1,07 |
| *RAI2* | 140,9 | 133,4 | 62,1 | 90,4 | 130,6 | 8,0E-01 | 8,9E-01 | 1,06 | 1,3E-02 | 5,0E-02 | 2,27 | 8,4E-02 | 1,8E-01 | 1,56 | 6,6E-01 | 7,4E-01 | 1,08 |
| *NPC2* | 10007,5 | 9792,6 | 6239,1 | 7040,5 | 3872,3 | 8,1E-01 | 9,0E-01 | 1,02 | 6,8E-04 | 4,9E-03 | 1,60 | 9,3E-03 | 3,6E-02 | 1,42 | 4,1E-18 | 2,8E-16 | 2,58 |
| *SPTLC3* | 5,0 | 4,9 | 5,1 | 5,6 | 5,0 | 8,1E-01 | 9,0E-01 | 1,01 | 6,7E-01 | 8,5E-01 | 0,98 | 7,6E-02 | 1,7E-01 | 0,89 | 8,2E-01 | 8,7E-01 | 0,99 |
| *THEMIS2* | 123,5 | 128,0 | 25,0 | 46,9 | 70,1 | 8,1E-01 | 9,0E-01 | 0,97 | 2,8E-09 | 1,1E-07 | 4,94 | 3,1E-05 | 3,5E-04 | 2,63 | 4,6E-04 | 1,8E-03 | 1,76 |
| *IL1RN* | 56,0 | 51,6 | 7,1 | 13,6 | 14,1 | 8,2E-01 | 9,0E-01 | 1,08 | 1,0E-05 | 1,3E-04 | 7,86 | 4,9E-04 | 3,5E-03 | 4,12 | 6,2E-07 | 4,6E-06 | 3,96 |
| *CXCL11* | 10,1 | 11,2 | 5,6 | 6,0 | 10,6 | 8,2E-01 | 9,0E-01 | 0,90 | 2,6E-01 | 4,9E-01 | 1,81 | 2,4E-01 | 4,3E-01 | 1,70 | 9,0E-01 | 9,2E-01 | 0,96 |
| *LRP3* | 9,6 | 9,4 | 9,7 | 10,3 | 8,9 | 8,2E-01 | 9,0E-01 | 1,02 | 9,2E-01 | 9,5E-01 | 0,99 | 5,5E-01 | 7,4E-01 | 0,94 | 4,3E-01 | 5,4E-01 | 1,07 |
| *FAM49A* | 172,9 | 177,2 | 206,4 | 331,0 | 270,5 | 8,2E-01 | 9,0E-01 | 0,98 | 1,9E-01 | 4,2E-01 | 0,84 | 6,0E-08 | 2,7E-06 | 0,52 | 3,0E-07 | 2,5E-06 | 0,64 |
| *EGLN3* | 5,6 | 5,8 | 5,6 | 5,1 | 5,2 | 8,2E-01 | 9,0E-01 | 0,96 | 9,7E-01 | 9,7E-01 | 0,99 | 6,7E-01 | 8,4E-01 | 1,09 | 5,2E-01 | 6,3E-01 | 1,08 |
| *MGLL* | 30,6 | 32,1 | 21,0 | 27,0 | 32,0 | 8,2E-01 | 9,0E-01 | 0,95 | 2,3E-01 | 4,5E-01 | 1,45 | 6,8E-01 | 8,4E-01 | 1,13 | 8,0E-01 | 8,6E-01 | 0,96 |
| *CCR2* | 11,8 | 11,1 | 6,5 | 6,7 | 11,3 | 8,2E-01 | 9,0E-01 | 1,06 | 1,1E-01 | 2,7E-01 | 1,82 | 8,1E-02 | 1,8E-01 | 1,75 | 8,6E-01 | 9,0E-01 | 1,05 |
| *PLEK2* | 103,6 | 106,6 | 181,5 | 203,1 | 143,0 | 8,3E-01 | 9,0E-01 | 0,97 | 1,9E-03 | 1,1E-02 | 0,57 | 1,5E-05 | 2,0E-04 | 0,51 | 3,1E-03 | 9,5E-03 | 0,72 |
| *RETN* | 5,0 | 5,1 | 4,6 | 4,6 | 4,8 | 8,3E-01 | 9,0E-01 | 0,97 | 6,5E-01 | 8,5E-01 | 1,08 | 5,8E-01 | 7,6E-01 | 1,08 | 6,6E-01 | 7,4E-01 | 1,04 |
| *TMEM35* | 4,7 | 4,7 | 4,6 | 5,6 | 4,8 | 8,3E-01 | 9,0E-01 | 1,00 | 4,3E-01 | 6,8E-01 | 1,02 | 5,8E-02 | 1,4E-01 | 0,84 | 2,2E-01 | 3,3E-01 | 0,98 |
| *CD53* | 296,9 | 315,8 | 48,0 | 67,9 | 207,9 | 8,4E-01 | 9,1E-01 | 0,94 | 2,7E-05 | 2,9E-04 | 6,18 | 1,3E-04 | 1,2E-03 | 4,37 | 2,0E-01 | 3,2E-01 | 1,43 |
| *PTAFR* | 23,3 | 23,6 | 21,5 | 19,4 | 19,9 | 8,4E-01 | 9,1E-01 | 0,99 | 1,8E-01 | 4,0E-01 | 1,09 | 2,1E-03 | 1,1E-02 | 1,20 | 1,4E-04 | 6,0E-04 | 1,17 |
| *PADI2* | 6,5 | 6,3 | 5,7 | 5,8 | 4,9 | 8,4E-01 | 9,1E-01 | 1,03 | 5,7E-01 | 8,0E-01 | 1,13 | 5,1E-01 | 7,1E-01 | 1,13 | 3,0E-02 | 7,0E-02 | 1,31 |
| *ADAMTS8* | 4,9 | 4,8 | 4,8 | 4,8 | 4,8 | 8,4E-01 | 9,1E-01 | 1,00 | 6,3E-01 | 8,4E-01 | 1,01 | 4,2E-01 | 6,2E-01 | 1,01 | 4,9E-02 | 1,1E-01 | 1,02 |
| *LARP6* | 107,8 | 102,6 | 77,2 | 107,0 | 131,2 | 8,5E-01 | 9,1E-01 | 1,05 | 3,4E-01 | 5,9E-01 | 1,40 | 9,8E-01 | 9,8E-01 | 1,01 | 3,2E-01 | 4,4E-01 | 0,82 |
| *BMP6* | 4,9 | 4,9 | 4,9 | 4,9 | 5,2 | 8,5E-01 | 9,1E-01 | 1,01 | 7,9E-01 | 8,7E-01 | 1,01 | 8,4E-01 | 9,0E-01 | 1,01 | 2,6E-01 | 3,9E-01 | 0,95 |
| *MEIS2* | 10,7 | 10,4 | 8,1 | 18,9 | 25,1 | 8,6E-01 | 9,2E-01 | 1,03 | 2,2E-01 | 4,4E-01 | 1,31 | 2,1E-02 | 6,6E-02 | 0,56 | 1,5E-07 | 1,3E-06 | 0,42 |
| *RGS4* | 37,0 | 34,1 | 5,3 | 4,4 | 11,6 | 8,6E-01 | 9,2E-01 | 1,09 | 3,8E-03 | 1,9E-02 | 7,00 | 3,2E-04 | 2,5E-03 | 8,46 | 3,3E-03 | 9,8E-03 | 3,17 |
| *ASS1* | 944,5 | 926,8 | 783,0 | 624,2 | 756,9 | 8,7E-01 | 9,3E-01 | 1,02 | 2,3E-01 | 4,5E-01 | 1,21 | 1,9E-02 | 6,0E-02 | 1,51 | 3,7E-02 | 8,3E-02 | 1,25 |
| *CDK6* | 4,7 | 4,7 | 4,7 | 4,7 | 4,7 | 8,7E-01 | 9,3E-01 | 1,00 | 5,0E-01 | 7,4E-01 | 1,01 | 3,9E-01 | 5,9E-01 | 1,01 | 7,7E-01 | 8,3E-01 | 1,00 |
| *CNR1* | 5,1 | 5,0 | 5,3 | 4,4 | 5,0 | 8,7E-01 | 9,3E-01 | 1,02 | 8,2E-01 | 8,9E-01 | 0,95 | 3,9E-01 | 6,0E-01 | 1,17 | 8,9E-01 | 9,2E-01 | 1,02 |
| *PLAGL1* | 5,9 | 5,9 | 5,4 | 5,8 | 6,4 | 8,9E-01 | 9,4E-01 | 1,01 | 4,3E-01 | 6,8E-01 | 1,10 | 8,3E-01 | 8,9E-01 | 1,02 | 3,9E-01 | 5,0E-01 | 0,93 |
| *PPARG* | 6,0 | 5,9 | 5,9 | 7,7 | 11,5 | 8,9E-01 | 9,4E-01 | 1,01 | 9,2E-01 | 9,5E-01 | 1,01 | 5,4E-02 | 1,3E-01 | 0,78 | 4,6E-07 | 3,6E-06 | 0,52 |
| *NAB2* | 268,8 | 275,0 | 205,5 | 157,0 | 75,4 | 8,9E-01 | 9,5E-01 | 0,98 | 2,6E-01 | 4,9E-01 | 1,31 | 1,4E-02 | 5,2E-02 | 1,71 | 8,4E-13 | 2,0E-11 | 3,57 |
| *SUCLA2* | 210,3 | 207,4 | 319,6 | 314,9 | 391,0 | 9,0E-01 | 9,5E-01 | 1,01 | 4,4E-03 | 2,1E-02 | 0,66 | 1,9E-03 | 1,1E-02 | 0,67 | 1,3E-09 | 1,9E-08 | 0,54 |
| *SDPR* | 24,6 | 23,6 | 25,0 | 102,3 | 107,1 | 9,0E-01 | 9,5E-01 | 1,04 | 9,6E-01 | 9,7E-01 | 0,98 | 1,4E-04 | 1,3E-03 | 0,24 | 3,8E-08 | 3,8E-07 | 0,23 |
| *ME2* | 39,3 | 39,8 | 43,8 | 49,5 | 47,6 | 9,1E-01 | 9,6E-01 | 0,99 | 5,0E-01 | 7,4E-01 | 0,90 | 1,1E-01 | 2,3E-01 | 0,80 | 5,9E-02 | 1,2E-01 | 0,83 |
| *SMOX* | 4,7 | 4,7 | 5,4 | 4,8 | 4,7 | 9,3E-01 | 9,7E-01 | 1,00 | 1,1E-02 | 4,5E-02 | 0,88 | 6,5E-01 | 8,3E-01 | 0,99 | 8,5E-01 | 8,9E-01 | 1,00 |
| *NSUN7* | 4,7 | 4,7 | 4,8 | 4,7 | 4,7 | 9,3E-01 | 9,7E-01 | 1,00 | 1,4E-02 | 5,3E-02 | 0,98 | 7,4E-01 | 8,6E-01 | 1,00 | 3,8E-01 | 5,0E-01 | 1,00 |
| *AQP1* | 131,1 | 133,7 | 129,8 | 184,3 | 144,2 | 9,3E-01 | 9,7E-01 | 0,98 | 9,7E-01 | 9,8E-01 | 1,01 | 2,3E-01 | 4,1E-01 | 0,71 | 5,8E-01 | 6,8E-01 | 0,91 |
| *THRB* | 4,7 | 4,7 | 4,7 | 4,7 | 4,7 | 9,4E-01 | 9,8E-01 | 1,00 | 5,2E-01 | 7,6E-01 | 1,00 | 8,2E-01 | 8,9E-01 | 1,00 | 7,6E-01 | 8,3E-01 | 1,00 |
| *GRP* | 5,8 | 5,8 | 5,0 | 5,2 | 6,6 | 9,4E-01 | 9,8E-01 | 1,01 | 4,6E-01 | 7,1E-01 | 1,15 | 4,8E-01 | 6,8E-01 | 1,13 | 5,9E-01 | 6,9E-01 | 0,89 |
| *TRIM9* | 4,7 | 4,7 | 4,7 | 4,7 | 4,7 | 9,4E-01 | 9,8E-01 | 1,00 | 6,2E-01 | 8,4E-01 | 1,00 | 5,5E-01 | 7,4E-01 | 1,00 | 5,3E-01 | 6,4E-01 | 1,00 |
| *LAP3* | 855,8 | 845,7 | 571,6 | 535,8 | 866,4 | 9,4E-01 | 9,8E-01 | 1,01 | 5,9E-02 | 1,7E-01 | 1,50 | 1,3E-02 | 4,6E-02 | 1,60 | 9,3E-01 | 9,3E-01 | 0,99 |
| *DUSP2* | 21,4 | 20,9 | 9,8 | 9,4 | 15,2 | 9,4E-01 | 9,8E-01 | 1,02 | 6,8E-02 | 1,8E-01 | 2,18 | 2,8E-02 | 7,9E-02 | 2,27 | 2,2E-01 | 3,3E-01 | 1,41 |
| *INPP5J* | 160,9 | 157,2 | 271,3 | 474,3 | 318,3 | 9,5E-01 | 9,8E-01 | 1,02 | 2,8E-01 | 5,1E-01 | 0,59 | 7,3E-03 | 3,1E-02 | 0,34 | 8,3E-03 | 2,2E-02 | 0,51 |
| *PGLYRP4* | 4,9 | 4,9 | 5,1 | 4,8 | 4,7 | 9,5E-01 | 9,8E-01 | 1,00 | 5,9E-01 | 8,1E-01 | 0,96 | 7,2E-01 | 8,5E-01 | 1,02 | 2,9E-01 | 4,2E-01 | 1,04 |
| *COMP* | 50,1 | 51,5 | 7,2 | 8,0 | 5,1 | 9,6E-01 | 9,8E-01 | 0,97 | 4,8E-03 | 2,2E-02 | 6,93 | 2,1E-03 | 1,1E-02 | 6,28 | 2,1E-07 | 1,8E-06 | 9,76 |
| *ACTR3B* | 11,6 | 11,6 | 11,7 | 12,0 | 11,4 | 9,6E-01 | 9,8E-01 | 1,00 | 8,6E-01 | 9,1E-01 | 0,99 | 3,2E-01 | 5,3E-01 | 0,96 | 6,2E-01 | 7,1E-01 | 1,01 |
| *ADM* | 272,2 | 267,8 | 301,9 | 310,6 | 98,4 | 9,6E-01 | 9,8E-01 | 1,02 | 8,0E-01 | 8,8E-01 | 0,90 | 7,5E-01 | 8,6E-01 | 0,88 | 5,3E-05 | 2,6E-04 | 2,77 |
| *CHI3L1* | 991,8 | 965,9 | 17,0 | 80,9 | 40,9 | 9,6E-01 | 9,8E-01 | 1,03 | 1,2E-07 | 2,5E-06 | 58,43 | 2,2E-04 | 1,8E-03 | 12,25 | 6,6E-12 | 1,3E-10 | 24,27 |
| *NCS1* | 5,1 | 5,0 | 6,7 | 5,2 | 5,6 | 9,6E-01 | 9,8E-01 | 1,00 | 2,7E-02 | 8,9E-02 | 0,76 | 6,3E-01 | 8,1E-01 | 0,97 | 2,6E-02 | 6,2E-02 | 0,91 |
| *WISP1* | 4,7 | 4,7 | 4,7 | 4,7 | 4,7 | 9,7E-01 | 9,9E-01 | 1,00 | 5,1E-01 | 7,6E-01 | 1,01 | 4,1E-01 | 6,1E-01 | 1,01 | 5,1E-01 | 6,2E-01 | 1,01 |
| *SUOX* | 58,6 | 58,7 | 98,4 | 79,3 | 69,0 | 9,7E-01 | 9,9E-01 | 1,00 | 1,4E-07 | 2,7E-06 | 0,60 | 5,2E-03 | 2,4E-02 | 0,74 | 6,0E-03 | 1,6E-02 | 0,85 |
| *SYK* | 171,6 | 172,1 | 160,1 | 211,0 | 241,6 | 9,8E-01 | 1,0E+00 | 1,00 | 6,5E-01 | 8,5E-01 | 1,07 | 1,4E-01 | 2,8E-01 | 0,81 | 5,3E-04 | 2,0E-03 | 0,71 |
| *RXRG* | 103,5 | 102,6 | 125,1 | 54,2 | 11,1 | 9,8E-01 | 1,0E+00 | 1,01 | 7,3E-01 | 8,7E-01 | 0,83 | 2,1E-01 | 4,0E-01 | 1,91 | 1,3E-09 | 1,9E-08 | 9,35 |
| *NPY* | 4,8 | 4,8 | 4,7 | 4,8 | 4,7 | 9,9E-01 | 1,0E+00 | 1,00 | 6,5E-01 | 8,5E-01 | 1,02 | 9,4E-01 | 9,6E-01 | 1,00 | 3,0E-01 | 4,3E-01 | 1,03 |
| *PRRX2* | 8,0 | 7,9 | 7,8 | 7,6 | 7,3 | 9,9E-01 | 1,0E+00 | 1,00 | 7,8E-01 | 8,7E-01 | 1,02 | 4,4E-01 | 6,4E-01 | 1,05 | 5,3E-02 | 1,1E-01 | 1,09 |
| *MPC1* | 112,7 | 112,8 | 120,2 | 154,8 | 183,3 | 9,9E-01 | 1,0E+00 | 1,00 | 6,7E-01 | 8,5E-01 | 0,94 | 7,2E-03 | 3,1E-02 | 0,73 | 3,2E-07 | 2,6E-06 | 0,61 |
| *GFRA4* | 4,7 | 4,7 | 5,0 | 4,9 | 5,2 | 9,9E-01 | 1,0E+00 | 1,00 | 1,3E-03 | 8,7E-03 | 0,94 | 3,9E-03 | 1,9E-02 | 0,97 | 2,5E-01 | 3,7E-01 | 0,91 |
| *PVR* | 4,7 | 4,7 | 4,7 | 4,8 | 4,8 | 9,9E-01 | 1,0E+00 | 1,00 | 4,1E-01 | 6,7E-01 | 0,99 | 3,3E-02 | 9,2E-02 | 0,97 | 3,1E-01 | 4,3E-01 | 0,98 |
| *SV2A* | 27,8 | 27,8 | 79,8 | 81,3 | 18,0 | 1,0E+00 | 1,0E+00 | 1,00 | 8,1E-03 | 3,4E-02 | 0,35 | 2,4E-03 | 1,2E-02 | 0,34 | 4,6E-02 | 1,0E-01 | 1,54 |
| *OPTN* | 174,9 | 174,9 | 139,4 | 126,3 | 148,1 | 1,0E+00 | 1,0E+00 | 1,00 | 2,9E-01 | 5,2E-01 | 1,25 | 7,8E-02 | 1,8E-01 | 1,38 | 1,9E-01 | 3,0E-01 | 1,18 |
