# Supplementary material for: BRAFV600E-Associated Gene Expression Profile: Early Changes in the Transcriptome, Based on a Transgenic Mouse Model of Papillary Thyroid Carcinoma
Source: PLoS One. 2015 Dec 1;10(12):e0143688. doi: 10.1371/journal.pone.0143688 (PMC4666467; doi:10.1371/journal.pone.0143688)
Supplement: S3 Table — The results are summarized with human analysis of BRAF(+) PTCs versus healthy thyroids. a–tested for differences by U Mann-Whitney test (remaining columns were earlier selected for significance, thus are not tested here). b–denotes differences statistically significant at p-value p<0.05 (U Mann-Whitney test). (DOC) [file pone.0143688.s010.doc]

**S3 Table Comparison of *BRAF*(+) as well as *BRAF*(-) mouse thyroid lesions (including healthy thyroids, hyperplastic lesions, borderline lesions and PTC) to *BRAF*(-) asymptomatic thyroids for 18 genes designated as *BRAF*V600E-dependent. The results are summarized with human analysis of *BRAF*(+) PTCs versus healthy thyroids.**

| **Gene** | **Fold-change of geometric means^a^** | | | | | |
| --- | --- | --- | --- | --- | --- | --- |
|  | **in mouse thyroids** | | | | | **in human thyroids** |
|  | ***BRAF*(+) healthy  vs *BRAF*(-) healthy** | ***BRAF*(-)  hyperplastic lesion vs *BRAF*(-) healthy** | ***BRAF*(+)  hyperplastic lesion vs *BRAF*(-) healthy** | ***BRAF*(+)  borderline lesion  vs *BRAF*(-) healthy** | ***BRAF*(+) PTC vs *BRAF*(-) healthy** | ***BRAF*(+) PTC vs  normal thyroid** |
| *DCSTAMP* | 1.45 | 1.00 | 1.96 | 1.64^b^ | 2.05^b^ | 61.86^b^ |
| *SLC34A2* | 3.15 | 0.96 | 4.72 | 4.52^b^ | 6.36^b^ | 31.99^b^ |
| *FN1* | 1.56 | 1.22 | 3.19 | 2.27^b^ | 2.72^b^ | 18.87^b^ |
| *PDLIM4* | 1.44 | 0.83 | 2.14 | 1.84^b^ | 2.28^b^ | 10.69^b^ |
| *MET* | 1.31 | 1.11 | 1.98 | 1.69^b^ | 2.01^b^ | 9.09^b^ |
| *ERBB3* | 1.39 | 1.17 | 2.17 | 1.91^b^ | 2.23^b^ | 5.13^b^ |
| *LAD1* | 1.41 | 1.04 | 1.96 | 1.66^b^ | 1.76^b^ | 3.22^b^ |
| *EPHA2* | 1.41 | 0.99 | 2.17 | 1.89^b^ | 2.26^b^ | 2.98^b^ |
| *PLAUR* | 1.69 | 1.04 | 2.46 | 1.89^b^ | 2.21^b^ | 2.92^b^ |
| *ITPR3* | 1.52 | 1.10 | 2.56 | 2.13^b^ | 2.49^b^ | 2.24^b^ |
| *GRB7* | 1.46 | 1.18 | 1.76 | 1.69^b^ | 1.84^b^ | 2.23^b^ |
| *RASA1* | 1.10 | 1.04 | 2.04 | 1.59^b^ | 1.97^b^ | 2.14^b^ |
| *ALDH3B1* | 1.65 | 1.23 | 2.13 | 1.94^b^ | 2.11^b^ | 2.07^b^ |
| *AACS* | 0.89 | 1.06 | 0.61 | 0.64^b^ | 0.66^b^ | 0.59^b^ |
| *MMD* | 0.82 | 1.19 | 0.52 | 0.65^b^ | 0.67^b^ | 0.54^b^ |
| *PVRL3* | 1.52 | 1.03 | 2.08 | 1.71^b^ | 2.09^b^ | 0.32^b^ |
| *IQGAP2* | 0.63 | 0.86 | 0.43 | 0.59^b^ | 0.50^b^ | 0.19^b^ |
| *DIO1* | 0.60 | 0.90 | 0.12 | 0.29^b^ | 0.23^b^ | 0.02^b^ |

a – tested for differences by U Mann-Whitney test (remaining columns were earlier selected for significance, thus are not tested here)

b – denotes differences statistically significant at p-value p<0.05 (U Mann-Whitney test)
