# Supplementary material for: BRAFV600E-Associated Gene Expression Profile: Early Changes in the Transcriptome, Based on a Transgenic Mouse Model of Papillary Thyroid Carcinoma
Source: PLoS One. 2015 Dec 1;10(12):e0143688. doi: 10.1371/journal.pone.0143688 (PMC4666467; doi:10.1371/journal.pone.0143688)
Supplement: S4 Table — a- denotes differences statistically significant at FDR<0.05 (U Mann-Whitney test). (DOC) [file pone.0143688.s011.doc]

**S4 Table Comparison of human *RET*(+), *RAS*(+) and PTC(-) to healthy thyroids**

|  | ***RET*(+) vs healthy** | ***RAS*(+) vs healthy** | **PTC(-) vs healthy** | ***BRAF* vs healthy** |
| --- | --- | --- | --- | --- |
| **Symbol** | **Fold change** | **Fold change** | **Fold change** | **Fold change** |
| *DCSTAMP* | 2,52^a^ | 0,34^a^ | 0,38^a^ | 61,86^a^ |
| *MMD* | 1,26 | 2,90^a^ | 1,33 | 0,54^a^ |
| *SLC34A2* | 10,13^a^ | 0,51 | 1,61 | 31,99^a^ |
| *FN1* | 3,38^a^ | 0,30^a^ | 0,42 | 18,87^a^ |
| *PLAUR* | 10,47^a^ | 0,80 | 0,73 | 2,92^a^ |
| *ITPR3* | 1,32 | 1,09 | 1,03 | 2,24^a^ |
| *GRB7* | 1,27^a^ | 1,10 | 1,16 | 2,23^a^ |
| *PDLIM4* | 6,42^a^ | 0,64 | 0,85 | 10,69^a^ |
| *MET* | 4,46^a^ | 1,18 | 2,02 | 9,09^a^ |
| *AACS* | 0,88 | 1,36 | 1,37 | 0,59^a^ |
| *RASA1* | 1,48^a^ | 1,10 | 1,09 | 2,14^a^ |
| *LAD1* | 1,86^a^ | 1,27 | 1,17 | 3,22^a^ |
| *IQGAP2* | 0,42^a^ | 2,00^a^ | 0,80 | 0,19^a^ |
| *ERBB3* | 2,06 | 0,99 | 1,77 | 5,13^a^ |
| *PVRL3* | 0,72 | 1,03 | 1,38 | 0,32^a^ |
| *EPHA2* | 4,88^a^ | 1,05 | 0,93 | 2,98^a^ |
| *DIO1* | 0,07^a^ | 2,05 | 0,53 | 0,02^a^ |
| *ALDH3B1* | 1,35^a^ | 1,09 | 1,23 | 2,07^a^ |

a- denotes differences statistically significant at FDR<0.05 (U Mann-Whitney test)
